# Supplementary material for: Dementia and disadvantage in the USA and England: population-based comparative study
Source: BMJ Open. 2021 Oct 5;11(10):e045186. doi: 10.1136/bmjopen-2020-045186 (PMC8496387; doi:10.1136/bmjopen-2020-045186)
Supplement: Supplementary data [file bmjopen-2020-045186supp001.pdf]

Supplementary Appendix (For Online Publication)

Research in context

We reviewed existing evidence in July 2020, searching PubMed database for any studies looking at cross-country comparisons of dementia using the same standard measures and diagnostic practices.

("Dementia"[mesh]) AND ("Prevalence"[mesh])

AND

(“Survey”[tw]) AND (“Population”[tw]) AND (“Representative”[tw])

AND

(“Cross-Country”[ti] OR “Comparison”[ti])

This search yielded no results. The only relevant project we could find relating to our research was regarding the Health and Retirement Study Harmonized Cognitive Assessment Protocol (HCAP) Project. [1] This is an international research collaboration funded by the National Institute on Aging to better measure and identify cognitive impairment and dementia in longitudinal studies of countries around the world. However, this project has not yet released any international comparisons.

We also reviewed existing evidence in the PubMed database for any studies looking at how dementia prevalence varies by socioeconomic status (SES) in either England or the US. The search terms used were the following:

("Dementia"[mesh]) AND

("Prevalence"[tw]) AND ("Socioeconomic"[ti] OR "Socioeconomic"[tw] OR "SES"[tw]) AND

(“United Kingdom”[mesh] OR “United Kingdom”[tw] OR “England”[tw] OR “UK”[tw] OR “United Kingdom”[tw] "Britain"[tw] OR “United States”[mesh] OR “United States”[tw] OR “US”[tw] OR “America”[tw])

This search yielded 50 results. Papers which were not relevant were manually removed. We also excluded papers that did not use nationally representative samples. We performed additional searches using lists of references retrieved from relevant papers. Relevant papers found in the search can be found in Table A1 below.

Table A1: Studies on Dementia Prevalence by Socioeconomic Status (SES)

Authors	Country	Socioeconomic Indicator	Years	Findings
Langa et al. (2017)[1]	United States	Education	2000 and 2012	Link between education and dementia risk
Hudomiet et al, (2019)[2]	United States	Education and Social Security Benefits	1998 to 2014	Link between education and dementia risk
Rusmaully et al, (2017)[3]	United Kingdom	Education, height, occupational position	Various years between 1985 and 2015	High cognitive reserve associated with lower risk for dementia
Cadar et al, (2018)[4]	United Kingdom	Education, wealth and the index of multiple deprivation	2002 to 2015	Lower wealth in but not education associated with increased risk for dementia
Rocca et al,(2011)[5]	United States	Education, net worth	1993 and 2002	Higher education, higher net worth protected against cognitive impairment.
Basu (2013)[6]	United States	Education	2000 - 2002	Education has causal effect on dementia risk
Nguyen et al, (2016)[7]	United States	Education	1998 to 2010	Education protective against dementia risk
Crimmins et al, (2018)[8]	United States	Education	2000 and 2010	More education linked to lower dementia prevalence
Garcia et al, (2018)[9]	United States	Race, ethnicity, nativity, and education	2012	Education reduces the odds for CIND
Weden et al, (2018)[10]	United States	Race, ethnicity, total number of children, marital status, highest educational attainment, and net total assets in 2000	2000 and 2010	Strong protective role of educational attainment and persisting rural disadvantages for dementia

Arapakis K, *et al. BMJ Open* 2022; 11:e045186. doi: 10.1136/bmjopen-2020-045186

Data Appendix

HRS Data

The Health and Retirement Study (HRS) is a nationally representative, biennial longitudinal survey of adults in the United States.[11] It started in 1992 and since it collects a wide range of questions on income, wealth, employment, health, cognition, and demographics. It utilises a steady-state sampling design, with a new cohort aged 51-56 entering every 6 years. In total, 43,478 individuals have been interviewed to date. We used data from the 2016 and earlier waves to predict dementia and focused on the over the age of 70 years old that live in the community or in nursing homes in 2016. This left a sample of 7,165 individuals. We restricted our attention to non-Hispanic whites for comparability with ELSA. These restrictions generated a main study sample of 5,330 participants with 4,932 being self-respondents, and 398 proxy interviews.

ELSA Data

The English Longitudinal Study of Ageing (ELSA) is a biennial longitudinal survey of adults in England, developed as a companion study to the HRS.[12] ELSA was also designed to be nationally representative of the non-institutionalised population. Respondents remain the study if they become institutionalised. While it is has been shown to be representative of the English population in terms of sociodemographic characteristics, the proportion of non-white people in the survey is very small.[12] We used data from the 2016 and earlier waves and focused on the aged over 70 years old that live in the community or in nursing homes in 2016. This left a sample of 3,224 individuals. We restricted our attention to non-Hispanic whites for comparability. These restrictions generated a study sample of 3,147 participants with 3,007 being self-respondents, and 140 proxy interviews.

Harmonised Variables HRS and ELSA

We use the same set of cognitive and demographic variables available in both the HRS and ELSA.

For cognitive and demographic measures, we used the same questions as Hurd et al.[13] Specifically, we used variables on demographics, difficulties respondents have with Activities of Daily Living (ADLs), Instrumental Activities of Daily Living (IADLs), and a range of cognitive function tests adapted from the Telephone Interview for Cognitive Status (TICS) and their change across waves. For Proxy respondents we also used the shortened 16 question form of IQCODE.

We used four variables to measure socioeconomic status (SES). These are household income, education, total household wealth, and total household non-housing wealth. For the HRS we used variables from HRS RAND dataset. To establish each respondent's SES in terms of income, wealth, and non-housing wealth, we assigned everyone to a decile rank for each variable in their respective country. For education, we ranked respondents according to their number of years of schooling. We used respondents' reported wealth and non-housing wealth from 4 years prior. If a respondent had a missing value of income, wealth, or non-housing wealth we used the most recent non-missing observation from previous waves. All amounts of income, wealth and non-housing wealth were deflated to 2016 GBPs, and USD amounts were converted to GBPs using 1 USD = 0.75 GBP.

Finally, in our analysis, we investigated how variables that have been associated with dementia affect our results. These include Body Mass Index (BMI), past smoking behaviour, past stroke. Respondents are asked whether they have any health conditions, of which we use whether they had any cardiometabolic diseases (diabetes, heart disease, and stroke) and/or psychiatric conditions 4 years prior. BMI is collected by a nurse for every ELSA respondent when they first enter the survey, which for everyone over 70 in the 2016 survey means their BMI was collected over 10 years prior. BMI is recorded in each wave of HRS based on self-reported height and weight. To make the BMI measures in HRS comparable with ELSA, we used respondents first ever recorded BMI in HRS, which for our sample was at least 10 years prior.

Hurd et al. Dementia Prediction Algorithm

To estimate the probability of dementia for each individual present in our pooled data we follow the method of Hurd et al. that is based on the HRS supplement Aging, Demographics and Memory Study (ADAMS).[13] It is a representative subsample of HRS members aged over 70 that received a detailed in-home assessment of their cognitive status by experienced teams at the Duke University Dementia Epidemiology

Research Center.[14] Consensus conferences were used to establish a final diagnosis of dementia or a cognitive impairment with no dementia (CIND) for each participant. Hurd et al. applied separate ordered probit models to self- and proxy-respondents to generate a predictive algorithm, based on the ADAMS diagnoses, for the whole HRS sample, using a range of variables including demographic information, ADLs, IADLs, the TICS questionnaire, IQCODE, lagged variables and differences in variables between waves. The algorithm predicts the probability of dementia in the following year.

Most of the coefficients used in the predictive algorithm can be found in the online appendix of the Hurd et al. paper. To account for the small number of missing variables in HRS and ELSA we either used coefficients provided by the authors or estimated them by a conditional minimum distance estimator using the publicly available HRS data and the predicted values of the researcher contribution Dementia Predicted Probability Files (DPPF), that follows the method of Hurd et al. To verify our predictions in HRS, we compared our predictions with the DPPF data available on the HRS website for the year 2008 and found that we accurately matched predicted dementia.

Hurd et al's predictive algorithm has been shown to have high specificity and accuracy, and generally performs well in terms of sensitivity compared to other predictive algorithms for dementia.[15]

Table A2: HRS and ELSA Response Rates

Year	2010	2012	2014	2016
HRS				
Wave	10	11	12	13
Response rate (%)	81.0	89.1	87.1	84.3
ELSA				
Wave	5	6	7	8
Fieldwork response rate (%)	79.1	80.2	80.1	82.4

Notes: The response rates are calculated differently in HRS and ELSA. For the HRS, the response rate includes all individuals who were determined to be eligible for HRS who completed a baseline interview.[16] For ELSA, the fieldwork response rate is the proportion of eligible survey units who participate in the research study, where 'eligible' means not having been found to be ineligible through death or moving out of Great Britain.[17]

Table A3: Summary Statistics, Raw Sample, England vs United States

	Raw Full Sample		With Population Weights	
	Non-Standardized Mean		Non-Standardized Mean	
	England	United States	England	United States
Total Sample Size	3,147	5,330	3,147	5,330
Proxy Respondents (%)	0.044	0.075	0.050	0.072
Age	78.8	80.5	79.5	79.7
Female	0.551	0.590	0.553	0.561
Married	0.580	0.517	0.584	0.536
Current Income and Wealth (£)				
Income	21,070	28,800	20,215	30,240
Wealth	280,000	210,000	270,000	230,000
Non-Housing Wealth	40,000	88,275	36,000	96,150
Wealth 4 Years Prior (£)				
Wealth	270,000	220,000	250,000	230,000
Non-Housing Wealth	42,300	96,800	35,727	100,000
Education				
Less than High School	0.362	0.174	0.424	0.161
High-school or Some College	0.473	0.573	0.444	0.560
College	0.165	0.253	0.132	0.279
Current Health				
ADLs (Out of 6)	0.510	0.583	0.561	0.548
IADLs (Out of 5)	0.348	0.513	0.411	0.482
Arthritis	0.569	0.728	0.578	0.715
Cancer	0.190	0.251	0.189	0.244
Lung disease	0.117	0.135	0.118	0.127
Diabetes	0.162	0.246	0.166	0.240
Heart Disease	0.390	0.383	0.396	0.365
High Blood Pressure	0.576	0.684	0.583	0.671
Psychiatric Condition	0.134	0.171	0.132	0.175
Stroke	0.092	0.103	0.096	0.095
Health 4 Years Prior				
ADLs	0.346	0.274	0.381	0.267
IADLs	0.159	0.228	0.185	0.219
Diabetes	0.131	0.215	0.135	0.212
Heart Disease	0.309	0.316	0.312	0.303
High Blood Pressure	0.513	0.653	0.519	0.643
Psychiatric Condition	0.120	0.157	0.118	0.162
Stroke	0.063	0.074	0.065	0.067
Other Health Factors				
Ever Smoked	0.660	0.553	0.674	0.561
BMI: <20 (Underweight)	0.016	0.038	0.015	0.036
BMI: 20-24.9 (Normal Weight)	0.266	0.352	0.262	0.343
BMI: 25-29.9 (Overweight)	0.461	0.405	0.461	0.410
BMI: 30+ (Obese)	0.256	0.205	0.262	0.211

Notes: Sample includes non-Hispanic white population aged 70+ only. The sample size is participants in England and participants in the United States. The mean is presented for each variable, apart from income and wealth variables where medians values are displayed. All amounts deflated to 2016 £s. Dollar amounts converted to £ using 1 $ = 0.75 £. Age is top coded at 99.

Table A4: Summary Statistics, Age-Gender Standardized, England vs United States

	Full Sample: Age-Gender Standardized Mean		Lowest Income Decile: Age-Gender Standardized Mean	
	England	United States	England	United States
Age	79.5	79.3	79.7	79.7
Female	0.553	0.553	0.553	0.553
Married	0.584	0.547	0.148	0.170
Current Income and Wealth (£)				
Income	20,547	31,927	8,247	8,645
Wealth	271,511	236,431	120,261	46,762
Non-Housing Wealth	38,715	100,996	7,778	5,509
Wealth 4 Years Prior (£)				
Wealth	257,459	242,655	111,830	55,953
Non-Housing Wealth	40,604	110,530	6,476	13,724
Education				
Less than High School	0.424	0.160	0.619	0.323
High-school or Some College	0.444	0.559	0.318	0.556
College	0.132	0.281	0.063	0.122
Current Health				
ADLs (Out of 6)	0.561	0.523	0.559	0.827
IADLs (Out of 5)	0.411	0.457	0.421	0.679
Arthritis	0.578	0.715	0.617	0.750
Cancer	0.189	0.244	0.157	0.196
Lung disease	0.118	0.127	0.137	0.217
Diabetes	0.166	0.242	0.157	0.283
Heart Disease	0.396	0.364	0.402	0.427
High Blood Pressure	0.583	0.671	0.619	0.703
Psychiatric Condition	0.132	0.174	0.158	0.262
Stroke	0.096	0.094	0.111	0.135
Health 4 Years Prior				
ADLs	0.381	0.255	0.424	0.546
IADLs	0.185	0.205	0.218	0.393
Diabetes	0.135	0.214	0.131	0.254
Heart Disease	0.312	0.302	0.345	0.352
High Blood Pressure	0.519	0.640	0.559	0.662
Psychiatric Condition	0.118	0.162	0.142	0.271
Stroke	0.065	0.066	0.064	0.099
Other Health Factors				
Ever Smoked	0.674	0.565	0.741	0.594
BMI: <20 (Underweight)	0.015	0.036	0.025	0.032
BMI: 20-24.9 (Normal Weight)	0.261	0.342	0.361	0.364
BMI: 25-29.9 (Overweight)	0.461	0.410	0.397	0.339
BMI: 30+ (Obese)	0.262	0.212	0.216	0.265

Notes: Sample includes non-Hispanic white population aged 70+ only. The sample size is 3,147 participants in England and 5,330 participants in the United States. The age-gender standardized mean is presented for each variable, apart from income and wealth variables where age-gender standardised medians values are displayed. The standard population is the overall age 70+ population in England in 2016. All amounts deflated to 2016 £s. Dollar amounts converted to £ using 1 \$ = 0.75 £. Age is top coded at 99.

Table A5: Summary Statistics for Predictor Variables, England vs United States

	Full Sample		Lowest Income Decile	
	Age-Gender- Standardized Mean		Age-Gender-Standardized Mean	
	England	United States	England	United States
**Cognitive Scores**				
Dates (Out of 4)	3.663	3.387	3.573	3.198
Backward counting 20	0.912	0.875	0.935	0.830
Serial 7 (Out of 5)	3.787	3.364	3.667	2.580
Scissor	0.978	0.920	0.993	0.912
Cactus	0.915	0.891	0.913	0.842
PM/Vice-President	0.846	0.900	0.828	0.854
Immediate recall (Out of 10)	5.318	4.631	5.010	3.972
Delayed recall (Out of 10)	3.797	3.684	3.185	2.894
**Limitations**				
ADLs (Out of 6)	0.561	0.523	0.559	0.827
IADLs (Out of 5)	0.411	0.457	0.421	0.679
Proxy Respondent	0.050	0.068	0.038	0.075

Notes: Sample includes non-Hispanic white population aged 70+ only. The sample size is 3,147 participants in England and 5,330 participants in the United States. The Cognitive Scores are from a range of tests adapted from Telephone Interview for Cognitive Status (TICS). Only non-proxy respondents answered the TICS questions. ADL and IADLs are Activities of Daily Living and Instrumental Activities of Daily Living, respectively.

Table A6: Indexes of Inequality

	Slope Index of Inequality (SII)			Relative Index of Inequality (RII)		
	England	United States	Difference	England	United States	Difference
Income	-0.062	-0.085	-0.023	1.946	2.341	1.203
	[-0.097 to -0.028]	[-0.114 to -0.057]	(0.31)	[1.333 to 2.841]	[1.774 to 3.089]	(0.44)
Education	-0.087	-0.099	-0.011	3.001	2.820	0.940
	[-0.120 to -0.055]	[-0.126 to -0.072]	(0.56)	[2.013 to 4.474]	[2.131 to 3.731]	(0.80)
Wealth	-0.069	-0.096	-0.026	2.157	2.730	1.265
	[-0.104 to -0.035]	[-0.123 to -0.069]	(0.24)	[1.467 to 3.174]	[2.070 to 3.601]	(0.33)
NH Wealth	-0.071	-0.093	-0.022	2.213	2.311	1.044
	[-0.106 to -0.035]	[-0.124 to -0.062]	(0.36)	[1.463 to 3.348]	[1.748 to 3.056]	(0.87)
**Excluding Most Disadvantaged Group**						
Income	-0.067	-0.060	0.007	2.104	1.833	0.871
	[-0.107 to -0.027]	[-0.093 to -0.027]	(0.79)	[1.343 to 3.296]	[1.313 to 2.560]	(0.63)
Education	-0.070	-0.062	0.008	2.509	1.932	0.770
	[-0.107 to -0.032]	[-0.094 to -0.030]	(0.76)	[1.548 to 4.065]	[1.378 to 2.708]	(0.39)
Wealth	-0.065	-0.059	0.005	2.092	1.887	0.902
	[-0.104 to -0.025]	[-0.091 to -0.028]	(0.84)	[1.319 to 3.317]	[1.349 to 2.639]	(0.72)
NH Wealth	-0.064	-0.075	-0.012	2.077	1.981	0.954
	[-0.107 to -0.020]	[-0.110 to -0.040]	(0.69)	[1.217 to 3.544]	[1.433 to 2.740]	(0.88)
**Full Sample with Controls**						
Income	-0.040	-0.051	-0.011	1.499	1.698	1.133
	[-0.075 to -0.004]	[-0.079 to -0.023]	(0.63)	[1.028 to 2.187]	[1.275 to 2.263]	(0.61)
Education	-0.0336	-0.056	-0.022	1.308	1.742	1.332
	[-0.0754 to 0.008]	[-0.086 to -0.026]	(0.40)	[0.899 to 1.903]	[1.306 to 2.324]	(0.24)
Wealth	-0.060	-0.045	0.015	1.777	1.483	0.834
	[-0.097 to -0.023]	[-0.074 to -0.015]	(0.53)	[1.224 to 2.582]	[1.131 to 1.945]	(0.44)
NH Wealth	-0.050	-0.051	-0.002	1.562	1.614	1.033
	[-0.087 to -0.012]	[-0.080 to -0.023]	(0.94)	[1.078 to 2.262]	[1.231 to 2.116]	(0.89)

Notes: This table shows the Slope Index of Inequality (SII) and Relative Index of Inequality (RII), calculated using generalized linear models (log binomial regression) with identity and logarithmic link functions, respectively. The SIIs are displayed graphically in Figure 1. The estimates of RII are risk ratios. P-values are displayed in parentheses under differences. The panel 'Excluding Most Disadvantaged Group' excludes individuals in the bottom income decile. The panel 'Full Sample with Controls' controls for whether an individual had any cardiometabolic diseases (diabetes, heart disease, and stroke) and/or psychiatric conditions 4 years prior, whether an individual has ever smoked, and Body Mass Index (BMI) from at least 10 years prior.

Table A7: Difference in Prevalence of Dementia with Controls, United States vs England, 2017

Whole Sample:					
England Prevalence	0.097	0.088	0.086	0.089	0.092
	(0.089 to 0.106)	(0.078 to 0.098)	(0.073 to 0.099)	(0.072 to 0.106)	(0.072 to 0.111)
United States	0.112	0.100	0.098	0.101	0.120
	(0.106 to 0.118)	(0.092 to 0.108)	(0.087 to 0.109)	(0.087 to 0.114)	(0.104 to 0.135)
Percentage Point Difference	1.43	1.15	1.21	1.18	2.82
p-value	0.0055	0.025	0.020	0.034	<0.0001
Excluding Lowest Income Decile:					
England Prevalence	0.094	0.086	0.086	0.090	0.094
	(0.085 to 0.103)	(0.076 to 0.096)	(0.073 to 0.1)	(0.073 to 0.108)	(0.074 to 0.114)
United States	0.103	0.093	0.094	0.098	0.115
	(0.097 to 0.109)	(0.085 to 0.102)	(0.083 to 0.105)	(0.084 to 0.113)	(0.099 to 0.131)
Percentage Point Difference	0.89	0.74	0.75	0.81	2.09
p-value	0.11	0.18	0.18	0.18	0.0021
Lowest Income Decile:					
England Prevalence	0.114	0.107	0.088	0.082	0.095
	(0.089 to 0.139)	(0.074 to 0.14)	(0.036 to 0.14)	(0.021 to 0.144)	(0.025 to 0.164)
United States	0.187	0.162	0.148	0.131	0.163
	(0.166 to 0.208)	(0.129 to 0.195)	(0.103 to 0.192)	(0.078 to 0.184)	(0.103 to 0.223)
Percentage Point Difference	7.27	5.45	5.93	4.85	6.85
p-value	<0.0001	0.0020	0.0011	0.012	0.0015
Control for:					
Past Health Conditions		✓	✓	✓	✓
Ever Smoked			✓	✓	✓
Past BMI				✓	✓
Education					✓

Notes: Sample includes non-Hispanic white population aged 70+ only. The sample size is 3,147 participants in England and 5,330 participants in the United States. All estimates are age-gender standardized to the overall 2016 aged 70+ white population in England. The difference is calculated as the prevalence in the US minus prevalence in England. Differences are displayed as percentage points. ‘Past Health Conditions’ controls for whether an individual had any cardiometabolic diseases (diabetes, heart disease, and stroke) and/or psychiatric conditions 4 years prior. ‘Ever Smoked’ controls for whether an individual has ever smoked. BMI stands for Body Mass Index. ‘Past BMI’ includes dummy variables to control for whether an individual is classed as underweight, normal weight, *overweight* or *obese*. BMI measurements are based on when an individual first entered the survey, which is at least 10 years prior. We assume the effect of each control is constant across countries and age/gender groups. The reference groups are: No past health conditions; never smoked; normal weight; and high-school education.

Table A8: Difference in Prevalence of Dementia with Controls, United States vs England, 2017

	England			United States		
Whole Sample:						
Prevalence	0.097	0.092	0.089	0.112	0.105	0.101
CI	(0.089 to 0.106)	(0.082 to 0.102)	(0.072 to 0.106)	(0.106 to 0.118)	(0.098 to 0.113)	(0.087 to 0.114)
Income Decile:						
1 *Prevalence*	0.114	0.112	0.082	0.187	0.173	0.131
CI	(0.089 to 0.139)	(0.079 to 0.144)	(0.021 to 0.144)	(0.166 to 0.208)	(0.142 to 0.205)	(0.078 to 0.184)
2 *Prevalence*	0.113	0.119	0.118	0.141	0.145	0.148
CI	(0.09 to 0.136)	(0.088 to 0.149)	(0.05 to 0.187)	(0.119 to 0.163)	(0.113 to 0.176)	(0.086 to 0.21)
3 *Prevalence*	0.124	0.107	0.130	0.111	0.089	0.093
CI	(0.097 to 0.151)	(0.074 to 0.139)	(0.073 to 0.187)	(0.095 to 0.127)	(0.067 to 0.11)	(0.048 to 0.137)
4 *Prevalence*	0.099	0.095	0.094	0.118	0.114	0.111
CI	(0.071 to 0.126)	(0.065 to 0.126)	(0.041 to 0.148)	(0.099 to 0.137)	(0.088 to 0.14)	(0.062 to 0.16)
5 *Prevalence*	0.094	0.087	0.061	0.086	0.084	0.063
CI	(0.072 to 0.116)	(0.062 to 0.112)	(0.024 to 0.098)	(0.069 to 0.102)	(0.063 to 0.104)	(0.035 to 0.09)
6 *Prevalence*	0.098	0.097	0.116	0.108	0.107	0.100
CI	(0.07 to 0.127)	(0.064 to 0.131)	(0.056 to 0.177)	(0.088 to 0.128)	(0.081 to 0.134)	(0.053 to 0.148)
7 *Prevalence*	0.068	0.066	0.073	0.100	0.101	0.104
CI	(0.042 to 0.093)	(0.038 to 0.094)	(0.021 to 0.124)	(0.078 to 0.122)	(0.073 to 0.129)	(0.054 to 0.154)
8 *Prevalence*	0.083	0.084	0.097	0.082	0.078	0.085
CI	(0.053 to 0.114)	(0.051 to 0.117)	(0.054 to 0.14)	(0.066 to 0.097)	(0.058 to 0.098)	(0.054 to 0.117)
9 *Prevalence*	0.082	0.076	0.100	0.093	0.085	0.107
CI	(0.041 to 0.122)	(0.034 to 0.118)	(0.048 to 0.151)	(0.071 to 0.116)	(0.06 to 0.111)	(0.07 to 0.145)
10 *Prevalence*	0.059	0.058	0.051	0.077	0.077	0.068
CI	(0.034 to 0.083)	(0.029 to 0.087)	(0.015 to 0.087)	(0.052 to 0.103)	(0.051 to 0.103)	(0.036 to 0.1)
Control for:						
Past Health Conditions		✓	✓		✓	✓
Ever Smoked			✓			✓
Past BMI			✓			✓

Notes: Sample includes non-Hispanic white population aged 70+ only. The sample size is 3,147 participants in England and 5,330 participants in the United States. All estimates are age-gender standardized to the overall 2016 aged 70+ white population in England. 'Past Health Conditions' controls for whether an individual had any cardiometabolic diseases (diabetes, heart disease, and stroke) and/or psychiatric conditions 4 years prior. 'Ever Smoked' controls for whether an individual has ever smoked. BMI stands for Body Mass Index. 'Past BMI' includes dummy variables to control for whether an individual is classed as underweight, normal weight, *overweight* or *obese*. BMI measurements are based on when an individual first entered the survey, which is at least 10 years prior. We assume the effect of each control is constant across countries and age/gender groups. The reference groups are: No past health conditions; never smoked; and normal weight.

Arapakis K, *et al. BMJ Open* 2022; 11:e045186. doi: 10.1136/bmjopen-2020-045186

Figure A1: SES Gradient of Dementia, US vs England, Controlling for Past Health

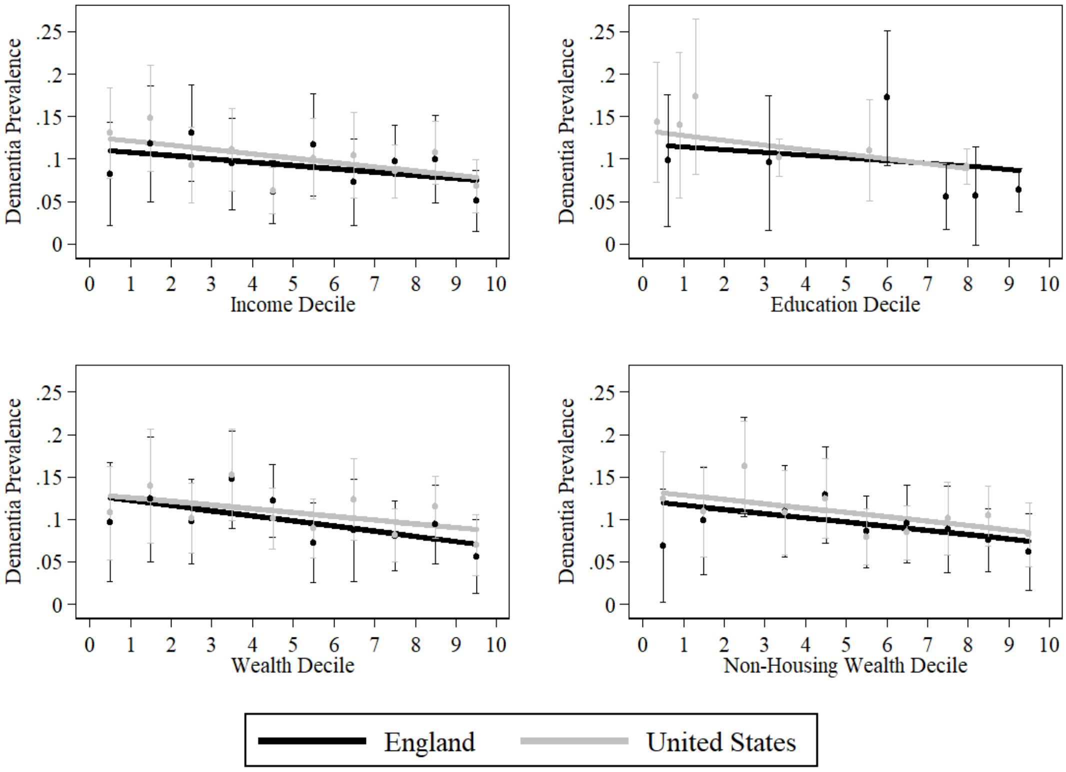

Notes: The points in this figure represent the mean age-gender standardized dementia prevalence for each country by socioeconomic status (SES) after controlling for past health and behaviour, along with 95% confidence interval for these predictions. The solid lines represent the fitted Socioeconomic Index of Inequality (SII) for each country. The values of the SII and the corresponding Relative Index of Inequality (RII) are listed in Table A4.

Table A9: Prevalence of Dementia, United States vs. England, Full-Sample

	England Age-Gender Standardized Prevalence [95% CI]		United States Age-Gender Standardized Prevalence [95% CI]		Difference	p-value
All	0.099	[0.091,0.108]	0.124	[0.118,0.129]	0.024	<0.0001
Household Income Decile						
1 (Lowest)	0.116	[0.091,0.140]	0.211	[0.192,0.230]	0.095	<0.0001
2	0.126	[0.101,0.152]	0.161	[0.144,0.178]	0.035	0.025
3	0.124	[0.098,0.150]	0.141	[0.120,0.162]	0.017	0.32
4	0.100	[0.072,0.127]	0.143	[0.124,0.162]	0.044	0.0094
5	0.101	[0.078,0.124]	0.097	[0.082,0.111]	-0.004	0.76
6	0.102	[0.072,0.131]	0.104	[0.086,0.121]	0.002	0.91
7	0.061	[0.043,0.079]	0.108	[0.088,0.127]	0.047	0.0006
8	0.088	[0.057,0.118]	0.085	[0.069,0.102]	-0.002	0.91
9	0.081	[0.041,0.122]	0.089	[0.070,0.108]	0.008	0.74
10 (Highest)	0.057	[0.034,0.080]	0.076	[0.055,0.098]	0.020	0.22
Years of Schooling						
9 or less	0.128	[0.103,0.153]	0.212	[0.195,0.230]	0.084	<0.0001
10	0.099	[0.078,0.119]	0.143	[0.118,0.167]	0.044	0.0072
11	0.097	[0.079,0.116]	0.119	[0.093,0.144]	0.021	0.19
12	0.069	[0.041,0.097]	0.134	[0.124,0.143]	0.064	<0.0001
13	0.063	[0.040,0.085]	0.118	[0.095,0.141]	0.055	0.00071
14 or more	0.055	[0.039,0.071]	0.086	[0.078,0.094]	0.031	0.00071
Household Wealth Decile						
1 (Lowest)	0.172	[0.136,0.207]	0.219	[0.198,0.241]	0.047	0.024
2	0.118	[0.093,0.144]	0.169	[0.151,0.188]	0.051	0.0014
3	0.105	[0.078,0.132]	0.138	[0.120,0.155]	0.032	0.049
4	0.090	[0.068,0.111]	0.120	[0.105,0.135]	0.030	0.023
5	0.118	[0.093,0.143]	0.116	[0.100,0.132]	-0.002	0.89
6	0.077	[0.056,0.099]	0.097	[0.084,0.110]	0.020	0.13
7	0.073	[0.047,0.098]	0.092	[0.076,0.107]	0.019	0.21
8	0.093	[0.067,0.118]	0.103	[0.086,0.120]	0.010	0.52
9	0.080	[0.047,0.113]	0.101	[0.084,0.118]	0.021	0.27
10 (Highest)	0.060	[0.040,0.081]	0.074	[0.058,0.089]	0.013	0.30
Household Non-Housing Wealth Decile						
1 (Lowest)	0.147	[0.113,0.180]	0.215	[0.195,0.235]	0.068	0.00055
2	0.114	[0.089,0.140]	0.184	[0.165,0.203]	0.070	<0.0001
3	0.121	[0.095,0.148]	0.148	[0.129,0.168]	0.027	0.11
4	0.100	[0.076,0.125]	0.129	[0.112,0.147]	0.029	0.054
5	0.110	[0.084,0.135]	0.104	[0.088,0.119]	-0.006	0.70
6	0.093	[0.067,0.118]	0.099	[0.085,0.113]	0.006	0.66
7	0.098	[0.072,0.124]	0.085	[0.071,0.098]	-0.013	0.37
8	0.079	[0.052,0.106]	0.090	[0.075,0.105]	0.011	0.48
9	0.057	[0.035,0.078]	0.094	[0.077,0.110]	0.037	0.0076
10 (Highest)	0.065	[0.045,0.085]	0.083	[0.067,0.099]	0.018	0.15

Notes: This table presents the estimates as Table 1 for the full sample aged 70+, including non-whites. The US sample includes 5,330 non-Hispanic white participants and 1,835 non-white participants. The England sample includes 3,147 non-Hispanic white participants and 77 non-white participants. All estimates are age-gender standardized to the overall 2016 aged 70+ white population in England. The difference is calculated as the prevalence in the US minus prevalence in England.

Table A10: Difference in Prevalence of Dementia with Controls, Full Sample

Whole Sample:					
England Prevalence	0.099	0.088	0.088	0.084	0.085
	[0.091 to 0.108]	[0.079 to 0.098]	[0.076 to 0.1]	[0.068 to 0.1]	[0.068 to 0.103]
United States	0.124	0.107	0.107	0.105	0.123
	[0.118 to 0.129]	[0.1 to 0.114]	[0.097 to 0.117]	[0.093 to 0.118]	[0.109 to 0.137]
Percentage Point Difference	2.44	1.87	1.90	2.14	3.80
p-value	<0.0001	<0.0001	0.00015	0.00011	<0.0001
Excluding Lowest Income Decile:					
England Prevalence	0.096	0.086	0.086	0.087	0.090
	[0.087 to 0.105]	[0.076 to 0.096]	[0.073 to 0.099]	[0.071 to 0.103]	[0.072 to 0.108]
United States	0.113	0.098	0.099	0.100	0.117
	[0.107 to 0.118]	[0.091 to 0.106]	[0.089 to 0.108]	[0.086 to 0.113]	[0.103 to 0.132]
Percentage Point Difference	1.64	1.25	1.27	1.24	2.70
p-value	0.0024	0.020	0.019	0.030	<0.0001
Lowest Income Decile:					
England Prevalence	0.116	0.107	0.106	0.078	0.099
	[0.091 to 0.140]	[0.077 to 0.138]	[0.062 to 0.15]	[0.026 to 0.13]	[0.036 to 0.162]
United States	0.211	0.187	0.186	0.161	0.195
	[0.192 to 0.230]	[0.16 to 0.214]	[0.149 to 0.223]	[0.117 to 0.206]	[0.138 to 0.252]
Percentage Point Difference	9.54	7.93	7.96	8.37	9.60
p-value	<0.0001	<0.0001	<0.0001	<0.0001	<0.0001
Control for:					
Past Health Conditions		✓	✓	✓	✓
Ever Smoked			✓	✓	✓
Past BMI				✓	✓
Education					✓

Notes: Full sample, population aged 70+, including minorities. The sample sizes are 3,224 participants in England, and 7,165 participants in the United States. All estimates are age-gender standardized to the overall 2016 aged 70+ white population in England. The difference is calculated as the prevalence in the US minus prevalence in England. Differences are displayed as percentage points. 'Past Health Conditions' controls for whether an individual had any cardiometabolic diseases (diabetes, heart disease, and stroke) and/or psychiatric conditions 4 years prior. 'Ever Smoked' controls for whether an individual has ever smoked. BMI stands for Body Mass Index. 'Past BMI' includes dummy variables to control for whether an individual is classed as underweight, normal weight, *overweight* or *obese*. BMI measurements are based on when an individual first entered the survey, which is at least 10 years prior. We assume the effect of each control is constant across countries and age/gender groups. The reference groups are: No past health conditions; never smoked; normal weight; and high-school education.

Table A11: Prevalence of Dementia in United States, Whites versus Non-whites

	US Non-White and Hispanic		US Non-Hispanic White			
	Age-Gender Standardized Prevalence [95% CI]		Age-Gender Standardized Prevalence [95% CI]		Difference	p-value
All	0.182	[0.169 to 0.195]	0.112	[0.106 to 0.118]	-0.070	<0.0001
Household Income Decile						
1 (Lowest)	0.224	[0.199 to 0.249]	0.204	[0.178 to 0.231]	-0.020	0.28
2	0.211	[0.180 to 0.243]	0.143	[0.123 to 0.163]	-0.068	0.00034
3	0.220	[0.158 to 0.282]	0.121	[0.102 to 0.140]	-0.099	0.0023
4	0.202	[0.165 to 0.240]	0.133	[0.111 to 0.155]	-0.069	0.0018
5	0.116	[0.076 to 0.156]	0.094	[0.079 to 0.109]	-0.022	0.31
6	0.164	[0.097 to 0.230]	0.098	[0.081 to 0.116]	-0.065	0.065
7	0.129	[0.080 to 0.178]	0.107	[0.086 to 0.128]	-0.022	0.42
8	0.107	[0.047 to 0.166]	0.084	[0.067 to 0.101]	-0.023	0.47
9	0.072	[0.026 to 0.118]	0.090	[0.069 to 0.110]	0.017	0.50
10 (Highest)	0.084	[0.038 to 0.129]	0.077	[0.055 to 0.099]	-0.006	0.81
Years of Schooling						
9 or less	0.235	[0.212 to 0.258]	0.190	[0.162 to 0.218]	-0.045	0.015
10	0.172	[0.127 to 0.218]	0.137	[0.109 to 0.165]	-0.035	0.19
11	0.144	[0.109 to 0.179]	0.109	[0.080 to 0.139]	-0.035	0.14
12	0.198	[0.170 to 0.227]	0.124	[0.114 to 0.133]	-0.074	<0.0001
13	0.159	[0.101 to 0.218]	0.116	[0.090 to 0.141]	-0.043	0.18
14 or more	0.099	[0.079 to 0.119]	0.085	[0.076 to 0.093]	-0.015	0.20
Household Wealth Decile						
1 (Lowest)	0.228	[0.201 to 0.255]	0.208	[0.176 to 0.240]	-0.020	0.36
2	0.208	[0.179 to 0.237]	0.154	[0.131 to 0.177]	-0.054	0.0043
3	0.184	[0.142 to 0.227]	0.120	[0.102 to 0.138]	-0.064	0.0063
4	0.142	[0.111 to 0.174]	0.115	[0.098 to 0.132]	-0.027	0.13
5	0.159	[0.117 to 0.202]	0.108	[0.092 to 0.124]	-0.051	0.027
6	0.120	[0.084 to 0.155]	0.093	[0.080 to 0.107]	-0.026	0.18
7	0.156	[0.086 to 0.226]	0.088	[0.072 to 0.103]	-0.068	0.063
8	0.197	[0.134 to 0.259]	0.099	[0.081 to 0.116]	-0.098	0.0031
9	0.122	[0.065 to 0.178]	0.099	[0.082 to 0.117]	-0.022	0.46
10 (Highest)	0.045	[0.018 to 0.073]	0.075	[0.059 to 0.090]	0.029	0.071
Household Non-Housing Wealth Decile						
1 (Lowest)	0.231	[0.207 to 0.256]	0.197	[0.167 to 0.227]	-0.034	0.085
2	0.204	[0.177 to 0.231]	0.177	[0.151 to 0.203]	-0.027	0.16
3	0.187	[0.142 to 0.232]	0.135	[0.114 to 0.155]	-0.052	0.036
4	0.158	[0.128 to 0.188]	0.123	[0.104 to 0.143]	-0.034	0.059
5	0.106	[0.069 to 0.143]	0.102	[0.086 to 0.118]	-0.004	0.85
6	0.099	[0.063 to 0.134]	0.098	[0.084 to 0.113]	0.000	0.99
7	0.124	[0.066 to 0.182]	0.083	[0.069 to 0.097]	-0.040	0.18
8	0.140	[0.069 to 0.210]	0.088	[0.073 to 0.103]	-0.051	0.16
9	0.127	[0.054 to 0.201]	0.092	[0.075 to 0.108]	-0.035	0.36
10 (Highest)	0.069	[0.050 to 0.088]	0.084	[0.068 to 0.101]	0.016	0.23

Notes: The sample includes 5,330 non-Hispanic white participants and 1,835 non-white and Hispanic participants. All estimates are age-gender standardized to the overall 2016 aged 70+ white population in England. The difference is calculated as the non-white and Hispanic prevalence in the US minus the non-Hispanic white prevalence in the US.

Arapakis K, *et al. BMJ Open* 2022; 11:e045186. doi: 10.1136/bmjopen-2020-045186

Appendix References

1 Langa KM, Larson EB, Crimmins EM, *et al.* A comparison of the prevalence of dementia in the United States in 2000 and 2012. *JAMA Intern Med* 2017;**177**:51–8.

2 Hudomiet P, Hurd MD, Rohwedder S. The relationship between lifetime out-of-pocket medical expenditures, dementia, and socioeconomic status in the US. *J Econ Ageing* 2019;**14**:100181.

3 Rusmaully J, Dugravot A, Moatti J-P, *et al.* Contribution of cognitive performance and cognitive decline to associations between socioeconomic factors and dementia: A cohort study. *PLoS Med* 2017;**14**:e1002334.

4 Cadar D, Lassale C, Davies H, *et al.* Individual and area-based socioeconomic factors associated with dementia incidence in England: evidence from a 12-year follow-up in the English longitudinal study of ageing. *JAMA Psychiatry* 2018;**75**:723–32.

5 Rocca WA, Petersen RC, Knopman DS, *et al.* Trends in the incidence and prevalence of Alzheimer's disease, dementia, and cognitive impairment in the United States. *Alzheimers Dement* 2011;**7**:80–93.

6 Basu R. Education and dementia risk: results from the Aging Demographics and Memory Study. *Res Aging* 2013;**35**:7–31.

7 Nguyen TT, Tchetgen EJT, Kawachi I, *et al.* Instrumental variable approaches to identifying the causal effect of educational attainment on dementia risk. *Ann Epidemiol* 2016;**26**:71–6.

8 Crimmins EM, Kim JK, Langa KM, *et al.* Assessment of cognition using surveys and neuropsychological assessment: the Health and Retirement Study and the Aging, Demographics, and Memory Study. *J Gerontol B Psychol Sci Soc Sci* 2011;**66**:i162–71.

9 Garcia MA, Saenz J, Downer B, *et al.* The role of education in the association between race/ethnicity/nativity, cognitive impairment, and dementia among older adults in the United States. *Demogr Res* 2018;**38**:155.

10 Weden MM, Shih RA, Kabeto MU, *et al.* Secular trends in dementia and cognitive impairment of US rural and urban older adults. *Am J Prev Med* 2018;**54**:164–72.

11 Sonnega A, Faul JD, Ofstedal MB, *et al.* Cohort profile: the health and retirement study (HRS). *Int J Epidemiol* 2014;**43**:576–85.

12 Steptoe A, Breeze E, Banks J, *et al.* Cohort profile: the English longitudinal study of ageing. *Int J Epidemiol* 2013;**42**:1640–8.

13 Hurd MD, Martorell P, Delavande A, *et al.* Monetary costs of dementia in the United States. *N Engl J Med* 2013;**368**:1326–34.

14 Langa KM, Plassman BL, Wallace RB, *et al.* The Aging, Demographics, and Memory Study: study design and methods. *Neuroepidemiology* 2005;**25**:181–91.

15 Gianattasio KZ, Wu Q, Glymour MM, *et al.* Comparison of methods for algorithmic classification of dementia status in the health and retirement study. *Epidemiol Camb Mass* 2019;**30**:291.

16 Staff H. Sample sizes and response rates. *Ann Arbor MI Surv Res Cent Inst Soc Res Univ Mich Online Verfügbar Unter Httpshrs Isr Umich Edusitesdefaultf IlesbiblioResponseRates2017 Pdf Zugriff Am* 2017;**28**.

17 Banks J, Nazroo J, Steptoe A. Wave 6. *Inst Fisc Stud* 2014.
