# Supplementary material for: Dementia and disadvantage in the USA and England: population-based comparative study
Source: BMJ Open. 2021 Oct 5;11(10):e045186. doi: 10.1136/bmjopen-2020-045186 (PMC8496387; doi:10.1136/bmjopen-2020-045186)
Supplement: Author's manuscript [file bmjopen-2020-045186.draft_revisions.pdf]

BMJ Open

BMJ Open is committed to open peer review. As part of this commitment we make the peer review history of every article we publish publicly available.

When an article is published we post the peer reviewers' comments and the authors' responses online. We also post the versions of the paper that were used during peer review. These are the versions that the peer review comments apply to.

The versions of the paper that follow are the versions that were submitted during the peer review process. They are not the versions of record or the final published versions. They should not be cited or distributed as the published version of this manuscript.

BMJ Open is an open access journal and the full, final, typeset and author-corrected version of record of the manuscript is available on our site with no access controls, subscription charges or pay-per-view fees (http://bmjopen.bmj.com).

If you have any questions on BMJ Open's open peer review process please email info.bmjopen@bmj.com

Dementia and Disadvantage in the United States and England: International Cohort Study

Journal:	*BMJ Open*
Manuscript ID	bmjopen-2020-045186
Article Type:	Original research
Date Submitted by the Author:	24-Sep-2020

Complete List of Authors:	Arapakis, Karolos; UCL, Department of Economics<br>Brunner, Eric; University College London, Epidemiology and Public Health<br>French, Eric; UCL, Department of Economics; Institute for Fiscal Studies<br>McCauley, Jeremy; University of Bristol, School of Economics

Keywords:	Dementia < NEUROLOGY, PUBLIC HEALTH, HEALTH ECONOMICS

SCHOLARONE™
Manuscripts

I, the Submitting Author has the right to grant and does grant on behalf of all authors of the Work (as defined in the below author licence), an exclusive licence and/or a non-exclusive licence for contributions from authors who are: i) UK Crown employees; ii) where BMJ has agreed a CC-BY licence shall apply, and/or iii) in accordance with the terms applicable for US Federal Government officers or employees acting as part of their official duties; on a worldwide, perpetual, irrevocable, royalty-free basis to BMJ Publishing Group Ltd ("BMJ") its licensees and where the relevant Journal is co-owned by BMJ to the co-owners of the Journal, to publish the Work in this journal and any other BMJ products and to exploit all rights, as set out in our licence.

The Submitting Author accepts and understands that any supply made under these terms is made by BMJ to the Submitting Author unless you are acting as an employee on behalf of your employer or a postgraduate student of an affiliated institution which is paying any applicable article publishing charge ("APC") for Open Access articles. Where the Submitting Author wishes to make the Work available on an Open Access basis (and intends to pay the relevant APC), the terms of reuse of such Open Access shall be governed by a Creative Commons licence – details of these licences and which Creative Commons licence will apply to this Work are set out in our licence referred to above.

Other than as permitted in any relevant BMJ Author's Self Archiving Policies, I confirm this Work has not been accepted for publication elsewhere, is not being considered for publication elsewhere and does not duplicate material already published. I confirm all authors consent to publication of this Work and authorise the granting of this licence.

For peer review only

Dementia and Disadvantage in the United States and England: International Cohort Study

By Karolos Arapakis, Eric J Brunner, Eric French, Jeremy McCauley*

Objectives – To compare dementia prevalence and how it varies by socioeconomic status (SES) across the United States and England.

Design – International cohort study.

Setting - Non-Hispanic whites aged over 70 population in the United States and England.

Participants – Data was used from the Health and Retirement Study (HRS) and the English Longitudinal Study of Ageing (ELSA), which are harmonised, nationally representative panel studies. The sample includes 5,330 and 3,147 individuals in the United States and England, respectively.

Main outcome measures – Between country differences in age-gender standardized dementia prevalence, across the SES gradient. Dementia prevalence was estimated in each country using an algorithm based on an identical battery of demographic, cognitive, and functional measures.

Results – Both countries display large inequalities in dementia prevalence, with the United States being more unequal according to four measures of SES. Overall prevalence was lower in England at 9.7% (95% CI, 8.9% to 10.6%) than the US at 11.2% (95% CI, 10.6% to 11.8%), a difference of 1.4 percentage points (pp) (p = 0.0055). Most of the between country difference is driven by the bottom of the SES distribution. In the lowest income decile individuals in the US had 7.3 pp (p <0.0001) higher prevalence than in England. Once past health factors and education were controlled for, most of the within country inequalities disappeared, however the cross-country difference in prevalence for those in lowest income decile remained disproportionately high.

Conclusions – There is inequality in dementia prevalence according to income, wealth, and education in both the US and England. England has lower dementia prevalence and a less steep SES gradient. Most of the cross-country difference is concentrated in the lowest SES group, which provides evidence that disadvantage in the United States is a disproportionately high risk factor for dementia.

*Department of Economics, University College London, London, UK (K Arapakis MSc, Prof E French PhD)
Department of Epidemiology and Public Health, University College London, London, UK (Prof E J Brunner PhD)
Department of Economics, University of Bristol, Bristol, UK (J McCauley PhD)
Institute for Fiscal Studies, London, UK (E French)
Correspondence to: Jeremy McCauley, School of Economics, University of Bristol, Bristol BS8 1TU, UK **jeremy.mccauley@bristol.ac.uk**

For peer review only - http://bmjopen.bmj.com/site/about/guidelines.xhtml

Article summary

Strengths and limitations of this study

- First study to compare dementia prevalence across countries using the exact same measure of dementia.
- As ELSA and HRS share sample selection and questionnaire design, any differences in overall prevalence across the two countries should represent true differences.
- We measure the SES gradient of dementia across four different measures of SES: income; education; wealth; and non-housing wealth. All provide consistent results.
- We do not ascertain dementia directly, but predict caseness using a common battery of measures in ELSA and HRS.
- One of the SES measures, education, is also used as a predictor of dementia.

For peer review only

For peer review only - http://bmjopen.bmj.com/site/about/guidelines.xhtml

Introduction

Dementia, a severe and irreversible decline in memory and other cognitive functions, is a major and increasing global health challenge. It is the fifth leading cause of death globally and is one of the most common comorbidities for Covid-19 morbidity.[1,2] It results in large social and economic costs.[3,4]

Americans are more likely to be in poor health than their English counterparts in multiple dimensions, including heart disease and diabetes.[5] These differences are large along all points of the socioeconomic status (SES) gradient, although the gradient is generally steeper in the US. While the SES gradient for many diseases has been well established,[6–8] only a few studies have focused specifically on dementia.[9,10] The evidence of the SES gradient for dementia is also less clear, as in England while a strong association has been established between wealth and dementia incidence, the same was not observed for education level.[11]

The Global Burden of Disease Study (GBD) reported that in 2017 among those aged over 70, the US had a lower overall prevalence of dementia at 7.89% (7.15% to 8.59%) compared with the UK at 8.91% (7.80% to 10.09%).[12] However, the GBD has identified substantial heterogeneity in case-ascertainment methods throughout the dementia literature, resulting in location-specific inconsistencies and potentially biased cross-country comparisons. This has led to calls for analyses with more consistent and comparable measures of dementia to inform policy makers, researchers, and clinicians more accurately about global differences in dementia.[13]

In this study, we compared dementia prevalence in England and the US among non-Hispanic whites aged 70+, and how it varied across the SES gradient of each country. Location-specific inconsistencies caused by differences in diagnostic practices were not an issue in our study. We used two large surveys, the US Health and Retirement Study (HRS) and the English Longitudinal Study of Ageing (ELSA), that contain a battery of the same demographic, cognitive, and functional measures, and we used the same prediction algorithm in both countries to detect undiagnosed as well as diagnosed cases. We compared dementia prevalence within and across England and the US using important indicators of SES, specifically: income; education; wealth; and non-housing wealth.

Methods

Cohort Description

Data were extracted from the 2016 and earlier waves of the HRS and ELSA. Both are biennial surveys, representative of the aged over 50 population that collect a wide range of data on health, demographics, employment, and wealth. The design of ELSA was based on the HRS, making the two surveys highly similar. Both the HRS and ELSA follow respondents longitudinally until death, with new cohorts entering to maintain population representativeness as the study sample gets older.

Our samples are restricted to non-Hispanic whites over the age of 70 years old that live in the community or in nursing homes in 2016. This provides a study sample of 5,330 participants in the HRS and 3,147 participants in ELSA. We restrict our sample to non-Hispanic whites to ensure estimates are comparable across countries. In the online appendix we present estimates where we include ethnic minorities.

For peer review only - http://bmjopen.bmj.com/site/about/guidelines.xhtml

In addition to containing details on health conditions and any difficulties respondents have with Activities of Daily Living (ADLs) and Instrumental Activities of Daily Living (IADLs), both HRS and ELSA sample members have their cognitive function assessed. A range of tests adapted from the Telephone Interview for Cognitive Status (TICS) have been carried out in HRS since 1996 and ELSA since 2014. If a sample member was unable to respond in person, a proxy respondent was asked questions about the respondent's change in memory.

Patient and Public Involvement

No study participants were involved in setting the research question or outcome measures, nor were they involved in any other area of the design, implementation, and analysis of the study. There are no direct plans to disseminate the results of the research to study participants.

Dementia case definition

The HRS included a detailed clinical substudy (ADAMS: Aging, Demographics and Memory Study) of those aged 70+ with an in-depth in-home assessment of cognitive status by experienced teams at the Duke University Dementia Epidemiology Research Center who diagnosed each participant as normal, cognitively impaired but not demented (CIND), or demented.[14] Hurd et al. applied separate ordered probit models to self- and proxy-respondents to generate a predictive algorithm, based on the ADAMS diagnoses, for the whole HRS sample, using a range of variables including demographic information, Activities of Daily Living (ADLs), Instrumental Activities of Daily Living (IADLs), TICS questionnaire, and IQCODE, as well as the change in these variables across waves.[3]

We applied Hurd et al.'s predictive algorithm to estimate the probably of dementia for those in the HRS sample in 2016 and extended the prediction to the ELSA sample. The algorithm predicts the probability of dementia in the following year; therefore, we predicted dementia prevalence in 2017. Hurd et al's predictive algorithm has been shown to have a high accuracy of 93% (91% to 96%).[14] An in-depth discussion of the predictive algorithm procedure can be found in the online appendix.

Non-response for people unable or unwilling to participate in the survey is important when attempting to estimate dementia prevalence across the population. While attrition exists in both surveys, it is unlikely to significantly affect our estimates, as attrition in these surveys does not appear to be related to prior disease prevalence. Among older ELSA and HRS respondents there is no statistically significant correlation between attrition and the SES indicators of education, income and wealth.[15]

Measures of socioeconomic status

We considered four measures of SES: income; education; wealth; and non-housing wealth. Income is measured as current household income from all sources. For education we used total years of schooling. Wealth is measured as respondents' reported household wealth and household non-housing wealth from 4 years prior to minimize reverse causality, as medical expenses associated with dementia are high and may run down wealth.[4] For each measure, we created a SES gradient by ranking individuals based on

For peer review only - http://bmjopen.bmj.com/site/about/guidelines.xhtml

For peer review only

that measure. For income, wealth, and non-housing wealth, we assigned everyone to a decile in their respective country. For education, we ranked individuals according to their number of years of schooling.

Statistical analysis

A pooled dataset of the two surveys was created and observations were stratified based on country. In our statistical analysis we used the respondent's inverse probability of being sampled to adjust for non-response and the sampling design of the surveys. To make both within country estimates along the SES gradient and cross-country estimates directly comparable, estimates were age-gender standardized to the English population aged over 70 in 2016 using direct standardization. We estimated the unconditional prevalence of dementia in each country, their difference, and compared the prevalence along the four SES gradients. For each estimate presented we computed the corresponding 95% confidence interval (CI), and for any differences we computed the corresponding p-values. For each SES factor, as well as estimating the age-gender standardized prevalence along the gradient, we calculated the Relative Index of Inequality (RII) and Slope Index of Inequality (SII) using generalized linear models (log binomial regression) with logarithmic and identity link functions, respectively. The RII can be interpreted as the relative likelihood of dementia prevalence of those in the lowest SES group compared to those in the highest, and the SII can be interpreted as the absolute effect on dementia probability of moving from the lowest SES group to the highest.[16] To assess whether any observed differences could be explained by disparities in past health risk factors across countries we conditioned on a variety of risk factors and assessed how our estimates changed. Where possible, when conditioning on these factors we used past health instead of current health to address the problem of reverse causality: i.e., the problem that dementia may cause health problems such as low weight. Statistical analyses were performed using STATA software.

Results

Table 1 shows the age-gender standardized prevalence of dementia for the aged over 70 white non-Hispanic population in both England and the US. Dementia prevalence is lower in England at 9.7% (95% CI, 8.9% to 10.6%) than the US at 11.2% (95% CI, 10.6% to 11.8), a difference of 1.4 percentage points (pp) that is highly statistically significant (P = 0.0055).

Table 1 also shows dementia prevalence for different SES groups, in terms of income, education, wealth, and non-housing wealth. Regardless of the measure of the SES, there is a clear gradient in dementia prevalence, with the most disadvantaged groups in both England and the US having higher dementia prevalence. The gradient is steeper in the US and is driven by significantly higher dementia prevalence for those at the very bottom of the distribution. In the US, those in the lowest income decile have a dementia prevalence of 18.7% (95% CI, 16.6% to 20.8%), which is considerably higher than in England, with a prevalence among those in the lowest decile of 11.4% (95% CI, 8.9% to 13.9%). The difference is highly statistically significant (*P < 0.0001).* Table 1 shows that for income deciles above the lowest, the difference across the two countries is much smaller and not statistically significant. This same general pattern is

For peer review only - http://bmjopen.bmj.com/site/about/guidelines.xhtml

evident across the other measures of SES that we consider, although when using wealth, the difference between those in the bottom decile is not statistically significant.

Figure 1 presents the same dementia prevalence information shown in Table 1, but in graphical format. It also plots the Slope Index of Inequality (SII) for the four different measures of the SES for both countries. In both the US and England, dementia is more prevalent among the more disadvantaged. The gradient tends to be steeper in the US, corresponding to a larger SII in the US for each SES measure. For income, the SII is -0.062 (95% CI, -0.097 to -0.028) and -0.085 (95% CI, -0.114 to -0.057) for England and the US, respectively. The SIIs are statistically similar. If the lowest income decile is excluded, the SII for England becomes slightly steeper (-0.067 (95% CI, -0.107 to -0.027)) whereas the SII for the US becomes less steep than England (-0.060 (95% CI, 0.093 to -0.027)).

We extended the analysis to account for cardiometabolic diseases (i.e. diabetes, heart disease and stroke), and behaviours (i.e. smoking and body mass index) as dementia risk factors.[1,17] Previous research showed these factors to be more prevalent in the US than England, especially among the most disadvantaged.[5] Table 2 displays the percentage point difference in dementia prevalence after we controlled for various measures of past health. The results are split into three panels: the whole sample; the whole sample excluding those in the lowest income decile; and the lowest income decile. Controlling for past health-related factors only modestly reduced overall dementia prevalence in both England and the US. However, it had a relatively larger impact on those in the lowest income decile. In England, when we controlled for health-related factors, most of the SES gradient disappeared. In the US, when we controlled for health-related factors, while most of the SES gradient disappeared, prevalence in the lowest income decile remained disproportionately high.

As was shown in Table 1, the difference in the prevalence of dementia between England and the US is 1.43 pp ($P = 0.0055$). Table 2 shows this difference declined to 0.894 pp ($P = 0.11$) when we excluded the lowest income decile. Controlling for past health modestly reduced this cross-country difference further. After controlling for past health-related factors, the difference declined by a maximum of 19%. For the lowest income decile, controlling for past health-related factors, reduced the cross-country difference of 7.27 pp ($P < 0.0001$) by 33%.

Education has also been shown to be a risk factor for dementia. Table 1 shows that in both the US and England, the less educated have higher dementia prevalence. Controlling for education increased the estimated difference across countries, from 1.43 pp to 2.82 pp, as can be seen in the online appendix Table A5.

In our main analysis we exclude ethnic minorities, who have higher dementia prevalence and comprise a larger share of the US than the English population. Including minorities increased the estimated difference across countries, from 1.43 pp to 2.42 pp, as can be seen in the online appendix Tables A7 and A8.

Discussion

Main findings

Using nationally representative samples of older individuals from England and the US, and applying the same algorithmic procedure to predict dementia in both samples, we showed that in both the US and

For peer review only - http://bmjopen.bmj.com/site/about/guidelines.xhtml

England dementia is more prevalent among the disadvantaged, and the SES gradient of dementia is steeper in the US. The steeper gradient in the US is largely driven by those in the lowest decile, suggesting an excess burden of dementia and underlying causes of dementia among the poorest in the US population. In both countries, most of the SES gradient disappeared when we controlled for past health related factors, however prevalence for those in lowest income decile in the US remained disproportionately high. If the lowest income decile is excluded from our sample the difference in dementia prevalence across the countries is statistically insignificant, and any remaining SES gradient of dementia is remarkably similar across countries.

While poorer individuals face a higher burden of dementia in both England and the US, the extremely poor in the US face a disproportionately high burden of dementia. Controlling for past health-related factors can explain some, but not the all, of the cross-country difference. It can explain up to around one third of the difference for those in the lowest income decile. While past health factors such as adiposity and smoking are correlated with dementia, those in the lowest income decile in the US do not smoke more or have higher BMIs than their English counterparts. Therefore, this cannot explain their disproportionately higher prevalence of dementia. Education also cannot explain the difference, as the US population is more educated at every income decile, and in fact the educational difference masks some of the underlying dementia risk difference between countries.

Adding minorities increased estimated dementia prevalence, especially in the US, because dementia prevalence is higher among minorities, who comprise a higher share of the US than the English population. This fits with prior research which showed dementia prevalence is higher for non-whites and Hispanics.[17,18] We did not observe higher dementia prevalence among Hispanics and non-whites in the US for those in relatively high socioeconomic groups.

Comparison with previous studies and how findings are an advance on current literature

Previous studies have shown cross-country variation in dementia prevalence,[1] however substantial heterogeneity in case-ascertainment methods across countries and studies makes interpreting any observed differences difficult. We believe this is the first study to compare dementia prevalence in England and the US using the exact same measure of dementia. Some studies have shown in both England and the US those with lower education and less wealth have been found to have higher rates of dementia,[4,9,11,19,20] but there are no systematic comparative studies. We compared prevalence along the SES gradient using almost identical measures of income, wealth, and education. Further, we standardized the cross-country comparison for age and gender, using England as the reference population. We found that dementia prevalence is higher and more concentrated among the poorest in the US than England. Detailed disaggregation according to SES measures shows the true extent of the excess burden of dementia in the very poorest group in the US.

We showed that risk factors for dementia such as cardiometabolic diseases, psychiatric conditions, high BMI, smoking has similar affects across countries. Accounting for these risk factors removes most of the SES gradient for both countries, but disproportionately high prevalence remains for the most disadvantaged in the US.

For peer review only - http://bmjopen.bmj.com/site/about/guidelines.xhtml

Implications (wider interpretation)

Much research has shown that low income Americans are more likely to be in poor health and die younger than their high-income counterparts.[21] We show that these health differences also extend to dementia prevalence.

While risk factors contribute to higher prevalence among those who are more SES disadvantaged, those in the US appear to have an undue burden that is caused by risk factors for which we cannot account. The NHS provides broadly equitable care according to education in the older population after accounting for health status.[22] In the US, virtually every American aged 65 or older is eligible for Medicare, however even then around 20% of individuals' healthcare must be financed out of pocket.[23] The extent to which health care provision below and above aged 65 may account for the relative excess dementia burden in the US is unclear.

The implications of our results are that interventions designed to attempt to prevent dementia should be targeted towards the most disadvantaged. This is especially true in the US. As yet, we are unable to advocate specific measures as we do not yet understand the specific nature of disadvantage in respect to dementia risk.

Strengths and Weaknesses of Analysis

This study has a strong design. Results are directly comparable across England and the US. The same predictive algorithm was applied to both countries, which excluded the problem of heterogeneity in case ascertainment which has affected the literature.[1,13] Further, because ELSA and HRS share sample selection and questionnaire design, any differences in overall prevalence and SES gradients in prevalence across the two countries should represent true differences. In contrast to the Global Burden Disease study, we find higher dementia prevalence in the US. Furthermore, we measured the SES gradient of dementia across four different measures of SES, with consistent results. Our work also highlights the usefulness of the standardized measure of dementia to allow for meaningful comparisons across countries.

This study had two limitations. First, we do not ascertain dementia directly, but predict caseness using a common battery of measures in ELSA and HRS. Importantly, Hurd's prediction algorithm has high accuracy and although our case definition lacks a clinical point of reference in England, it is based on a detailed clinical substudy in the US.[14] Further, cross-cultural subjectivities in reporting of impairment severity are likely to be similar in the US and England (online appendix Table A2). It would be of great value for future work to use the Harmonized Cognitive Assessment Protocol (HCAP) data to provide a standard clinical point of reference to validate and verify the cross-country dementia prevalence estimates.[24] Second, education is one of the factors in the predictive algorithm for dementia and also one of our measures of SES. The dementia algorithm takes account of the well-documented correlation between education level and cognitive function in adult life. Nevertheless, we found substantial absolute and relative inequalities in dementia prevalence according to education level in the US and UK. Analysis of HRS data using schools policy and genetic variants as instrumental variables suggests education may be a successful approach for reducing dementia risk.[25]

For peer review only - http://bmjopen.bmj.com/site/about/guidelines.xhtml

For peer review only

Conclusion

Given the large social and economic costs of dementia, there is great value in understanding the scope and burden of dementia in the population along the SES gradient. This study indicates that more disadvantaged individuals face a higher burden of dementia and that the poorest individuals in the US face a disproportionately high burden. The high burden faced by these individuals can be partly but not fully be explained by past health factors. We lacked data on other important possible contributing factors such as habitual drug use. Further research is needed to fully understand this issue using data from multiple sources.

For peer review only

For peer review only - http://bmjopen.bmj.com/site/about/guidelines.xhtml

Table 1: Prevalence of Dementia, United States vs. England, 2017. Overall prevalence and prevalence according to four measures of socioeconomic status

	England		United States		Difference	p-value
	Age-Gender Standardized Prevalence [95% CI]		Age-Gender Standardized Prevalence [95% CI]			
All	0.097	[0.089 to 0.106]	0.112	[0.106 to 0.118]	0.014	0.0055
Household Income Decile						
1 (Lowest)	0.114	[0.089 to 0.139]	0.187	[0.166 to 0.208]	0.073	<0.0001
2	0.113	[0.090 to 0.136]	0.141	[0.119 to 0.163]	0.028	0.090
3	0.124	[0.097 to 0.151]	0.111	[0.095 to 0.127]	-0.013	0.42
4	0.099	[0.071 to 0.126]	0.118	[0.099 to 0.137]	0.019	0.26
5	0.094	[0.072 to 0.116]	0.086	[0.069 to 0.102]	-0.008	0.56
6	0.098	[0.070 to 0.127]	0.108	[0.088 to 0.128]	0.010	0.59
7	0.068	[0.042 to 0.093]	0.100	[0.078 to 0.122]	0.032	0.060
8	0.083	[0.053 to 0.114]	0.082	[0.066 to 0.097]	-0.002	0.92
9	0.082	[0.041 to 0.122]	0.093	[0.071 to 0.116]	0.012	0.62
10 (Highest)	0.059	[0.035 to 0.083]	0.077	[0.052 to 0.103]	0.019	0.30
Years of Schooling						
9 or fewer	0.128	[0.101 to 0.154]	0.190	[0.162 to 0.218]	0.062	0.0015
10	0.095	[0.074 to 0.116]	0.137	[0.109 to 0.165]	0.042	0.018
11	0.096	[0.077 to 0.115]	0.109	[0.080 to 0.139]	0.013	0.471
12	0.071	[0.042 to 0.100]	0.124	[0.114 to 0.133]	0.053	0.00060
13	0.061	[0.038 to 0.083]	0.116	[0.090 to 0.141]	0.055	0.0013
14 or more	0.056	[0.039 to 0.073]	0.085	[0.076 to 0.093]	0.029	0.0031
Household Wealth Decile						
1 (Lowest)	0.165	[0.132 to 0.198]	0.187	[0.162 to 0.211]	0.022	0.31
2	0.117	[0.092 to 0.143]	0.149	[0.129 to 0.169]	0.031	0.061
3	0.100	[0.073 to 0.127]	0.107	[0.091 to 0.122]	0.006	0.68
4	0.093	[0.071 to 0.115]	0.115	[0.098 to 0.132]	0.022	0.12
5	0.110	[0.085 to 0.134]	0.091	[0.077 to 0.106]	-0.018	0.21
6	0.080	[0.057 to 0.103]	0.089	[0.074 to 0.103]	0.008	0.55
7	0.070	[0.046 to 0.094]	0.103	[0.084 to 0.123]	0.034	0.034
8	0.092	[0.066 to 0.118]	0.089	[0.072 to 0.106]	-0.003	0.85
9	0.082	[0.048 to 0.116]	0.103	[0.084 to 0.123]	0.021	0.28
10 (Highest)	0.060	[0.038 to 0.081]	0.067	[0.051 to 0.084]	0.008	0.58
Household Non-Housing Wealth Decile						
1 (Lowest)	0.136	[0.103 to 0.168]	0.201	[0.176 to 0.226]	0.065	0.0019
2	0.109	[0.084 to 0.134]	0.137	[0.119 to 0.156]	0.029	0.074
3	0.123	[0.095 to 0.151]	0.131	[0.111 to 0.150]	0.008	0.66
4	0.096	[0.073 to 0.118]	0.101	[0.085 to 0.116]	0.005	0.71
5	0.108	[0.082 to 0.134]	0.107	[0.091 to 0.123]	-0.001	0.95
6	0.093	[0.067 to 0.119]	0.086	[0.070 to 0.101]	-0.007	0.64
7	0.099	[0.072 to 0.125]	0.086	[0.070 to 0.102]	-0.013	0.42
8	0.078	[0.051 to 0.105]	0.090	[0.073 to 0.106]	0.011	0.49
9	0.058	[0.036 to 0.081]	0.092	[0.074 to 0.111]	0.034	0.024
10 (Highest)	0.063	[0.043 to 0.084]	0.079	[0.062 to 0.095]	0.015	0.26

Notes: Sample includes non-Hispanic white population aged 70+ only. The sample size is 3,147 participants in England and 5,330 participants in the United States. All estimates are age-gender standardized to the overall 2016 aged 70+ white population in England. The difference is calculated as the prevalence in the US minus prevalence in England. All deciles are calculated within country.

For peer review only - http://bmjopen.bmj.com/site/about/guidelines.xhtml

Figure 1: SES Gradient of Dementia, United States vs England, 2017, according to four measures of socioeconomic status. Absolute and relative inequality shown with 95% confidence intervals.

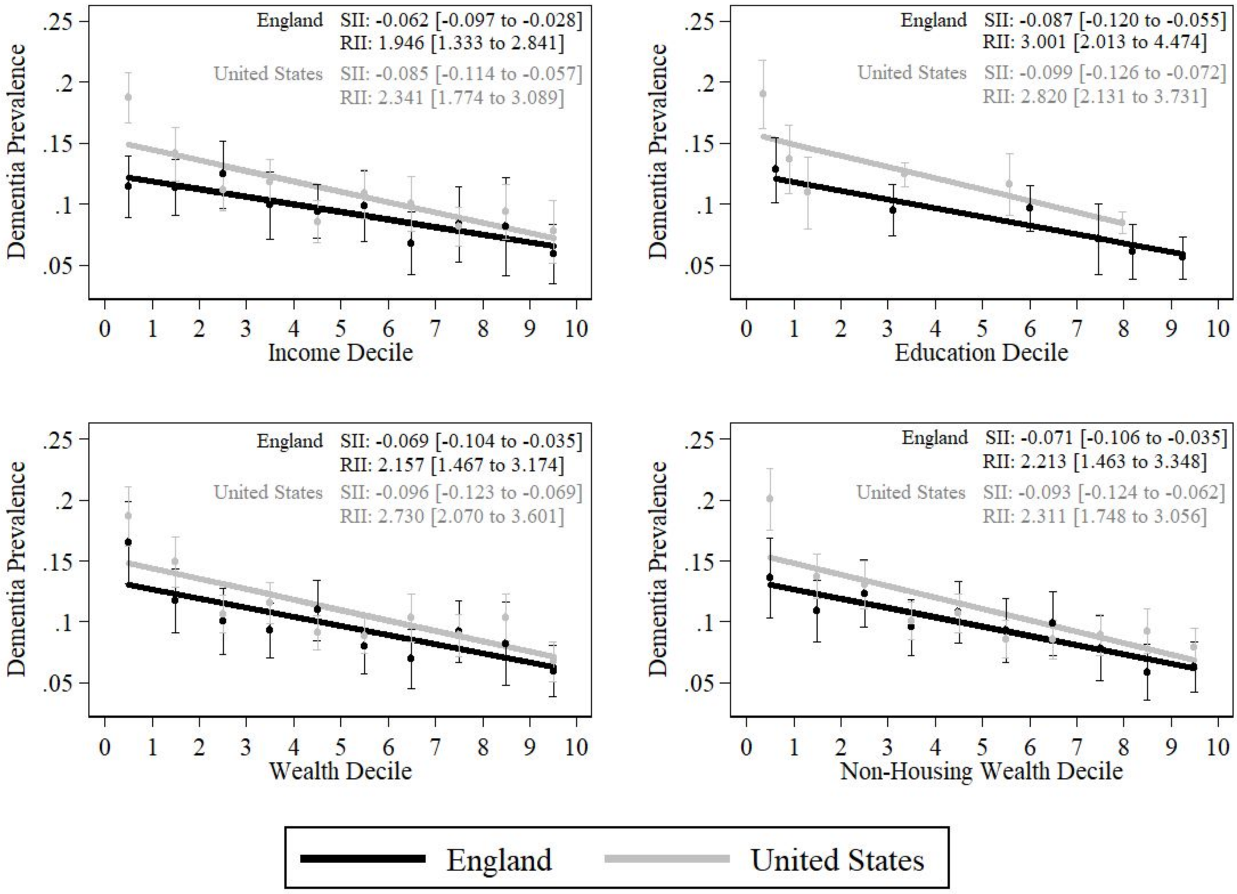

Notes: The points in this figure represent the mean age-gender standardized dementia prevalence for each country by socioeconomic status (SES), along with 95% confidence interval for these predictions. The solid lines represent the fitted Slope Index of Inequality (SII: absolute inequality) for each country. The values of the SII and the corresponding Relative Index of Inequality (RII: relative inequality) are listed in the top right of each figure, with 95% Confidence Intervals in brackets. For education, individuals are ranked based on their years of schooling within each country, and as the US has higher educational attainment, individuals with 14+ years of schooling are the 80th percentile of the US education distribution, but at the 90th percentile of the English educational distribution.

For peer review only - http://bmjopen.bmj.com/site/about/guidelines.xhtml

Table 2: Difference in Prevalence of Dementia, United States vs England, 2017. Estimates of the between-country difference taking account of antecedent health status, smoking and degree of adiposity

Whole Sample:					
Percentage Point Difference	1.43	1.34	1.15	1.21	1.18
p-value	0.0055	0.0091	0.025	0.020	0.034
% Difference from Baseline	--	-6%	-19%	-16%	-17%
Excluding Lowest Income Decile:					
Percentage Point Difference	0.89	0.88	0.74	0.75	0.81
p-value	0.11	0.11	0.18	0.18	0.18
% Difference from Baseline	--	-2%	-17%	-16%	-10%
Lowest Income Decile:					
Percentage Point Difference	7.27	6.14	5.45	5.93	4.85
p-value	<0.0001	0.00032	0.0020	0.0011	0.012
% Difference from Baseline	--	-15%	-25%	-18%	-33%
Control for:					
Past Cardiometabolic Diseases		✓	✓	✓	✓
Past Psychiatric Conditions			✓	✓	✓
Ever Smoked				✓	✓
Past BMI					✓

Notes: Sample includes non-Hispanic white population aged 70+ only. The sample size is 3,147 participants in England and 5,330 participants in the United States. All estimates are age-gender standardized to the overall 2016 aged 70+ white population in England. The difference is calculated as the prevalence in the US minus prevalence in England. Differences are displayed as percentage points. 'Past Cardiometabolic Diseases' and 'Past Psychiatric Conditions' control for whether an individual says they had the conditions 4 years prior. 'Cardiometabolic Diseases' include diabetes, heart disease, and stroke. 'Smoking' controls for whether an individual has ever smoked. BMI stands for Body Mass Index. 'Past BMI' includes dummy variables to control for whether an individual is classed as underweight, overweight, or obese. BMI values are based on when an individual first entered the survey, which is at least 10 years prior.

For peer review only

Contributors and Transparency Statement

All authors made a substantial contribution to study conception and design. EF and JM developed the original idea. JM and KA prepared and analysed the data, with inputs from EF and EJB. All authors contributed to interpreting the results and drafting the manuscript. JM and KA serve as guarantors and affirm that the manuscript is an honest, accurate, and transparent account of the study being reported; that no important aspects of the study have been omitted; and that any discrepancies from the study as planned have been explained. All authors had full access to all of the data (including statistical reports and tables) in the study and take responsibility for the integrity of the data and accuracy of the data analysis. The corresponding author (JM) attests that all listed authors meet authorship criteria and that no others meeting the criteria have been omitted. Author names on manuscript are listed in alphabetical order.

Competing interests

All authors have completed the ICMJE uniform disclosure form and declare no support from any organisation for the submitted work; no financial relationships with any organisations that might have an interest in the submitted work in the previous three years; no other relationships or activities that could appear to have influenced the submitted work

Acknowledgments

We thank Paco Martorell and Ken Langa for help with understanding the prediction equations and Liliana French for editorial assistance.

Ethical approval

The HRS has ethical approval from the University of Michigan Institutional Review Board. ELSA has ethical approval from the London Multicentre Research Ethics Committee (MREC/01/2/91). This study did not require additional ethical approval as we conduct secondary analysis of these two publicly available datasets.

Funding

This work was supported by grants from the British Heart Foundation (RG/13/2/30098, RG/16/11/32334), MRRC, ESRC, and Onassis Foundation.

Data sharing

Appendix with details on the formula and calculations is available online. Code and results are available on request from the corresponding author.

For peer review only

References

1 Nichols E, Szoeke CE, Vollset SE, *et al.* Global, regional, and national burden of Alzheimer's disease and other dementias, 1990–2016: a systematic analysis for the Global Burden of Disease Study 2016. *Lancet Neurol* 2019;**18**:88–106.

2 Kuo C-L, Pilling LC, Atkins JL, *et al.* ApoE e4e4 genotype and mortality with COVID-19 in UK Biobank. *medRxiv* 2020.

3 Hurd MD, Martorell P, Delavande A, *et al.* Monetary costs of dementia in the United States. *N Engl J Med* 2013;**368**:1326–1334.

4 Hudomiet P, Hurd MD, Rohwedder S. The relationship between lifetime out-of-pocket medical expenditures, dementia, and socioeconomic status in the US. *J Econ Ageing* 2019;**14**:100181.

5 Banks J, Marmot M, Oldfield Z, *et al.* Disease and disadvantage in the United States and in England. *Jama* 2006;**295**:2037–2045.

6 Marmot MG. Status syndrome: a challenge to medicine. *Jama* 2006;**295**:1304–1307.

7 Brunner EJ. Social factors and cardiovascular morbidity. *Neurosci Biobehav Rev* 2017;**74**:260–268.

8 Marmot M. Health equity in England: the Marmot review 10 years on. *Bmj* 2020;**368**.

9 Rusmaully J, Dugravot A, Moatti J-P, *et al.* Contribution of cognitive performance and cognitive decline to associations between socioeconomic factors and dementia: A cohort study. *PLoS Med* 2017;**14**:e1002334.

10 George KM, Lutsey PL, Kucharska-Newton A, *et al.* Life Course Individual and Neighborhood Socioeconomic Status and Risk of Dementia in the Atherosclerosis Risk in Communities Neurocognitive Study (ARIC-NCS). *Am J Epidemiol* 2020.

11 Cadar D, Lassale C, Davies H, *et al.* Individual and area-based socioeconomic factors associated with dementia incidence in England: evidence from a 12-year follow-up in the English longitudinal study of ageing. *JAMA Psychiatry* 2018;**75**:723–732.

12 Global Burden of Disease. Global Burden of Disease. GBD Compare. IHME Viz Hub. GBD Comp. Viz Hub. https://vizhub.healthdata.org/gbd-compare/ (accessed 18 Jul 2020).

13 Launer LJ. Statistics on the burden of dementia: need for stronger data. *Lancet Neurol* 2019;**18**:25–27.

14 Gianattasio KZ, Wu Q, Glymour MM, *et al.* Comparison of methods for algorithmic classification of dementia status in the health and retirement study. *Epidemiol Camb Mass* 2019;**30**:291.

15 Banks J, Muriel A, Smith JP. Attrition and health in ageing studies: evidence from ELSA and HRS. *Longitud Life Course Stud* 2011;**2**:101–26.

16 Wagstaff A, Paci P, Van Doorslaer E. On the measurement of inequalities in health. *Soc Sci Med* 1991;**33**:545–557.

17 Yaffe K, Falvey C, Harris TB, *et al.* Effect of socioeconomic disparities on incidence of dementia among biracial older adults: prospective study. *Bmj* 2013;**347**.

18 Vega IE, Cabrera LY, Wygant CM, *et al.* Alzheimer's disease in the latino community: Intersection of genetics and social determinants of health. *J Alzheimers Dis* 2017;**58**:979–992.

For peer review only - http://bmjopen.bmj.com/site/about/guidelines.xhtml

19 Langa KM, Larson EB, Crimmins EM, *et al.* A comparison of the prevalence of dementia in the United States in 2000 and 2012. *JAMA Intern Med* 2017;**177**:51–58.

20 Rocca WA, Petersen RC, Knopman DS, *et al.* Trends in the incidence and prevalence of Alzheimer's disease, dementia, and cognitive impairment in the United States. *Alzheimers Dement* 2011;**7**:80–93.

21 Chetty R, Stepner M, Abraham S, *et al.* The association between income and life expectancy in the United States, 2001-2014. *Jama* 2016;**315**:1750–1766.

22 Stoye G, Zaranko B, Shipley M, *et al.* Educational inequalities in hospital use among the over 65s in England. *Milbank Q* 2020.

23 De Nardi M, French E, Jones JB, *et al.* Medical spending of the US elderly. *Fisc Stud* 2016;**37**:717–747.

24 Langa KM, Ryan LH, McCammon RJ, *et al.* The health and retirement study harmonized cognitive assessment protocol project: study design and methods. *Neuroepidemiology* 2020;**54**:64–74.

25 Nguyen TT, Tchetgen EJT, Kawachi I, *et al.* Instrumental variable approaches to identifying the causal effect of educational attainment on dementia risk. *Ann Epidemiol* 2016;**26**:71–76.

For peer review only

For peer review only - http://bmjopen.bmj.com/site/about/guidelines.xhtml

Author Licence

I *Jeremy McCauley [*The Corresponding Author] of this article contained within the original manuscript which includes any diagrams & photographs within and any related or stand alone film submitted (the Contribution") has the right to grant on behalf of all authors and does grant on behalf of all authors, a licence to the BMJ Publishing Group Ltd and its licencees, to permit this Contribution (if accepted) to be published in the BMJ and any other BMJ Group products and to exploit all subsidiary rights, as set out in our licence set out at: http://www.bmj.com/about-bmj/resources-authors/forms-policies-and-checklists/copyright-open-access-and-permission-reuse."

IF YOU ARE A NATIONAL INSTITUTE OF HEALTH ("NIH") EMPLOYEE, CONTRACTOR OR TRAINEE the following cover sheet will be accepted by the BMJ Group and NIH and incorporated into the above Licence.

Please tick **one or more** boxes as appropriate:

- ☐ I am the sole author of the Contribution.
- ✓ I am one author signing on behalf of all co-owners of the Contribution.
- ☐ The Contribution has been made in the course of my employment and I am signing as authorised by my employer.
- ☐ I am a US Federal Government employee acting in the course of my employment.
- ☐ I am not a US Federal Government employee, but some or all of my co-authors are.
- ☐ I am an employee of the UK Crown* acting in the course of my employment
- ☐ I am a US Federal Government employee acting in the course of my employment.
- ☐ I am not a US Federal Government employee, but some or all of my co-authors are.
- ☐ I am an employee of the UK Crown acting in the course of my employment
- ☐ I am not an employee of the UK Crown acting in the course of my employment but some/all of my co-authors are.*

For peer review only - http://bmjopen.bmj.com/site/about/guidelines.xhtml

Appendix (For Online Publication)

Research in context

We reviewed existing evidence in July 2020, searching PubMed database for any studies looking at cross-country comparisons of dementia using the same standard measures and diagnostic practices.

("Dementia"[mesh]) AND ("Prevalence"[mesh])

AND

(“Survey”[tw]) AND (“Population”[tw]) AND (“Representative”[tw])

AND

(“Cross-Country”[ti] OR “Comparison”[ti])

This search yielded no results. The only relevant project we could find relating to our research was regarding the Health and Retirement Study Harmonized Cognitive Assessment Protocol (HCAP) Project. [1] This is an international research collaboration funded by the National Institute on Aging to better measure and identify cognitive impairment and dementia in longitudinal studies of countries around the world. However, this project has not yet released any international comparisons.

We also reviewed existing evidence in the PubMed database for any studies looking at how dementia prevalence varies by socioeconomic status (SES) in either England or the US. The search terms used were the following:

("Dementia"[mesh]) AND

("Prevalence"[tw]) AND ("Socioeconomic"[ti] OR "Socioeconomic"[tw] OR "SES"[tw]) AND

(“United Kingdom”[mesh] OR “United Kingdom”[tw] OR “England”[tw] OR “UK”[tw] OR “United Kingdom”[tw] "Britain"[tw] OR “United States”[mesh] OR “United States”[tw] OR “US”[tw] OR “America”[tw])

This search yielded 50 results. Papers which were not relevant were manually removed. We also excluded papers that did not use nationally representative samples. We performed additional searches using lists of references retrieved from relevant papers. Relevant papers found in the search can be found in Table A1 below.

For peer review only

For peer review only - http://bmjopen.bmj.com/site/about/guidelines.xhtml

Table A1: Studies on Dementia Prevalence by Socioeconomic Status (SES)

Authors	Country	Socioeconomic Indicator	Years	Findings
Langa et al. (2017)[1]	United States	Education	2000 and 2012	Link between education and dementia risk
Hudomiet et al, (2019)[2]	United States	Education and Social Security Benefits	1998 to 2014	Link between education and dementia risk
Rusmaully et al, (2017)[3]	United Kingdom	Education, height, occupational position	Various years between 1985 and 2015	High cognitive reserve associated with lower risk for dementia
Cadar et al, (2018)[4]	United Kingdom	Education, wealth and the index of multiple deprivation	2002 to 2015	Lower wealth in but not education associated with increased risk for dementia
Rocca et al,(2011)[5]	United States	Education, net worth	1993 and 2002	Higher education, higher net worth protected against cognitive impairment.
Basu (2013)[6]	United States	Education	2000 - 2002	Education has causal effect on dementia risk
Nguyen et al, (2016)[7]	United States	Education	1998 to 2010	Education protective against dementia risk
Crimmins et al, (2018)[8]	United States	Education	2000 and 2010	More education linked to lower dementia prevalence
Garcia et al, (2018)[9]	United States	Race, ethnicity, nativity, and education	2012	Education reduces the odds for CIND
Weden et al, (2018)[10]	United States	Race, ethnicity, total number of children, marital status, highest educational attainment, and net total assets in 2000	2000 and 2010	Strong protective role of educational attainment and persisting rural disadvantages for dementia

For peer review only

For peer review only - http://bmjopen.bmj.com/site/about/guidelines.xhtml

Data Appendix

HRS Data

The Health and Retirement Study (HRS) is a nationally representative, biennial longitudinal survey of adults in the United States. It started in 1992 and since it collects a wide range of questions on income, wealth, employment, health, cognition, and demographics. It utilises a steady-state sampling design, with a new cohort aged 51-56 entering every 6 years. In total, 43,478 individuals have been interviewed to date. We used data from the 2016 and earlier waves to predict dementia and focused on the over the age of 70 years old that live in the community or in nursing homes in 2016. This left a sample of 7,165 individuals. We restricted our attention to non-Hispanic whites for comparability with ELSA. These restrictions generated a main study sample of 5,330 participants with 4,932 being self-respondents, and 398 proxy interviews.

ELSA Data

The English Longitudinal Study of Ageing (ELSA) is a biennial longitudinal survey of adults in England, developed as a companion study to the HRS. ELSA was also designed to be nationally representative of the non-institutionalised population. Respondents remain the study if they become institutionalised. While it is has been shown to be representative of the English population in terms of sociodemographic characteristics, the proportion of non-white people in the survey is very small.[11] We used data from the 2016 and earlier waves and focused on the aged over 70 years old that live in the community or in nursing homes in 2016. This left a sample of 3,224 individuals. We restricted our attention to non-Hispanic whites for comparability. These restrictions generated a study sample of 3,147 participants with 3,007 being self-respondents, and 140 proxy interviews.

Harmonised Variables HRS and ELSA

We use the same set of cognitive and demographic variables available in both the HRS and ELSA.

For cognitive and demographic measures, we used the same questions as Hurd et al.[12] Specifically, we used variables on demographics, difficulties respondents have with Activities of Daily Living (ADLs), Instrumental Activities of Daily Living (IADLs), and a range of cognitive function tests adapted from the Telephone Interview for Cognitive Status (TICS) and their change across waves. For Proxy respondents we also used the shortened 16 question form of IQCODE.

We used four variables to measure socioeconomic status (SES). These are household income, education, total household wealth, and total household non-housing wealth. For the HRS we used variables from HRS RAND dataset. To establish each respondent's SES in terms of income, wealth, and non-housing wealth, we assigned everyone to a decile rank for each variable in their respective country. For education, we ranked respondents according to their number of years of schooling. We used respondents' reported wealth and non-housing wealth from 4 years prior. If a respondent had a missing value of income, wealth, or non-housing wealth we used the most recent non-missing observation from previous waves. All amounts of income, wealth and non-housing wealth were deflated to 2016 GBPs, and USD amounts were converted to GBPs using 1 USD = 0.75 GBP.

Finally, in our analysis, we investigated how variables that have been associated with dementia affect our results. These include Body Mass Index (BMI), past smoking behaviour, past stroke. Respondents are asked whether they have any health conditions, of which we use whether they had any cardiometabolic diseases (diabetes, heart disease, and stroke) and/or psychiatric conditions 4 years prior. BMI is collected by a nurse for every ELSA respondent when they first enter the survey, which for everyone over 70 in the 2016 survey means their BMI was collected over 10 years prior. BMI is recorded in each wave of HRS based on self-reported height and weight. To make the BMI measures in HRS comparable with ELSA, we used respondents first ever recorded BMI in HRS, which for our sample was at least 10 years prior.

Hurd et al. Dementia Prediction Algorithm

To estimate the probability of dementia for each individual present in our pooled data we follow the method of Hurd et al. that is based on the HRS supplement Aging, Demographics and Memory Study (ADAMS).[12] It is a representative subsample of HRS members aged over 70 that received a detailed in-home assessment of their cognitive status by experienced teams at the Duke University Dementia Epidemiology

For peer review only - http://bmjopen.bmj.com/site/about/guidelines.xhtml

Research Center.[13] Consensus conferences were used to establish a final diagnosis of dementia or a cognitive impairment with no dementia (CIND) for each participant. Hurd et al. applied separate ordered probit models to self- and proxy-respondents to generate a predictive algorithm, based on the ADAMS diagnoses, for the whole HRS sample, using a range of variables including demographic information, ADLs, IADLs, the TICS questionnaire, IQCODE, lagged variables and differences in variables between waves. The algorithm predicts the probability of dementia in the following year.

Most of the coefficients used in the predictive algorithm can be found in the online appendix of the Hurd et al. paper. To account for the small number of missing variables in HRS and ELSA we either used coefficients provided by the authors or estimated them by a conditional minimum distance estimator using the publicly available HRS data and the predicted values of the researcher contribution Dementia Predicted Probability Files (DPPF), that follows the method of Hurd et al. To verify our predictions in HRS, we compared our predictions with the DPPF data available on the HRS website for the year 2008 and found that we accurately matched predicted dementia.

Hurd et al's predictive algorithm has been shown to have high specificity and accuracy, and generally performs well in terms of sensitivity compared to other predictive algorithms for dementia.[14]

Table A2: Summary Statistics for Predictor Variables, England vs United States

	Full Sample		Lowest Income Decile	
	Age-Gender- Standardized Mean		Age-Gender- Standardized Mean	
	England	United States	England	United States
**Cognitive Scores**				
Dates (Out of 4)	3.663	3.387	3.573	3.198
Backward counting 20	0.912	0.875	0.935	0.830
Serial 7 (Out of 5)	3.787	3.364	3.667	2.580
Scissor	0.978	0.920	0.993	0.912
Cactus	0.915	0.891	0.913	0.842
PM/Vice-President	0.846	0.900	0.828	0.854
Immediate recall (Out of 10)	5.318	4.631	5.010	3.972
Delayed recall (Out of 10)	3.797	3.684	3.185	2.894
**Limitations**				
ADLs (Out of 6)	0.561	0.523	0.559	0.827
IADLs (Out of 5)	0.411	0.457	0.421	0.679
Proxy Respondent	0.050	0.068	0.038	0.075

Notes: Sample includes non-Hispanic white population aged 70+ only. The sample size is 3,147 participants in England and 5,330 participants in the United States. The Cognitive Scores are from a range of tests adapted from Telephone Interview for Cognitive Status (TICS). Only non-proxy respondents answered the TICS questions. ADL and IADLs are Activities of Daily Living and Instrumental Activities of Daily Living, respectively.

For peer review only

Table A3: Summary Statistics, England vs United States

	Full Sample		Lowest Income Decile	
	Age-Gender Standardized Mean		Age-Gender Standardized Mean	
	England	United States	England	United States
Age	79.5	79.3	79.7	79.7
Female	0.553	0.553	0.553	0.553
Married	0.584	0.547	0.148	0.170
Current Income and Wealth (£)				
Income	20,547	31,927	8,247	8,645
Wealth	271,511	236,431	120,261	46,762
Non-Housing Wealth	38,715	100,996	7,778	5,509
Wealth 4 Years Prior (£)				
Wealth	257,459	242,655	111,830	55,953
Non-Housing Wealth	40,604	110,530	6,476	13,724
Education				
Less than High School	0.424	0.160	0.619	0.323
High-school or Some College	0.444	0.559	0.318	0.556
College	0.132	0.281	0.063	0.122
Current Health				
ADLs (Out of 6)	0.561	0.523	0.559	0.827
IADLs (Out of 5)	0.411	0.457	0.421	0.679
Arthritis	0.578	0.715	0.617	0.750
Cancer	0.189	0.244	0.157	0.196
Lung disease	0.118	0.127	0.137	0.217
Diabetes	0.166	0.242	0.157	0.283
Heart Disease	0.396	0.364	0.402	0.427
High Blood Pressure	0.583	0.671	0.619	0.703
Psychiatric Condition	0.132	0.174	0.158	0.262
Stroke	0.096	0.094	0.111	0.135
Health 4 Years Prior				
ADLs	0.381	0.255	0.424	0.546
IADLs	0.185	0.205	0.218	0.393
Diabetes	0.135	0.214	0.131	0.254
Heart Disease	0.312	0.302	0.345	0.352
High Blood Pressure	0.519	0.640	0.559	0.662
Psychiatric Condition	0.118	0.162	0.142	0.271
Stroke	0.065	0.066	0.064	0.099
Other Health Factors				
Ever Smoked	0.674	0.565	0.741	0.594
BMI: <20 (Underweight)	0.015	0.036	0.025	0.032
BMI: 20-24.9 (Normal Weight)	0.261	0.342	0.361	0.364
BMI: 25-29.9 (Overweight)	0.461	0.410	0.397	0.339
BMI: 30+ (Obese)	0.262	0.212	0.216	0.265

Notes: Sample includes non-Hispanic white population aged 70+ only. The sample size is 3,147 participants in England and 5,330 participants in the United States. The age-gender standardized mean is presented for each variable, apart from income and wealth variables where age-gender standardised medians values are displayed. All amounts deflated to 2016 £s. Dollar amounts converted to £ using 1 $ = 0.75 £. Age is top coded at 99.

For peer review only

Table A4: Indexes of Inequality

	Slope Index of Inequality (SII)			Relative Index of Inequality (RII)		
	England	United States	Difference	England	United States	Difference
Income	-0.062	-0.085	-0.023	1.946	2.341	1.203
	[-0.097 to -0.028]	[-0.114 to -0.057]	(0.31)	[1.333 to 2.841]	[1.774 to 3.089]	(0.44)
Education	-0.087	-0.099	-0.011	3.001	2.820	0.940
	[-0.120 to -0.055]	[-0.126 to -0.072]	(0.56)	[2.013 to 4.474]	[2.131 to 3.731]	(0.80)
Wealth	-0.069	-0.096	-0.026	2.157	2.730	1.265
	[-0.104 to -0.035]	[-0.123 to -0.069]	(0.24)	[1.467 to 3.174]	[2.070 to 3.601]	(0.33)
NH Wealth	-0.071	-0.093	-0.022	2.213	2.311	1.044
	[-0.106 to -0.035]	[-0.124 to -0.062]	(0.36)	[1.463 to 3.348]	[1.748 to 3.056]	(0.87)
**Excluding Most Disadvantaged Group**						
Income	-0.067	-0.060	0.007	2.104	1.833	0.871
	[-0.107 to -0.027]	[-0.093 to -0.027]	(0.79)	[1.343 to 3.296]	[1.313 to 2.560]	(0.63)
Education	-0.070	-0.062	0.008	2.509	1.932	0.770
	[-0.107 to -0.032]	[-0.094 to -0.030]	(0.76)	[1.548 to 4.065]	[1.378 to 2.708]	(0.39)
Wealth	-0.065	-0.059	0.005	2.092	1.887	0.902
	[-0.104 to -0.025]	[-0.091 to -0.028]	(0.84)	[1.319 to 3.317]	[1.349 to 2.639]	(0.72)
NH Wealth	-0.064	-0.075	-0.012	2.077	1.981	0.954
	[-0.107 to -0.020]	[-0.110 to -0.040]	(0.69)	[1.217 to 3.544]	[1.433 to 2.740]	(0.88)
**Full Sample with Controls**						
Income	-0.040	-0.051	-0.011	1.499	1.698	1.133
	[-0.075 to -0.004]	[-0.079 to -0.023]	(0.63)	[1.028 to 2.187]	[1.275 to 2.263]	(0.61)
Education	-0.0336	-0.056	-0.022	1.308	1.742	1.332
	[-0.0754 to 0.008]	[-0.086 to -0.026]	(0.40)	[0.899 to 1.903]	[1.306 to 2.324]	(0.24)
Wealth	-0.060	-0.045	0.015	1.777	1.483	0.834
	[-0.097 to -0.023]	[-0.074 to -0.015]	(0.53)	[1.224 to 2.582]	[1.131 to 1.945]	(0.44)
NH Wealth	-0.050	-0.051	-0.002	1.562	1.614	1.033
	[-0.087 to -0.012]	[-0.080 to -0.023]	(0.94)	[1.078 to 2.262]	[1.231 to 2.116]	(0.89)

Notes: This table shows the Slope Index of Inequality (SII) and Relative Index of Inequality (RII), calculated using generalized linear models (log binomial regression) with identity and logarithmic link functions, respectively. The SIIs are displayed graphically in Figure 1. The estimates of RII are risk ratios. P-values are displayed in parentheses under differences. The panel 'Excluding Most Disadvantaged Group' excludes individuals in the bottom income decile. The panel 'Full Sample with Controls' controls for whether an individual had any cardiometabolic diseases (diabetes, heart disease, and stroke) and/or psychiatric conditions 4 years prior, whether an individual has ever smoked, and Body Mass Index (BMI) from at least 10 years prior.

For peer review only - http://bmjopen.bmj.com/site/about/guidelines.xhtml

Table A5: Difference in Prevalence of Dementia with Controls, United States vs England, 2017

Whole Sample:					
England Prevalence	0.097	0.088	0.086	0.089	0.092
	(0.089 to 0.106)	(0.078 to 0.098)	(0.073 to 0.099)	(0.072 to 0.106)	(0.072 to 0.111)
United States	0.112	0.100	0.098	0.101	0.120
	(0.106 to 0.118)	(0.092 to 0.108)	(0.087 to 0.109)	(0.087 to 0.114)	(0.104 to 0.135)
Percentage Point Difference	1.43	1.15	1.21	1.18	2.82
p-value	0.0055	0.025	0.020	0.034	<0.0001
Excluding Lowest Income Decile:					
England Prevalence	0.094	0.086	0.086	0.090	0.094
	(0.085 to 0.103)	(0.076 to 0.096)	(0.073 to 0.1)	(0.073 to 0.108)	(0.074 to 0.114)
United States	0.103	0.093	0.094	0.098	0.115
	(0.097 to 0.109)	(0.085 to 0.102)	(0.083 to 0.105)	(0.084 to 0.113)	(0.099 to 0.131)
Percentage Point Difference	0.89	0.74	0.75	0.81	2.09
p-value	0.11	0.18	0.18	0.18	0.0021
Lowest Income Decile:					
England Prevalence	0.114	0.107	0.088	0.082	0.095
	(0.089 to 0.139)	(0.074 to 0.14)	(0.036 to 0.14)	(0.021 to 0.144)	(0.025 to 0.164)
United States	0.187	0.162	0.148	0.131	0.163
	(0.166 to 0.208)	(0.129 to 0.195)	(0.103 to 0.192)	(0.078 to 0.184)	(0.103 to 0.223)
Percentage Point Difference	7.27	5.45	5.93	4.85	6.85
p-value	<0.0001	0.0020	0.0011	0.012	0.0015
Control for:					
Past Health Conditions		✓	✓	✓	✓
Ever Smoked			✓	✓	✓
Past BMI				✓	✓
Education					✓

Notes: Sample includes non-Hispanic white population aged 70+ only. The sample size is 3,147 participants in England and 5,330 participants in the United States. All estimates are age-gender standardized to the overall 2016 aged 70+ white population in England. The difference is calculated as the prevalence in the US minus prevalence in England. Differences are displayed as percentage points. 'Past Health Conditions' controls for whether an individual had any cardiometabolic diseases (diabetes, heart disease, and stroke) and/or psychiatric conditions 4 years prior. 'Ever Smoked' controls for whether an individual has ever smoked. BMI stands for Body Mass Index. 'Past BMI' includes dummy variables to control for whether an individual is classed as underweight, normal weight, *overweight* or *obese*. BMI measurements are based on when an individual first entered the survey, which is at least 10 years prior. We assume the effect of each control is constant across countries and age/gender groups. The reference groups are: No past health conditions; never smoked; normal weight; and high-school education.

For peer review only

For peer review only - http://bmjopen.bmj.com/site/about/guidelines.xhtml

Table A6: Difference in Prevalence of Dementia with Controls, United States vs England, 2017

	England			United States		
Whole Sample:						
Prevalence	0.097	0.092	0.089	0.112	0.105	0.101
CI	(0.089 to 0.106)	(0.082 to 0.102)	(0.072 to 0.106)	(0.106 to 0.118)	(0.098 to 0.113)	(0.087 to 0.114)
Income Decile:						
1 *Prevalence*	0.114	0.112	0.082	0.187	0.173	0.131
CI	(0.089 to 0.139)	(0.079 to 0.144)	(0.021 to 0.144)	(0.166 to 0.208)	(0.142 to 0.205)	(0.078 to 0.184)
2 *Prevalence*	0.113	0.119	0.118	0.141	0.145	0.148
CI	(0.09 to 0.136)	(0.088 to 0.149)	(0.05 to 0.187)	(0.119 to 0.163)	(0.113 to 0.176)	(0.086 to 0.21)
3 *Prevalence*	0.124	0.107	0.130	0.111	0.089	0.093
CI	(0.097 to 0.151)	(0.074 to 0.139)	(0.073 to 0.187)	(0.095 to 0.127)	(0.067 to 0.11)	(0.048 to 0.137)
4 *Prevalence*	0.099	0.095	0.094	0.118	0.114	0.111
CI	(0.071 to 0.126)	(0.065 to 0.126)	(0.041 to 0.148)	(0.099 to 0.137)	(0.088 to 0.14)	(0.062 to 0.16)
5 *Prevalence*	0.094	0.087	0.061	0.086	0.084	0.063
CI	(0.072 to 0.116)	(0.062 to 0.112)	(0.024 to 0.098)	(0.069 to 0.102)	(0.063 to 0.104)	(0.035 to 0.09)
6 *Prevalence*	0.098	0.097	0.116	0.108	0.107	0.100
CI	(0.07 to 0.127)	(0.064 to 0.131)	(0.056 to 0.177)	(0.088 to 0.128)	(0.081 to 0.134)	(0.053 to 0.148)
7 *Prevalence*	0.068	0.066	0.073	0.100	0.101	0.104
CI	(0.042 to 0.093)	(0.038 to 0.094)	(0.021 to 0.124)	(0.078 to 0.122)	(0.073 to 0.129)	(0.054 to 0.154)
8 *Prevalence*	0.083	0.084	0.097	0.082	0.078	0.085
CI	(0.053 to 0.114)	(0.051 to 0.117)	(0.054 to 0.14)	(0.066 to 0.097)	(0.058 to 0.098)	(0.054 to 0.117)
9 *Prevalence*	0.082	0.076	0.100	0.093	0.085	0.107
CI	(0.041 to 0.122)	(0.034 to 0.118)	(0.048 to 0.151)	(0.071 to 0.116)	(0.06 to 0.111)	(0.07 to 0.145)
10 *Prevalence*	0.059	0.058	0.051	0.077	0.077	0.068
CI	(0.034 to 0.083)	(0.029 to 0.087)	(0.015 to 0.087)	(0.052 to 0.103)	(0.051 to 0.103)	(0.036 to 0.1)
Control for:						
Past Health Conditions		✓	✓		✓	✓
Ever Smoked			✓			✓
Past BMI			✓			✓

Notes: Sample includes non-Hispanic white population aged 70+ only. The sample size is 3,147 participants in England and 5,330 participants in the United States. All estimates are age-gender standardized to the overall 2016 aged 70+ white population in England. 'Past Health Conditions' controls for whether an individual had any cardiometabolic diseases (diabetes, heart disease, and stroke) and/or psychiatric conditions 4 years prior. 'Ever Smoked' controls for whether an individual has ever smoked. BMI stands for Body Mass Index. 'Past BMI' includes dummy variables to control for whether an individual is classed as underweight, normal weight, *overweight* or *obese*. BMI measurements are based on when an individual first entered the survey, which is at least 10 years prior. We assume the effect of each control is constant across countries and age/gender groups. The reference groups are: No past health conditions; never smoked; and normal weight.

For peer review only

Figure A1: SES Gradient of Dementia, US vs England, Controlling for Past Health

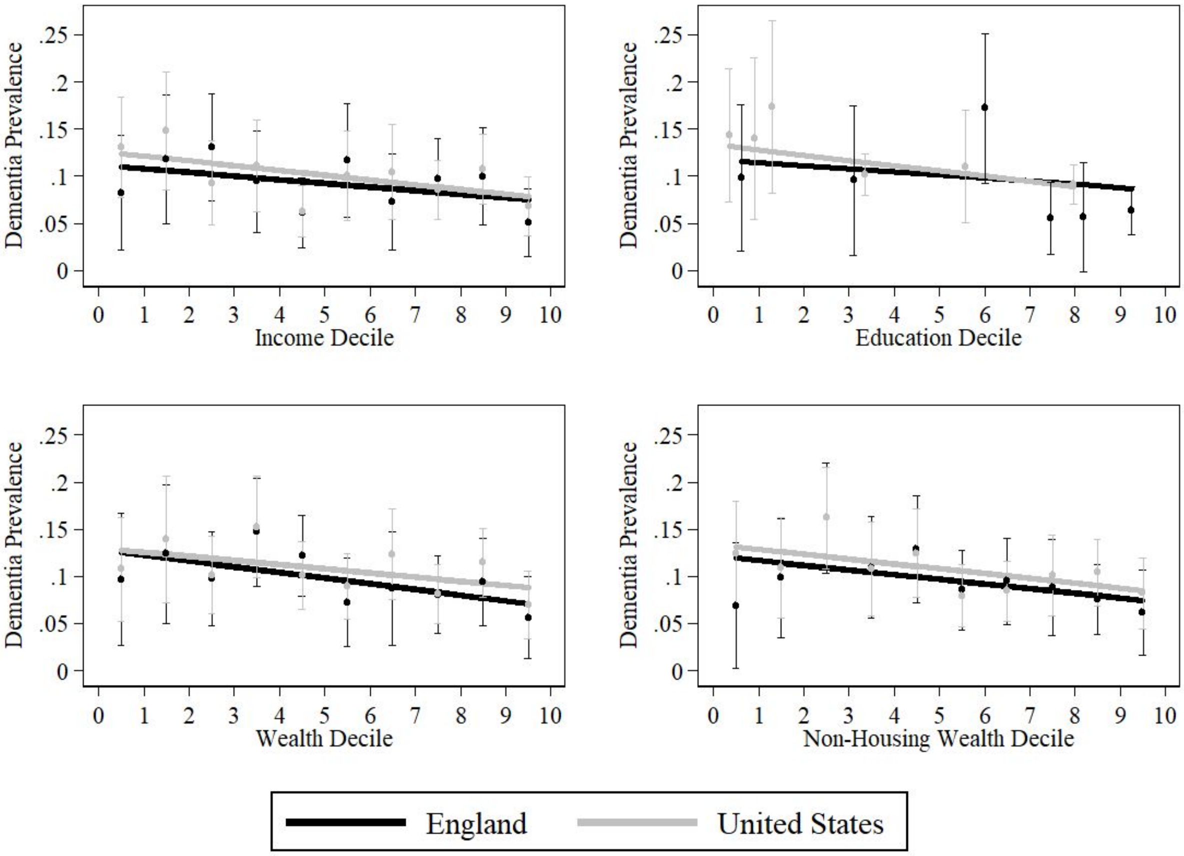

Notes: The points in this figure represent the mean age-gender standardized dementia prevalence for each country by socioeconomic status (SES) after controlling for a range of past health factors, along with 95% confidence interval for these predictions. The solid lines represent the fitted Socioeconomic Index of Inequality (SII) for each country. The values of the SII and the corresponding Relative Index of Inequality (RII) are listed in Table A4.

For peer review only - http://bmjopen.bmj.com/site/about/guidelines.xhtml

Table A7: Prevalence of Dementia, United States vs. England, Full-Sample

	England		United States			
	Age-Gender Standardized Prevalence [95% CI]		Age-Gender Standardized Prevalence [95% CI]		Difference	p-value
All	0.099	[0.091,0.108]	0.124	[0.118,0.129]	0.024	<0.0001
Household Income Decile						
1 (Lowest)	0.116	[0.091,0.140]	0.211	[0.192,0.230]	0.095	<0.0001
2	0.126	[0.101,0.152]	0.161	[0.144,0.178]	0.035	0.025
3	0.124	[0.098,0.150]	0.141	[0.120,0.162]	0.017	0.32
4	0.100	[0.072,0.127]	0.143	[0.124,0.162]	0.044	0.0094
5	0.101	[0.078,0.124]	0.097	[0.082,0.111]	-0.004	0.76
6	0.102	[0.072,0.131]	0.104	[0.086,0.121]	0.002	0.91
7	0.061	[0.043,0.079]	0.108	[0.088,0.127]	0.047	0.00062
8	0.088	[0.057,0.118]	0.085	[0.069,0.102]	-0.002	0.91
9	0.081	[0.041,0.122]	0.089	[0.070,0.108]	0.008	0.74
10 (Highest)	0.057	[0.034,0.080]	0.076	[0.055,0.098]	0.020	0.22
Years of Schooling						
9 or less	0.128	[0.103,0.153]	0.212	[0.195,0.230]	0.084	<0.0001
10	0.099	[0.078,0.119]	0.143	[0.118,0.167]	0.044	0.0072
11	0.097	[0.079,0.116]	0.119	[0.093,0.144]	0.021	0.19
12	0.069	[0.041,0.097]	0.134	[0.124,0.143]	0.064	<0.0001
13	0.063	[0.040,0.085]	0.118	[0.095,0.141]	0.055	0.00071
14 or more	0.055	[0.039,0.071]	0.086	[0.078,0.094]	0.031	0.00071
Household Wealth Decile						
1 (Lowest)	0.172	[0.136,0.207]	0.219	[0.198,0.241]	0.047	0.024
2	0.118	[0.093,0.144]	0.169	[0.151,0.188]	0.051	0.0014
3	0.105	[0.078,0.132]	0.138	[0.120,0.155]	0.032	0.049
4	0.090	[0.068,0.111]	0.120	[0.105,0.135]	0.030	0.023
5	0.118	[0.093,0.143]	0.116	[0.100,0.132]	-0.002	0.89
6	0.077	[0.056,0.099]	0.097	[0.084,0.110]	0.020	0.13
7	0.073	[0.047,0.098]	0.092	[0.076,0.107]	0.019	0.21
8	0.093	[0.067,0.118]	0.103	[0.086,0.120]	0.010	0.52
9	0.080	[0.047,0.113]	0.101	[0.084,0.118]	0.021	0.27
10 (Highest)	0.060	[0.040,0.081]	0.074	[0.058,0.089]	0.013	0.30
Household Non-Housing Wealth Decile						
1 (Lowest)	0.147	[0.113,0.180]	0.215	[0.195,0.235]	0.068	0.00055
2	0.114	[0.089,0.140]	0.184	[0.165,0.203]	0.070	<0.0001
3	0.121	[0.095,0.148]	0.148	[0.129,0.168]	0.027	0.11
4	0.100	[0.076,0.125]	0.129	[0.112,0.147]	0.029	0.054
5	0.110	[0.084,0.135]	0.104	[0.088,0.119]	-0.006	0.70
6	0.093	[0.067,0.118]	0.099	[0.085,0.113]	0.006	0.66
7	0.098	[0.072,0.124]	0.085	[0.071,0.098]	-0.013	0.37
8	0.079	[0.052,0.106]	0.090	[0.075,0.105]	0.011	0.48
9	0.057	[0.035,0.078]	0.094	[0.077,0.110]	0.037	0.0076
10 (Highest)	0.065	[0.045,0.085]	0.083	[0.067,0.099]	0.018	0.15

Notes: This table presents the estimates as Table 1 for the full sample aged 70+, including non-whites. The US sample includes 5,330 non-Hispanic white participants and 1,835 non-white participants. The England sample includes 3,147 non-Hispanic white participants and 77 non-white participants. All estimates are age-gender standardized to the overall 2016 aged 70+ white population in England. The difference is calculated as the prevalence in the US minus prevalence in England.

For peer review only - http://bmjopen.bmj.com/site/about/guidelines.xhtml

Table A9: Prevalence of Dementia in United States, Whites versus Non-whites

Table A8: Difference in Prevalence of Dementia with Controls, Full Sample

Whole Sample:					
England Prevalence	0.099	0.088	0.088	0.084	0.085
	[0.091 to 0.108]	[0.079 to 0.098]	[0.076 to 0.1]	[0.068 to 0.1]	[0.068 to 0.103]
United States	0.124	0.107	0.107	0.105	0.123
	[0.118 to 0.129]	[0.1 to 0.114]	[0.097 to 0.117]	[0.093 to 0.118]	[0.109 to 0.137]
Percentage Point Difference	2.44	1.87	1.90	2.14	3.80
p-value	<0.0001	<0.0001	0.00015	0.00011	<0.0001
Excluding Lowest Income Decile:					
England Prevalence	0.096	0.086	0.086	0.087	0.090
	[0.087 to 0.105]	[0.076 to 0.096]	[0.073 to 0.099]	[0.071 to 0.103]	[0.072 to 0.108]
United States	0.113	0.098	0.099	0.100	0.117
	[0.107 to 0.118]	[0.091 to 0.106]	[0.089 to 0.108]	[0.086 to 0.113]	[0.103 to 0.132]
Percentage Point Difference	1.64	1.25	1.27	1.24	2.70
p-value	0.0024	0.020	0.019	0.030	<0.0001
Lowest Income Decile:					
England Prevalence	0.116	0.107	0.106	0.078	0.099
	[0.091 to 0.140]	[0.077 to 0.138]	[0.062 to 0.15]	[0.026 to 0.13]	[0.036 to 0.162]
United States	0.211	0.187	0.186	0.161	0.195
	[0.192 to 0.230]	[0.16 to 0.214]	[0.149 to 0.223]	[0.117 to 0.206]	[0.138 to 0.252]
Percentage Point Difference	9.54	7.93	7.96	8.37	9.60
p-value	<0.0001	<0.0001	<0.0001	<0.0001	<0.0001
Control for:					
Past Health Conditions		✓	✓	✓	✓
Ever Smoked			✓	✓	✓
Past BMI				✓	✓
Education					✓

Notes: Full sample, population aged 70+, including minorities. The sample sizes are 3,224 participants in England, and 7,165 participants in the United States. All estimates are age-gender standardized to the overall 2016 aged 70+ white population in England. The difference is calculated as the prevalence in the US minus prevalence in England. Differences are displayed as percentage points. 'Past Health Conditions' controls for whether an individual had any cardiometabolic diseases (diabetes, heart disease, and stroke) and/or psychiatric conditions 4 years prior. 'Ever Smoked' controls for whether an individual has ever smoked. BMI stands for Body Mass Index. 'Past BMI' includes dummy variables to control for whether an individual is classed as underweight, normal weight, *overweight* or *obese*. BMI measurements are based on when an individual first entered the survey, which is at least 10 years prior. We assume the effect of each control is constant across countries and age/gender groups. The reference groups are: No past health conditions; never smoked; normal weight; and high-school education.

For peer review only

For peer review only - http://bmjopen.bmj.com/site/about/guidelines.xhtml

	US Non-White and Hispanic		US Non-Hispanic White			
	Age-Gender Standardized Prevalence [95% CI]		Age-Gender Standardized Prevalence [95% CI]		Difference	p-value
All	0.182	[0.169 to 0.195]	0.112	[0.106 to 0.118]	-0.070	<0.0001
Household Income Decile						
1 (Lowest)	0.224	[0.199 to 0.249]	0.204	[0.178 to 0.231]	-0.020	0.28
2	0.211	[0.180 to 0.243]	0.143	[0.123 to 0.163]	-0.068	0.00034
3	0.220	[0.158 to 0.282]	0.121	[0.102 to 0.140]	-0.099	0.0023
4	0.202	[0.165 to 0.240]	0.133	[0.111 to 0.155]	-0.069	0.0018
5	0.116	[0.076 to 0.156]	0.094	[0.079 to 0.109]	-0.022	0.31
6	0.164	[0.097 to 0.230]	0.098	[0.081 to 0.116]	-0.065	0.065
7	0.129	[0.080 to 0.178]	0.107	[0.086 to 0.128]	-0.022	0.42
8	0.107	[0.047 to 0.166]	0.084	[0.067 to 0.101]	-0.023	0.47
9	0.072	[0.026 to 0.118]	0.090	[0.069 to 0.110]	0.017	0.50
10 (Highest)	0.084	[0.038 to 0.129]	0.077	[0.055 to 0.099]	-0.006	0.81
Years of Schooling						
9 or less	0.235	[0.212 to 0.258]	0.190	[0.162 to 0.218]	-0.045	0.015
10	0.172	[0.127 to 0.218]	0.137	[0.109 to 0.165]	-0.035	0.19
11	0.144	[0.109 to 0.179]	0.109	[0.080 to 0.139]	-0.035	0.14
12	0.198	[0.170 to 0.227]	0.124	[0.114 to 0.133]	-0.074	<0.0001
13	0.159	[0.101 to 0.218]	0.116	[0.090 to 0.141]	-0.043	0.18
14 or more	0.099	[0.079 to 0.119]	0.085	[0.076 to 0.093]	-0.015	0.20
Household Wealth Decile						
1 (Lowest)	0.228	[0.201 to 0.255]	0.208	[0.176 to 0.240]	-0.020	0.36
2	0.208	[0.179 to 0.237]	0.154	[0.131 to 0.177]	-0.054	0.0043
3	0.184	[0.142 to 0.227]	0.120	[0.102 to 0.138]	-0.064	0.0063
4	0.142	[0.111 to 0.174]	0.115	[0.098 to 0.132]	-0.027	0.13
5	0.159	[0.117 to 0.202]	0.108	[0.092 to 0.124]	-0.051	0.027
6	0.120	[0.084 to 0.155]	0.093	[0.080 to 0.107]	-0.026	0.18
7	0.156	[0.086 to 0.226]	0.088	[0.072 to 0.103]	-0.068	0.063
8	0.197	[0.134 to 0.259]	0.099	[0.081 to 0.116]	-0.098	0.0031
9	0.122	[0.065 to 0.178]	0.099	[0.082 to 0.117]	-0.022	0.46
10 (Highest)	0.045	[0.018 to 0.073]	0.075	[0.059 to 0.090]	0.029	0.071
Household Non-Housing Wealth Decile						
1 (Lowest)	0.231	[0.207 to 0.256]	0.197	[0.167 to 0.227]	-0.034	0.085
2	0.204	[0.177 to 0.231]	0.177	[0.151 to 0.203]	-0.027	0.16
3	0.187	[0.142 to 0.232]	0.135	[0.114 to 0.155]	-0.052	0.036
4	0.158	[0.128 to 0.188]	0.123	[0.104 to 0.143]	-0.034	0.059
5	0.106	[0.069 to 0.143]	0.102	[0.086 to 0.118]	-0.004	0.85
6	0.099	[0.063 to 0.134]	0.098	[0.084 to 0.113]	0.000	0.99
7	0.124	[0.066 to 0.182]	0.083	[0.069 to 0.097]	-0.040	0.18
8	0.140	[0.069 to 0.210]	0.088	[0.073 to 0.103]	-0.051	0.16
9	0.127	[0.054 to 0.201]	0.092	[0.075 to 0.108]	-0.035	0.36
10 (Highest)	0.069	[0.050 to 0.088]	0.084	[0.068 to 0.101]	0.016	0.23

Notes: The sample includes 5,330 non-Hispanic white participants and 1,835 non-white and Hispanic participants. All estimates are age-gender standardized to the overall 2016 aged 70+ white population in England. The difference is calculated as the non-white and Hispanic prevalence in the US minus the non-Hispanic white prevalence in the US.

For peer review only

Appendix References

1 Langa KM, Larson EB, Crimmins EM, *et al.* A comparison of the prevalence of dementia in the United States in 2000 and 2012. *JAMA Intern Med* 2017;**177**:51–58.

2 Hudomiet P, Hurd MD, Rohwedder S. The relationship between lifetime out-of-pocket medical expenditures, dementia, and socioeconomic status in the US. *J Econ Ageing* 2019;**14**:100181.

3 Rusmaully J, Dugravot A, Moatti J-P, *et al.* Contribution of cognitive performance and cognitive decline to associations between socioeconomic factors and dementia: A cohort study. *PLoS Med* 2017;**14**:e1002334.

4 Cadar D, Lassale C, Davies H, *et al.* Individual and area-based socioeconomic factors associated with dementia incidence in England: evidence from a 12-year follow-up in the English longitudinal study of ageing. *JAMA Psychiatry* 2018;**75**:723–732.

5 Rocca WA, Petersen RC, Knopman DS, *et al.* Trends in the incidence and prevalence of Alzheimer's disease, dementia, and cognitive impairment in the United States. *Alzheimers Dement* 2011;**7**:80–93.

6 Basu R. Education and dementia risk: results from the Aging Demographics and Memory Study. *Res Aging* 2013;**35**:7–31.

7 Nguyen TT, Tchetgen EJT, Kawachi I, *et al.* Instrumental variable approaches to identifying the causal effect of educational attainment on dementia risk. *Ann Epidemiol* 2016;**26**:71–76.

8 Crimmins EM, Kim JK, Langa KM, *et al.* Assessment of cognition using surveys and neuropsychological assessment: the Health and Retirement Study and the Aging, Demographics, and Memory Study. *J Gerontol B Psychol Sci Soc Sci* 2011;**66**:i162–i171.

9 Garcia MA, Saenz J, Downer B, *et al.* The role of education in the association between race/ethnicity/nativity, cognitive impairment, and dementia among older adults in the United States. *Demogr Res* 2018;**38**:155.

10 Weden MM, Shih RA, Kabeto MU, *et al.* Secular trends in dementia and cognitive impairment of US rural and urban older adults. *Am J Prev Med* 2018;**54**:164–172.

11 Steptoe A, Breeze E, Banks J, *et al.* Cohort profile: the English longitudinal study of ageing. *Int J Epidemiol* 2013;**42**:1640–1648.

12 Hurd MD, Martorell P, Delavande A, *et al.* Monetary costs of dementia in the United States. *N Engl J Med* 2013;**368**:1326–1334.

13 Langa KM, Plassman BL, Wallace RB, *et al.* The Aging, Demographics, and Memory Study: study design and methods. *Neuroepidemiology* 2005;**25**:181–191.

14 Gianattasio KZ, Wu Q, Glymour MM, *et al.* Comparison of methods for algorithmic classification of dementia status in the health and retirement study. *Epidemiol Camb Mass* 2019;**30**:291.

For peer review only - http://bmjopen.bmj.com/site/about/guidelines.xhtml

STROBE (Strengthening The Reporting of OBservational Studies in Epidemiology) Checklist

A checklist of items that should be included in reports of observational studies. You must report the page number in your manuscript where you consider each of the items listed in this checklist. If you have not included this information, either revise your manuscript accordingly before submitting or note N/A.

Note: An Explanation and Elaboration article discusses each checklist item and gives methodological background and published examples of transparent reporting. The STROBE checklist is best used in conjunction with this article (freely available on the Web sites of PLoS Medicine at http://www.plosmedicine.org/, Annals of Internal Medicine at http://www.annals.org/, and Epidemiology at http://www.epidem.com/). Information on the STROBE Initiative is available at www.strobe-statement.org.

Section and Item	Item No.	Recommendation	Reported on Page No.
**Title and Abstract**	1	(*a*) Indicate the study's design with a commonly used term in the title or the abstract	1
		(*b*) Provide in the abstract an informative and balanced summary of what was done and what was found	1
**Introduction**			
Background/Rationale	2	Explain the scientific background and rationale for the investigation being reported	3
Objectives	3	State specific objectives, including any prespecified hypotheses	3
**Methods**			
Study Design	4	Present key elements of study design early in the paper	3-4
Setting	5	Describe the setting, locations, and relevant dates, including periods of recruitment, exposure, follow-up, and data collection	3-4
Participants	6	(*a*) *Cohort study*—Give the eligibility criteria, and the sources and methods of selection of participants. Describe methods of follow-up	3
		Case-control study—Give the eligibility criteria, and the sources and methods of case ascertainment and control selection. Give the rationale for the choice of cases and controls	
		Cross-sectional study—Give the eligibility criteria, and the sources and methods of selection of participants	
		(*b*) *Cohort study*—For matched studies, give matching criteria and number of exposed and unexposed	
		Case-control study—For matched studies, give matching criteria and the number of controls per case	
Variables	7	Clearly define all outcomes, exposures, predictors, potential confounders, and effect modifiers. Give diagnostic criteria, if applicable	4-5

For peer review only

Section and Item	Item No.	Recommendation	Reported on Page No.
Data Sources/ Measurement	8*	For each variable of interest, give sources of data and details of methods of assessment (measurement). Describe comparability of assessment methods if there is more than one group	4
Bias	9	Describe any efforts to address potential sources of bias	4-5
Study Size	10	Explain how the study size was arrived at	3
Quantitative Variables	11	Explain how quantitative variables were handled in the analyses. If applicable, describe which groupings were chosen and why	4-5
Statistical Methods	12	(*a*) Describe all statistical methods, including those used to control for confounding	4-5
		(*b*) Describe any methods used to examine subgroups and interactions	4-5
		(*c*) Explain how missing data were addressed	4-5
		(*d*) *Cohort study*—If applicable, explain how loss to follow-up was addressed	4-5
		Case-control study—If applicable, explain how matching of cases and controls was addressed	
		Cross-sectional study—If applicable, describe analytical methods taking account of sampling strategy	
		(*e*) Describe any sensitivity analyses	4-5
**Results**			
Participants	13*	(a) Report numbers of individuals at each stage of study—eg numbers potentially eligible, examined for eligibility, confirmed eligible, included in the study, completing follow-up, and analysed	9
		(b) Give reasons for non-participation at each stage	
		(c) Consider use of a flow diagram	
Descriptive Data	14*	(a) Give characteristics of study participants (eg demographic, clinical, social) and information on exposures and potential confounders	6,11
		(b) Indicate number of participants with missing data for each variable of interest	
		(c) *Cohort study*—Summarise follow-up time (eg, average and total amount)	
Outcome Data	15*	*Cohort study*—Report numbers of outcome events or summary measures over time	5-6
		Case-control study—Report numbers in each exposure category, or summary measures of exposure	
		Cross-sectional study—Report numbers of outcome events or summary measures	

For peer review only

Section and Item	Item No.	Recommendation	Reported on Page No.
Main Results	16	(*a*) Give unadjusted estimates and, if applicable, confounder-adjusted estimates and their precision (eg, 95% confidence interval). Make clear which confounders were adjusted for and why they were included	5-6
		(*b*) Report category boundaries when continuous variables were categorized	
		(*c*) If relevant, consider translating estimates of relative risk into absolute risk for a meaningful time period	10
Other Analyses	17	Report other analyses done—eg analyses of subgroups and interactions, and sensitivity analyses	5-6,9-11
**Discussion**			
Key Results	18	Summarise key results with reference to study objectives	6-7
Limitations	19	Discuss limitations of the study, taking into account sources of potential bias or imprecision. Discuss both direction and magnitude of any potential bias	8
Interpretation	20	Give a cautious overall interpretation of results considering objectives, limitations, multiplicity of analyses, results from similar studies, and other relevant evidence	7
Generalisability	21	Discuss the generalisability (external validity) of the study results	7
**Other Information**			
Funding	22	Give the source of funding and the role of the funders for the present study and, if applicable, for the original study on which the present article is based	12

*Give information separately for cases and controls in case-control studies and, if applicable, for exposed and unexposed groups in cohort and cross-sectional studies.

Once you have completed this checklist, please save a copy and upload it as part of your submission. DO NOT include this checklist as part of the main manuscript document. It must be uploaded as a separate file.

For peer review only

BMJ Open

Dementia and Disadvantage in the United States and England: Population-based comparative study

Journal:	*BMJ Open*
Manuscript ID	bmjopen-2020-045186.R1
Article Type:	Original research
Date Submitted by the Author:	06-Mar-2021
Complete List of Authors:	Arapakis, Karolos; UCL, Department of Economics<br>Brunner, Eric; University College London, Epidemiology and Public Health<br>French, Eric; UCL, Department of Economics; Institute for Fiscal Studies<br>McCauley, Jeremy; University of Bristol, School of Economics
**Primary Subject Heading**:	Public health
Secondary Subject Heading:	Health economics, Geriatric medicine, Neurology
Keywords:	Dementia < NEUROLOGY, PUBLIC HEALTH, HEALTH ECONOMICS

SCHOLARONE™
Manuscripts

I, the Submitting Author has the right to grant and does grant on behalf of all authors of the Work (as defined in the below author licence), an exclusive licence and/or a non-exclusive licence for contributions from authors who are: i) UK Crown employees; ii) where BMJ has agreed a CC-BY licence shall apply, and/or iii) in accordance with the terms applicable for US Federal Government officers or employees acting as part of their official duties; on a worldwide, perpetual, irrevocable, royalty-free basis to BMJ Publishing Group Ltd ("BMJ") its licensees and where the relevant Journal is co-owned by BMJ to the co-owners of the Journal, to publish the Work in this journal and any other BMJ products and to exploit all rights, as set out in our licence.

The Submitting Author accepts and understands that any supply made under these terms is made by BMJ to the Submitting Author unless you are acting as an employee on behalf of your employer or a postgraduate student of an affiliated institution which is paying any applicable article publishing charge ("APC") for Open Access articles. Where the Submitting Author wishes to make the Work available on an Open Access basis (and intends to pay the relevant APC), the terms of reuse of such Open Access shall be governed by a Creative Commons licence – details of these licences and which Creative Commons licence will apply to this Work are set out in our licence referred to above.

Other than as permitted in any relevant BMJ Author's Self Archiving Policies, I confirm this Work has not been accepted for publication elsewhere, is not being considered for publication elsewhere and does not duplicate material already published. I confirm all authors consent to publication of this Work and authorise the granting of this licence.

For peer review only

Dementia and Disadvantage in the United States and England: Population-based comparative study

By Karolos Arapakis, Eric J Brunner, Eric French, Jeremy McCauley*

Objectives – To compare dementia prevalence and how it varies by socioeconomic status (SES) across the United States and England.

Design – Population-based comparative study.

Setting - Non-Hispanic whites aged over 70 population in the United States and England.

Participants – Data from the Health and Retirement Study (HRS) and the English Longitudinal Study of Ageing (ELSA), which are harmonised, nationally representative panel studies. The sample includes 5,330 and 3,147 individuals in the United States and England, respectively.

Main outcome measures – Between country differences in age-gender standardized dementia prevalence, across the SES gradient. Dementia prevalence was estimated in each country using an algorithm based on an identical battery of demographic, cognitive, and functional measures.

Results – Dementia prevalence is higher among the disadvantaged in both countries, with the United States being more unequal according to four measures of SES. Overall prevalence was lower in England at 9.7% (95% CI, 8.9% to 10.6%) than the US at 11.2% (95% CI, 10.6% to 11.8%), a difference of 1.4 percentage points (pp) (p = 0.0055). Most of the between country difference is driven by the bottom of the SES distribution. In the lowest income decile individuals in the US had 7.3 pp (p <0.0001) higher prevalence than in England. Once past health factors and education were controlled for, most of the within country inequalities disappeared; however, the cross-country difference in prevalence for those in lowest income decile remained disproportionately high.

Conclusions – There is inequality in dementia prevalence according to income, wealth, and education in both the US and England. England has lower dementia prevalence and a less steep SES gradient. Most of the cross-country difference is concentrated in the lowest SES group, which provides evidence that disadvantage in the United States is a disproportionately high risk factor for dementia.

*Department of Economics, University College London, London, UK (K Arapakis MSc, Prof E French PhD)
Department of Epidemiology and Public Health, University College London, London, UK (Prof E J Brunner PhD)
Department of Economics, University of Bristol, Bristol, UK (J McCauley PhD)
Institute for Fiscal Studies, London, UK (E French)
Correspondence to: Jeremy McCauley, School of Economics, University of Bristol, Bristol BS8 1TU, UK **jeremy.mccauley@bristol.ac.uk**

For peer review only - http://bmjopen.bmj.com/site/about/guidelines.xhtml

Strengths and limitations of this study

- This is the first study to compare dementia prevalence across countries using the same survey methodology and the exact same measure of dementia. The surveys have similar sample selection and questionnaire design. We standardise our estimates by age and gender to the English population aged over 70 in 2016. Any differences in overall prevalence across the two countries should represent true differences.
- We measure the SES gradient of dementia across four different measures of SES: income; education; wealth; and non-housing wealth.
- Dementia disproportionately affects the most disadvantaged in in both countries, although the gradient is steeper in the US according to all four measures of SES.
- We do not ascertain dementia directly, but predict cases using a common battery of measures in ELSA and HRS. One of the SES measures, education, is also used as a predictor of dementia.

For peer review only

For peer review only - http://bmjopen.bmj.com/site/about/guidelines.xhtml

Introduction

Dementia, a severe and irreversible decline in memory and other cognitive functions, is a major and increasing global health challenge. It is the fifth leading cause of death globally and is one of the most common comorbidities for Covid-19 morbidity.[1,2] It results in large social and economic costs.[3,4]

Americans are more likely to be in poor health than their English counterparts in multiple dimensions, including heart disease and diabetes.[5] These differences are large along all points of the socioeconomic status (SES) gradient, although the gradient is generally steeper in the US. While the SES gradient for many diseases has been well established,[6–8] only a few studies have focused specifically on dementia.[9,10] The available evidence is summarized in Table A1 in the online appendix. The evidence of the SES gradient for dementia is also less clear, as in England while a strong association has been established between wealth and dementia incidence, the same was not observed for education level.[11]

The Global Burden of Disease Study (GBD) reported that in 2017 among those aged over 70, the US had a lower overall prevalence of dementia at 7.89% compared with the UK at 8.91%.[12] However, the GBD has identified substantial heterogeneity in case-ascertainment methods throughout the dementia literature, resulting in location-specific inconsistencies and potentially biased cross-country comparisons. This has led to calls for analyses with more consistent and comparable measures of dementia to inform policy makers, researchers, and clinicians about global differences in dementia.[13]

In this study, we compared dementia prevalence in England and the US among non-Hispanic whites aged 70+, and how it varied across the SES gradient of each country. Location-specific inconsistencies caused by differences in diagnostic practices were not an issue in our study because we used an identical case definition for dementia, and the surveys in the analysis shared the same design and sampling techniques. More specifically, we used two large surveys, the US Health and Retirement Study (HRS) and the English Longitudinal Study of Ageing (ELSA), that contain a battery of the same demographic, cognitive, and functional measures, and we applied the same prediction algorithm in both countries to detect undiagnosed as well as diagnosed cases. We compared dementia prevalence within and across England and the US using important indicators of SES, specifically: income; education; wealth; and non-housing wealth.

Methods

Description of Surveys

Data were extracted from the 2016 and earlier waves of the HRS and ELSA, which are nationally representative biennial surveys of the US and English populations, respectively.[14,15] Both the HRS and ELSA follow respondents longitudinally until death, with new cohorts entering to maintain population representativeness as the study sample gets older. The design of ELSA was based on the HRS, making the two surveys analogous, with both collecting data on health, ability, demographics, employment, and wealth. In addition to measuring health conditions and difficulties respondents have with Activities of Daily Living (ADLs) and Instrumental Activities of Daily Living (IADLs), sample members also have their cognitive function assessed. A range of tests adapted from the Telephone Interview for Cognitive Status (TICS) have been carried out in HRS since 1996 and ELSA since 2014. If a sample member was unable to respond in person, a proxy respondent was asked questions about the respondent's change in memory. Both surveys

For peer review only - http://bmjopen.bmj.com/site/about/guidelines.xhtml

have a high response rate, which is displayed in the online appendix Table A2. We describe these surveys in more detail in the online appendix.

Cohort Description

Our samples are restricted to non-Hispanic whites over the age of 70 years old that live in the community or in nursing homes in 2016. This provides a study sample of 5,330 participants in the HRS and 3,147 participants in ELSA. We restrict our sample to non-Hispanic whites to ensure estimates are comparable across countries. Summary statistics of both the raw and selected samples are displayed in online appendix Tables A3 and A4, and specifications that include ethnic minorities are also displayed.

Patient and Public Involvement

No study participants were involved in setting the research question or outcome measures, nor were they involved in any other area of the design, implementation, and analysis of the study. There are no direct plans to disseminate the results of the research to study participants.

Dementia Case Definition

The HRS included a detailed clinical substudy (ADAMS: Aging, Demographics and Memory Study) of 856 sample members aged 70+ who completed an in-depth in-home assessment of cognitive status conducted by experienced teams at the Duke University Dementia Epidemiology Research Center who diagnosed each participant as normal, cognitively impaired but not demented (CIND), or demented.[3] Data from ADAMS is regarded as the gold-standard dementia diagnoses against which to train algorithms to predict dementia.[16] Hurd et al. estimated separate ordered probit models in the ADAMS subsample for self- and proxy-respondents to generate a predictive algorithm for cognitive status, based on the ADAMS diagnoses, for the whole HRS sample. The algorithm uses a range of variables including demographic information, Activities of Daily Living (ADLs), Instrumental Activities of Daily Living (IADLs), TICS questionnaire, as well as the change in these variables across waves.[3] Proxy respondents had a separate predictive algorithm as they were asked a different set of questions from self-respondents, which included the short form of the Informant Questionnaire on Cognitive Decline in the Elderly (IQCODE). The use of a proxy to assess cognitive decline and dementia in elderly people is a recognised accepted standard method for identifying severe cognitive impairment and has been validated many times.[17] Importantly, the same set of questions used in the Hurd et al. algorithm is asked of self- and proxy-respondents in both HRS and ELSA. Summary statistics for a variety of predictors are displayed in online appendix Table A5.

We applied Hurd et al.'s predictive algorithm to estimate the probably of dementia for those in the HRS sample in 2016 and extended the prediction to the ELSA sample. The algorithm predicts the probability of dementia in the following year; therefore, we predicted dementia prevalence in 2017. Hurd et al's predictive algorithm has been shown to have an accuracy (percentage correctly classified as demented or non-demented) of 94%, sensitivity of 65%, and a specificity of 98% in the estimation sample.[16] An in-depth discussion of the predictive algorithm procedure can be found in the online appendix.

For peer review only - http://bmjopen.bmj.com/site/about/guidelines.xhtml

Non-response for people unable or unwilling to participate in the survey is important when attempting to estimate dementia prevalence across the population. While attrition exists in both surveys, it is unlikely to significantly affect our estimates since among older ELSA and HRS respondents there is no statistically significant correlation between attrition and prior health or the SES indicators of education, income and wealth.[18]

Measures of Socioeconomic Status

We considered four measures of SES: income; education; wealth; and non-housing wealth. Income is measured as current household income from all sources. For education we used total years of schooling. "Wealth is measured as the sum of all household reported savings, stocks, bonds, business wealth, other assets, and the value of housing assets (e.g., properties) after financial debt and mortgage debt has been subtracted. Non-housing wealth is the same measure as wealth but excludes housing assets and mortgage debt, and therefore measure wealth that can be more easily converted to cash. Wealth and non-housing wealth are both measured from 4 years prior to minimize reverse causality, as medical expenses associated with dementia are high and may run down wealth.[4] For each measure, we created a SES gradient by ranking individuals based on that measure. For income, wealth, and non-housing wealth, we assigned everyone to a decile in their respective country. For education, we ranked individuals according to their number of years of schooling.

Statistical Analysis

We created a pooled dataset of the two surveys. In our statistical analysis we used HRS and ELSA sampling weights to adjust for nonresponse and for the sampling design of the surveys. To make both within country estimates along the SES gradient and cross-country estimates directly comparable, estimates were age-gender standardized to the English population aged over 70 in 2016 using direct standardization, categorising the population into ten groups: five age bands (70-74; 75-79; 80-84; 85-89; and 90+) by gender. We estimated the prevalence of dementia in each country, their difference, and compared the prevalence along the four SES gradients. For each estimate presented we computed the corresponding 95% confidence interval (CI), and for any differences we computed the corresponding p-values. For each SES factor, as well as estimating the age-gender standardized prevalence along the gradient, we calculated the Relative Index of Inequality (RII) and Slope Index of Inequality (SII) using generalized linear models (log binomial regression) with logarithmic and identity link functions, respectively. The RII can be interpreted as the relative likelihood of dementia prevalence of those in the lowest SES group compared to those in the highest, and the SII can be interpreted as the absolute effect on dementia probability of moving from the lowest SES group to the highest.[19] To assess whether any observed differences could be explained by disparities in past health risk factors across countries we conditioned on a variety of risk factors and assessed how our estimates changed. Where possible, when conditioning on these factors we used past health instead of current health to address the problem of reverse causality: i.e., the problem that dementia may cause health problems such as low weight. Statistical analyses were performed using STATA software.

For peer review only - http://bmjopen.bmj.com/site/about/guidelines.xhtml

Results

Table 1 shows the age-gender standardized prevalence of dementia for the aged over 70 white non-Hispanic population in both England and the US. Dementia prevalence is lower in England at 9.7% (95% CI, 8.9% to 10.6%) than the US at 11.2% (95% CI, 10.6% to 11.8), a difference of 1.4 percentage points (pp) that is highly statistically significant (P = 0.0055).

Table 1 also shows dementia prevalence for different SES groups, in terms of income, education, wealth, and non-housing wealth. Regardless of the measure of the SES, there is a clear gradient in dementia prevalence, with the most disadvantaged groups in both England and the US having higher dementia prevalence. The gradient is steeper in the US and is driven by significantly higher dementia prevalence for those at the very bottom of the distribution. In the US, those in the lowest income decile have a dementia prevalence of 18.7% (95% CI, 16.6% to 20.8%), which is considerably higher than in England, with a prevalence among those in the lowest decile of 11.4% (95% CI, 8.9% to 13.9%). The difference is highly statistically significant (*P < 0.0001)*. For income deciles above the lowest, the difference across the two countries is much smaller and not statistically significant. This same general pattern is evident across the other measures of SES that we consider, although when using wealth, the difference between those in the bottom decile is not statistically significant.

Figure 1 presents the same dementia prevalence information shown in Table 1, but in graphical format. It also reports the Slope Index of Inequality (SII) for the four different measures of the SES for both countries. In both the US and England, dementia is more prevalent among the more disadvantaged. The gradient tends to be steeper in the US, corresponding to a larger (in absolute value) SII in the US for each SES measure. For income, the SII is -0.062 (95% CI, -0.097 to -0.028) and -0.085 (95% CI, -0.114 to -0.057) for England and the US, respectively. The SIIs are not statistically different. If the lowest income decile is excluded, the SII for England becomes slightly steeper (-0.067 (95% CI, -0.107 to -0.027)) whereas the SII for the US becomes less steep than England (-0.060 (95% CI, -0.093 to -0.027)).

Next, we attempted to understand the potential drivers of these gradients and the differences in the gradients across countries. We extended the analysis to account for cardiometabolic diseases (i.e. diabetes, heart disease and stroke), and behaviours (i.e. smoking and body mass index) as dementia risk factors.[1,20] Previous research showed these factors to be more prevalent in the US than England, especially among the most disadvantaged.[5] Table 2 displays the percentage point difference in dementia prevalence after we controlled for various measures of past health and behaviours. The results are split into three panels: the whole sample; the whole sample excluding those in the lowest income decile; and the lowest income decile. As was shown in Table 1, the difference in the prevalence of dementia between England and the US was 1.43 pp (*P = 0.0055*). Table 2 shows this difference declined to 0.894 pp (*P = 0.11)* when we excluded the lowest income decile. Controlling for past health and behaviours modestly reduced this cross-country difference further: the difference declined by a maximum of 19%. For the lowest income decile, controlling for past health and behaviour reduced the cross-country difference of 7.27 pp (*P < 0.0001*) by a more substantial 33%.

In the online appendix Tables A6-A8 we investigated whether past health and behaviour explained the SES gradient within each country. We found that in England and the US these factors accounted for most of

For peer review only - http://bmjopen.bmj.com/site/about/guidelines.xhtml

the SES gradient, as shown in online appendix Figure A1. However, in the US, prevalence in the lowest income decile remained disproportionately high.

Education has also been shown to be a risk factor for dementia. Table 1 shows that in both the US and England, the less educated have higher dementia prevalence. Controlling for education increased the estimated difference across countries, from 1.43 pp to 2.82 pp, as can be seen in the online appendix Table A7. Education cannot explain these differences since the English have lower educational attainment.

In our main analysis we exclude ethnic minorities, who have higher dementia prevalence and comprise a larger share of the US than the English population. Including minorities increased the estimated difference across countries, from 1.43 pp to 2.42 pp, as can be seen in the online appendix Tables A9 and A10. This is largely caused by the high prevalence of dementia among minorities in the US as displayed in online appendix Table A11.

Discussion

Main findings

Using nationally representative samples of older individuals from England and the US, and applying the same algorithmic procedure to predict dementia in both samples, we showed that in both the US and England dementia is more prevalent among the disadvantaged, and the SES gradient of dementia is steeper in the US. The steeper gradient in the US is largely driven by those in the lowest decile. In both countries, most of the SES gradient disappeared when we controlled for past health related factors, although prevalence for those in lowest income decile in the US remained disproportionately high. If the lowest income decile is excluded from our sample the difference in dementia prevalence across the countries is statistically insignificant, and the remaining SES gradient of dementia is remarkably similar across countries.

While poorer individuals face a higher burden of dementia in both England and the US, the extremely poor in the US face a disproportionately high burden of dementia. Controlling for past health-related factors can explain some, but not all, of the cross-country difference. It can explain up to around one third of the difference for those in the lowest income decile. While past health factors such as adiposity and smoking are correlated with dementia, those in the lowest income decile in the US do not smoke more or have higher BMIs than their English counterparts. Therefore, this cannot explain their disproportionately higher prevalence of dementia. Education also cannot explain the difference, as the US population is more educated at every income decile, and in fact the educational difference masks some of the underlying dementia risk difference between countries.

Adding minorities increased estimated dementia prevalence, especially in the US, because dementia prevalence is higher among minorities, who comprise a higher share of the US than the English population. This fits with prior research which showed dementia prevalence is higher for non-whites and Hispanics.[20,21] We did not observe higher dementia prevalence among Hispanics and non-whites in the US for those in relatively high socioeconomic groups.

For peer review only - http://bmjopen.bmj.com/site/about/guidelines.xhtml

Comparison with previous studies and how findings are an advance on current literature

Previous studies have shown cross-country variation in dementia prevalence. However, substantial heterogeneity in case-ascertainment methods across countries and studies makes interpreting any observed differences difficult. We believe this is the first study to compare dementia prevalence in England and the US using the exact same measure of dementia, thus overcoming previous difficulties in making comparisons across the two countries due differences in diagnostic practices and case definitions. We also compared the SES gradient of dementia in both countries. While some studies have shown in both England and the US those with lower education and less wealth have been found to have higher rates of dementia,[4,9,11,22,23] there are no systematic comparative studies. We compared prevalence along the SES gradient using almost identical measures of income, wealth, and education. Further, we standardized the cross-country comparison for age and gender, using the English over 70 population as the standard population. We found that dementia prevalence is higher and more concentrated among the poorest in the US than England. Detailed disaggregation according to SES measures shows the true extent of the excess burden of dementia in the very poorest group in the US.

We showed that risk factors for dementia such as cardiometabolic diseases, psychiatric conditions, high BMI, smoking have similar affects across countries. Accounting for these risk factors removes most of the SES gradient for both countries, but disproportionately high prevalence remains for the most disadvantaged in the US.

Implications (wider interpretation)

Much research has shown that low income Americans are more likely to be in poor health and die younger than their high-income counterparts.[24] We show that these health differences also extend to dementia prevalence.

While risk factors contribute to higher prevalence among those who are more disadvantaged, those in the US appear to have an undue burden that is caused by risk factors for which we cannot account. One possible explanation is differential access to healthcare. The NHS provides broadly equitable care according to education in the older population after accounting for health status.[25] In the US, the poor often go uninsured, and although virtually every American aged 65 or older is eligible for Medicare, around 20% of Medicare beneficiaries healthcare must be financed out of pocket.[26] The extent to which health care provision below and above aged 65 may account for the relative excess dementia burden in the US is unclear.

The implications of our results are that interventions designed to attempt to prevent dementia should be targeted towards the most disadvantaged. This is especially true in the US. As yet, we are unable to advocate specific measures as we do not yet understand the specific nature of disadvantage in respect to dementia risk.

Strengths and Weaknesses of Analysis

This study has a strong design. Results are directly comparable across England and the US. The same predictive algorithm was applied to both countries, addressing the problem of heterogeneity in case ascertainment which has affected the literature.[1,13] Further, because ELSA and HRS share sample

For peer review only - http://bmjopen.bmj.com/site/about/guidelines.xhtml

selection and questionnaire design, any differences in overall prevalence and SES gradients in prevalence across the two countries should represent true differences. In contrast to the Global Burden Disease study, we find higher dementia prevalence in the US. Furthermore, we measured the SES gradient of dementia across four different measures of SES, with consistent results. Our work also highlights the usefulness of the standardized measure of dementia to allow for meaningful comparisons across countries.

This study had three limitations. First, we do not ascertain dementia directly, but predict cases using a common battery of measures in ELSA and HRS. Importantly, Hurd's prediction algorithm has high accuracy and although our case definition lacks a clinical point of reference in England, it is based on a detailed clinical substudy in the US.[16] Further, cross-cultural subjectivities in reporting of impairment severity are likely to be similar in the US and England (see online appendix Table A5). It would be of great value for future work to use the Harmonized Cognitive Assessment Protocol (HCAP) data to provide a standard clinical point of reference to validate and verify the cross-country dementia prevalence estimates.[27] Second, education is one of the factors in the predictive algorithm for dementia and also one of our measures of SES. The dementia algorithm takes account of the well-documented correlation between education level and cognitive function in adult life. Nevertheless, we found substantial absolute and relative inequalities in dementia prevalence according to education level in the US and UK. Education may be a successful approach for reducing dementia risk.[28] Third, while we show risk factors explain a large proportion of the differences in dementia between the England and the US – although cannot account for the difference in the lowest income decile – there are likely other unmeasured confounding factors that impact dementia prevalence which we do not observe.

Conclusion

Given the large social and economic costs of dementia, there is great value in understanding the scope and burden of dementia in the population along the SES gradient. This study indicates that more disadvantaged individuals face a higher burden of dementia and that the poorest individuals in the US face a disproportionately high burden. The high burden faced by these individuals can be partly but not fully explained by past health factors. We lacked data on other important possible contributing factors such as habitual drug use. Further research is needed to fully understand this issue using data from multiple sources.

For peer review only

For peer review only - http://bmjopen.bmj.com/site/about/guidelines.xhtml

Table 1: Prevalence of Dementia, United States vs. England, 2017. Overall prevalence and prevalence according to four measures of socioeconomic status

	England		United States			
	Age-Gender Standardized Prevalence [95% CI]		Age-Gender Standardized Prevalence [95% CI]		Difference	p-value
All	0.097	[0.089 to 0.106]	0.112	[0.106 to 0.118]	0.014	0.0055
Household Income Decile						
1 (Lowest)	0.114	[0.089 to 0.139]	0.187	[0.166 to 0.208]	0.073	<0.0001
2	0.113	[0.090 to 0.136]	0.141	[0.119 to 0.163]	0.028	0.090
3	0.124	[0.097 to 0.151]	0.111	[0.095 to 0.127]	-0.013	0.42
4	0.099	[0.071 to 0.126]	0.118	[0.099 to 0.137]	0.019	0.26
5	0.094	[0.072 to 0.116]	0.086	[0.069 to 0.102]	-0.008	0.56
6	0.098	[0.070 to 0.127]	0.108	[0.088 to 0.128]	0.010	0.59
7	0.068	[0.042 to 0.093]	0.100	[0.078 to 0.122]	0.032	0.060
8	0.083	[0.053 to 0.114]	0.082	[0.066 to 0.097]	-0.002	0.92
9	0.082	[0.041 to 0.122]	0.093	[0.071 to 0.116]	0.012	0.62
10 (Highest)	0.059	[0.035 to 0.083]	0.077	[0.052 to 0.103]	0.019	0.30
Years of Schooling						
9 or fewer	0.128	[0.101 to 0.154]	0.190	[0.162 to 0.218]	0.062	0.0015
10	0.095	[0.074 to 0.116]	0.137	[0.109 to 0.165]	0.042	0.018
11	0.096	[0.077 to 0.115]	0.109	[0.080 to 0.139]	0.013	0.471
12	0.071	[0.042 to 0.100]	0.124	[0.114 to 0.133]	0.053	0.0006
13	0.061	[0.038 to 0.083]	0.116	[0.090 to 0.141]	0.055	0.0013
14 or more	0.056	[0.039 to 0.073]	0.085	[0.076 to 0.093]	0.029	0.0031
Household Wealth Decile						
1 (Lowest)	0.165	[0.132 to 0.198]	0.187	[0.162 to 0.211]	0.022	0.31
2	0.117	[0.092 to 0.143]	0.149	[0.129 to 0.169]	0.031	0.061
3	0.100	[0.073 to 0.127]	0.107	[0.091 to 0.122]	0.006	0.68
4	0.093	[0.071 to 0.115]	0.115	[0.098 to 0.132]	0.022	0.12
5	0.110	[0.085 to 0.134]	0.091	[0.077 to 0.106]	-0.018	0.21
6	0.080	[0.057 to 0.103]	0.089	[0.074 to 0.103]	0.008	0.55
7	0.070	[0.046 to 0.094]	0.103	[0.084 to 0.123]	0.034	0.034
8	0.092	[0.066 to 0.118]	0.089	[0.072 to 0.106]	-0.003	0.85
9	0.082	[0.048 to 0.116]	0.103	[0.084 to 0.123]	0.021	0.28
10 (Highest)	0.060	[0.038 to 0.081]	0.067	[0.051 to 0.084]	0.008	0.58
Household Non-Housing Wealth Decile						
1 (Lowest)	0.136	[0.103 to 0.168]	0.201	[0.176 to 0.226]	0.065	0.0019
2	0.109	[0.084 to 0.134]	0.137	[0.119 to 0.156]	0.029	0.074
3	0.123	[0.095 to 0.151]	0.131	[0.111 to 0.150]	0.008	0.66
4	0.096	[0.073 to 0.118]	0.101	[0.085 to 0.116]	0.005	0.71
5	0.108	[0.082 to 0.134]	0.107	[0.091 to 0.123]	-0.001	0.95
6	0.093	[0.067 to 0.119]	0.086	[0.070 to 0.101]	-0.007	0.64
7	0.099	[0.072 to 0.125]	0.086	[0.070 to 0.102]	-0.013	0.42
8	0.078	[0.051 to 0.105]	0.090	[0.073 to 0.106]	0.011	0.49
9	0.058	[0.036 to 0.081]	0.092	[0.074 to 0.111]	0.034	0.024
10 (Highest)	0.063	[0.043 to 0.084]	0.079	[0.062 to 0.095]	0.015	0.26

Notes: Sample includes non-Hispanic white population aged 70+ only. The sample size is 3,147 participants in England and 5,330 participants in the United States. All estimates are age-gender standardized to the overall 2016 aged 70+ white population in England. The difference is calculated as the prevalence in the US minus prevalence in England. All deciles are calculated within country.

For peer review only - http://bmjopen.bmj.com/site/about/guidelines.xhtml

Figure 1: SES Gradient of Dementia, United States vs England, 2017, according to four measures of socioeconomic status. Absolute and relative inequality shown with 95% confidence intervals.

[Figure 1 Attached separately]

Notes: The points in this figure represent the mean age-gender standardized dementia prevalence for each country by socioeconomic status (SES), along with 95% confidence interval for these predictions. The solid lines represent the fitted Slope Index of Inequality (SII: absolute inequality) for each country. The values of the SII and the corresponding Relative Index of Inequality (RII: relative inequality) are listed in the top right of each figure, with 95% Confidence Intervals in brackets. For education, individuals are ranked based on their years of schooling within each country, and as the US has higher educational attainment, individuals with 14+ years of schooling are the 80th percentile of the US education distribution, but at the 90th percentile of the English educational distribution.

Table 2: Difference in Prevalence of Dementia, United States vs England, 2017. Estimates of the between-country difference taking account of antecedent health status, smoking and degree of adiposity

Whole Sample:					
Percentage Point Difference	1.43	1.34	1.15	1.21	1.18
p-value	0.0055	0.0091	0.025	0.020	0.034
% Difference from Baseline	--	-6%	-19%	-16%	-17%
Excluding Lowest Income Decile:					
Percentage Point Difference	0.89	0.88	0.74	0.75	0.81
p-value	0.11	0.11	0.18	0.18	0.18
% Difference from Baseline	--	-2%	-17%	-16%	-10%
Lowest Income Decile:					
Percentage Point Difference	7.27	6.14	5.45	5.93	4.85
p-value	<0.0001	0.0003	0.002	0.0011	0.012
% Difference from Baseline	--	-15%	-25%	-18%	-33%
Control for:					
Past Cardiometabolic Diseases		✓	✓	✓	✓
Past Psychiatric Conditions			✓	✓	✓
Ever Smoked				✓	✓
Past BMI					✓

Notes: Sample includes non-Hispanic white population aged 70+ only. The sample size is 3,147 participants in England and 5,330 participants in the United States. All estimates are age-gender standardized to the overall 2016 aged 70+ white population in England. The difference is calculated as the prevalence in the US minus prevalence in England. Differences are displayed as percentage points. 'Past Cardiometabolic Diseases' and 'Past Psychiatric Conditions' control for whether an individual says they had the conditions 4 years prior. 'Cardiometabolic Diseases' include diabetes, heart disease, and stroke. 'Smoking' controls for whether an individual has ever smoked. BMI stands for Body Mass Index. 'Past BMI' includes dummy variables to control for whether an individual is classed as underweight, overweight, or obese. BMI values are based on when an individual first entered the survey, which is at least 10 years prior.

For peer review only - http://bmjopen.bmj.com/site/about/guidelines.xhtml

For peer review only

Contributors and Transparency Statement

All authors made a substantial contribution to study conception and design. EF and JM developed the original idea. JM and KA prepared and analysed the data, with inputs from EF and EJB. All authors contributed to interpreting the results and drafting the manuscript. JM and KA serve as guarantors and affirm that the manuscript is an honest, accurate, and transparent account of the study being reported; that no important aspects of the study have been omitted; and that any discrepancies from the study as planned have been explained. All authors had full access to all of the data (including statistical reports and tables) in the study and take responsibility for the integrity of the data and accuracy of the data analysis. The corresponding author (JM) attests that all listed authors meet authorship criteria and that no others meeting the criteria have been omitted. Author names on manuscript are listed in alphabetical order.

Competing interests

All authors have completed the ICMJE uniform disclosure form and declare no support from any organisation for the submitted work; no financial relationships with any organisations that might have an interest in the submitted work in the previous three years; no other relationships or activities that could appear to have influenced the submitted work

Acknowledgments

We thank Paco Martorell and Ken Langa for help with understanding the prediction equations and Liliana French for editorial assistance.

Ethical approval

The HRS has ethical approval from the University of Michigan Institutional Review Board. ELSA has ethical approval from the London Multicentre Research Ethics Committee (MREC/01/2/91). This study did not require additional ethical approval as we conduct secondary analysis of these two publicly available datasets.

Funding

This work was supported by grants from the British Heart Foundation (RG/13/2/30098, RG/16/11/32334), MRRC, ESRC, and Onassis Foundation.

Data sharing

Appendix with details on the formula and calculations is available online. Code and results are available on request from the corresponding author.

For peer review only

References

1 Nichols E, Szoeke CE, Vollset SE, *et al.* Global, regional, and national burden of Alzheimer's disease and other dementias, 1990–2016: a systematic analysis for the Global Burden of Disease Study 2016. *Lancet Neurol* 2019;**18**:88–106.

2 Kuo C-L, Pilling LC, Atkins JL, *et al.* ApoE e4e4 genotype and mortality with COVID-19 in UK Biobank. *medRxiv* 2020.

3 Hurd MD, Martorell P, Delavande A, *et al.* Monetary costs of dementia in the United States. *N Engl J Med* 2013;**368**:1326–34.

4 Hudomiet P, Hurd MD, Rohwedder S. The relationship between lifetime out-of-pocket medical expenditures, dementia, and socioeconomic status in the US. *J Econ Ageing* 2019;**14**:100181.

5 Banks J, Marmot M, Oldfield Z, *et al.* Disease and disadvantage in the United States and in England. *Jama* 2006;**295**:2037–45.

6 Marmot MG. Status syndrome: a challenge to medicine. *Jama* 2006;**295**:1304–7.

7 Brunner EJ. Social factors and cardiovascular morbidity. *Neurosci Biobehav Rev* 2017;**74**:260–8.

8 Marmot M. Health equity in England: the Marmot review 10 years on. *Bmj* 2020;**368**.

9 Rusmaully J, Dugravot A, Moatti J-P, *et al.* Contribution of cognitive performance and cognitive decline to associations between socioeconomic factors and dementia: A cohort study. *PLoS Med* 2017;**14**:e1002334.

10 George KM, Lutsey PL, Kucharska-Newton A, *et al.* Life Course Individual and Neighborhood Socioeconomic Status and Risk of Dementia in the Atherosclerosis Risk in Communities Neurocognitive Study (ARIC-NCS). *Am J Epidemiol* 2020.

11 Cadar D, Lassale C, Davies H, *et al.* Individual and area-based socioeconomic factors associated with dementia incidence in England: evidence from a 12-year follow-up in the English longitudinal study of ageing. *JAMA Psychiatry* 2018;**75**:723–32.

12 Global Burden of Disease. Global Burden of Disease. GBD Compare. IHME Viz Hub. GBD Comp. Viz Hub. https://vizhub.healthdata.org/gbd-compare/ (accessed 18 Jul 2020).

13 Launer LJ. Statistics on the burden of dementia: need for stronger data. *Lancet Neurol* 2019;**18**:25–7.

14 Sonnega A, Faul JD, Ofstedal MB, *et al.* Cohort profile: the health and retirement study (HRS). *Int J Epidemiol* 2014;**43**:576–85.

15 Steptoe A, Breeze E, Banks J, *et al.* Cohort profile: the English longitudinal study of ageing. *Int J Epidemiol* 2013;**42**:1640–8.

16 Gianattasio KZ, Wu Q, Glymour MM, *et al.* Comparison of methods for algorithmic classification of dementia status in the health and retirement study. *Epidemiol Camb Mass* 2019;**30**:291.

17 Jorm AF, Christensen H, Korten A, *et al.* Informant ratings of cognitive decline in old age: validation against change on cognitive tests over 7 to 8 years. *Psychol Med* 2000;**30**:981–5.

18 Banks J, Muriel A, Smith JP. Attrition and health in ageing studies: evidence from ELSA and HRS. *Longitud Life Course Stud* 2011;**2**:101–26.

19 Wagstaff A, Paci P, Van Doorslaer E. On the measurement of inequalities in health. *Soc Sci Med* 1991;**33**:545–57.

For peer review only

For peer review only - http://bmjopen.bmj.com/site/about/guidelines.xhtml

20 Yaffe K, Falvey C, Harris TB, *et al.* Effect of socioeconomic disparities on incidence of dementia among biracial older adults: prospective study. *Bmj* 2013;**347**.

21 Vega IE, Cabrera LY, Wygant CM, *et al.* Alzheimer's disease in the latino community: Intersection of genetics and social determinants of health. *J Alzheimers Dis* 2017;**58**:979–92.

22 Langa KM, Larson EB, Crimmins EM, *et al.* A comparison of the prevalence of dementia in the United States in 2000 and 2012. *JAMA Intern Med* 2017;**177**:51–8.

23 Rocca WA, Petersen RC, Knopman DS, *et al.* Trends in the incidence and prevalence of Alzheimer's disease, dementia, and cognitive impairment in the United States. *Alzheimers Dement* 2011;**7**:80–93.

24 Chetty R, Stepner M, Abraham S, *et al.* The association between income and life expectancy in the United States, 2001-2014. *Jama* 2016;**315**:1750–66.

25 Stoye G, Zaranko B, Shipley M, *et al.* Educational inequalities in hospital use among the over 65s in England. *Milbank Q* 2020.

26 De Nardi M, French E, Jones JB, *et al.* Medical spending of the US elderly. *Fisc Stud* 2016;**37**:717–47.

27 Langa KM, Ryan LH, McCammon RJ, *et al.* The health and retirement study harmonized cognitive assessment protocol project: study design and methods. *Neuroepidemiology* 2020;**54**:64–74.

28 Nguyen TT, Tchetgen EJT, Kawachi I, *et al.* Instrumental variable approaches to identifying the causal effect of educational attainment on dementia risk. *Ann Epidemiol* 2016;**26**:71–6.

For peer review only

For peer review only - http://bmjopen.bmj.com/site/about/guidelines.xhtml

Author Licence

I *Jeremy McCauley [*The Corresponding Author] of this article contained within the original manuscript which includes any diagrams & photographs within and any related or stand alone film submitted (the Contribution") has the right to grant on behalf of all authors and does grant on behalf of all authors, a licence to the BMJ Publishing Group Ltd and its licencees, to permit this Contribution (if accepted) to be published in the BMJ and any other BMJ Group products and to exploit all subsidiary rights, as set out in our licence set out at: http://www.bmj.com/about-bmj/resources-authors/forms-policies-and-checklists/copyright-open-access-and-permission-reuse."

IF YOU ARE A NATIONAL INSTITUTE OF HEALTH ("NIH") EMPLOYEE, CONTRACTOR OR TRAINEE the following cover sheet will be accepted by the BMJ Group and NIH and incorporated into the above Licence.

Please tick **one or more** boxes as appropriate:

- ☐ I am the sole author of the Contribution.
- ✓ I am one author signing on behalf of all co-owners of the Contribution.
- ☐ The Contribution has been made in the course of my employment and I am signing as authorised by my employer.
- ☐ I am a US Federal Government employee acting in the course of my employment.
- ☐ I am not a US Federal Government employee, but some or all of my co-authors are.
- ☐ I am an employee of the UK Crown* acting in the course of my employment
- ☐ I am a US Federal Government employee acting in the course of my employment.
- ☐ I am not a US Federal Government employee, but some or all of my co-authors are.
- ☐ I am an employee of the UK Crown acting in the course of my employment
- ☐ I am not an employee of the UK Crown acting in the course of my employment but some/all of my co-authors are.*

For peer review only

For peer review only - http://bmjopen.bmj.com/site/about/guidelines.xhtml

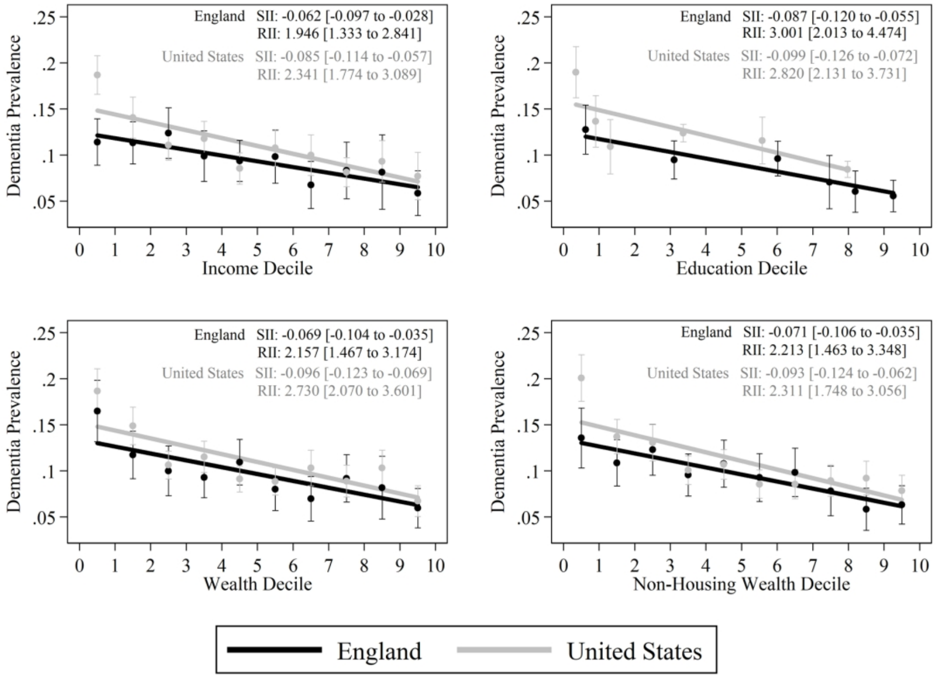

SES Gradient of Dementia, United States vs England, 2017, according to four measures of socioeconomic status. Absolute and relative inequality shown with 95% confidence intervals.

169x123mm (300 x 300 DPI)

Supplementary Appendix (For Online Publication)

Research in context

We reviewed existing evidence in July 2020, searching PubMed database for any studies looking at cross-country comparisons of dementia using the same standard measures and diagnostic practices.

("Dementia"[mesh]) AND ("Prevalence"[mesh])

AND

(“Survey”[tw]) AND (“Population”[tw]) AND (“Representative”[tw])

AND

(“Cross-Country”[ti] OR “Comparison”[ti])

This search yielded no results. The only relevant project we could find relating to our research was regarding the Health and Retirement Study Harmonized Cognitive Assessment Protocol (HCAP) Project. [1] This is an international research collaboration funded by the National Institute on Aging to better measure and identify cognitive impairment and dementia in longitudinal studies of countries around the world. However, this project has not yet released any international comparisons.

We also reviewed existing evidence in the PubMed database for any studies looking at how dementia prevalence varies by socioeconomic status (SES) in either England or the US. The search terms used were the following:

("Dementia"[mesh]) AND

("Prevalence"[tw]) AND ("Socioeconomic"[ti] OR "Socioeconomic"[tw] OR "SES"[tw]) AND

(“United Kingdom”[mesh] OR “United Kingdom”[tw] OR “England”[tw] OR “UK”[tw] OR “United Kingdom”[tw] "Britain"[tw] OR “United States”[mesh] OR “United States”[tw] OR “US”[tw] OR “America”[tw])

This search yielded 50 results. Papers which were not relevant were manually removed. We also excluded papers that did not use nationally representative samples. We performed additional searches using lists of references retrieved from relevant papers. Relevant papers found in the search can be found in Table A1 below.

For peer review only - http://bmjopen.bmj.com/site/about/guidelines.xhtml

Table A1: Studies on Dementia Prevalence by Socioeconomic Status (SES)

Authors	Country	Socioeconomic Indicator	Years	Findings
Langa et al. (2017)[1]	United States	Education	2000 and 2012	Link between education and dementia risk
Hudomiet et al, (2019)[2]	United States	Education and Social Security Benefits	1998 to 2014	Link between education and dementia risk
Rusmaully et al, (2017)[3]	United Kingdom	Education, height, occupational position	Various years between 1985 and 2015	High cognitive reserve associated with lower risk for dementia
Cadar et al, (2018)[4]	United Kingdom	Education, wealth and the index of multiple deprivation	2002 to 2015	Lower wealth in but not education associated with increased risk for dementia
Rocca et al,(2011)[5]	United States	Education, net worth	1993 and 2002	Higher education, higher net worth protected against cognitive impairment.
Basu (2013)[6]	United States	Education	2000 - 2002	Education has causal effect on dementia risk
Nguyen et al, (2016)[7]	United States	Education	1998 to 2010	Education protective against dementia risk
Crimmins et al, (2018)[8]	United States	Education	2000 and 2010	More education linked to lower dementia prevalence
Garcia et al, (2018)[9]	United States	Race, ethnicity, nativity, and education	2012	Education reduces the odds for CIND
Weden et al, (2018)[10]	United States	Race, ethnicity, total number of children, marital status, highest educational attainment, and net total assets in 2000	2000 and 2010	Strong protective role of educational attainment and persisting rural disadvantages for dementia

For peer review only

For peer review only - http://bmjopen.bmj.com/site/about/guidelines.xhtml

Data Appendix

HRS Data

The Health and Retirement Study (HRS) is a nationally representative, biennial longitudinal survey of adults in the United States.[11] It started in 1992 and since it collects a wide range of questions on income, wealth, employment, health, cognition, and demographics. It utilises a steady-state sampling design, with a new cohort aged 51-56 entering every 6 years. In total, 43,478 individuals have been interviewed to date. We used data from the 2016 and earlier waves to predict dementia and focused on the over the age of 70 years old that live in the community or in nursing homes in 2016. This left a sample of 7,165 individuals. We restricted our attention to non-Hispanic whites for comparability with ELSA. These restrictions generated a main study sample of 5,330 participants with 4,932 being self-respondents, and 398 proxy interviews.

ELSA Data

The English Longitudinal Study of Ageing (ELSA) is a biennial longitudinal survey of adults in England, developed as a companion study to the HRS.[12] ELSA was also designed to be nationally representative of the non-institutionalised population. Respondents remain the study if they become institutionalised. While it is has been shown to be representative of the English population in terms of sociodemographic characteristics, the proportion of non-white people in the survey is very small.[12] We used data from the 2016 and earlier waves and focused on the aged over 70 years old that live in the community or in nursing homes in 2016. This left a sample of 3,224 individuals. We restricted our attention to non-Hispanic whites for comparability. These restrictions generated a study sample of 3,147 participants with 3,007 being self-respondents, and 140 proxy interviews.

Harmonised Variables HRS and ELSA

We use the same set of cognitive and demographic variables available in both the HRS and ELSA.

For cognitive and demographic measures, we used the same questions as Hurd et al.[13] Specifically, we used variables on demographics, difficulties respondents have with Activities of Daily Living (ADLs), Instrumental Activities of Daily Living (IADLs), and a range of cognitive function tests adapted from the Telephone Interview for Cognitive Status (TICS) and their change across waves. For Proxy respondents we also used the shortened 16 question form of IQCODE.

We used four variables to measure socioeconomic status (SES). These are household income, education, total household wealth, and total household non-housing wealth. For the HRS we used variables from HRS RAND dataset. To establish each respondent's SES in terms of income, wealth, and non-housing wealth, we assigned everyone to a decile rank for each variable in their respective country. For education, we ranked respondents according to their number of years of schooling. We used respondents' reported wealth and non-housing wealth from 4 years prior. If a respondent had a missing value of income, wealth, or non-housing wealth we used the most recent non-missing observation from previous waves. All amounts of income, wealth and non-housing wealth were deflated to 2016 GBPs, and USD amounts were converted to GBPs using 1 USD = 0.75 GBP.

Finally, in our analysis, we investigated how variables that have been associated with dementia affect our results. These include Body Mass Index (BMI), past smoking behaviour, past stroke. Respondents are asked whether they have any health conditions, of which we use whether they had any cardiometabolic diseases (diabetes, heart disease, and stroke) and/or psychiatric conditions 4 years prior. BMI is collected by a nurse for every ELSA respondent when they first enter the survey, which for everyone over 70 in the 2016 survey means their BMI was collected over 10 years prior. BMI is recorded in each wave of HRS based on self-reported height and weight. To make the BMI measures in HRS comparable with ELSA, we used respondents first ever recorded BMI in HRS, which for our sample was at least 10 years prior.

Hurd et al. Dementia Prediction Algorithm

To estimate the probability of dementia for each individual present in our pooled data we follow the method of Hurd et al. that is based on the HRS supplement Aging, Demographics and Memory Study (ADAMS).[13] It is a representative subsample of HRS members aged over 70 that received a detailed in-home assessment of their cognitive status by experienced teams at the Duke University Dementia Epidemiology

For peer review only - http://bmjopen.bmj.com/site/about/guidelines.xhtml

Research Center.[14] Consensus conferences were used to establish a final diagnosis of dementia or a cognitive impairment with no dementia (CIND) for each participant. Hurd et al. applied separate ordered probit models to self- and proxy-respondents to generate a predictive algorithm, based on the ADAMS diagnoses, for the whole HRS sample, using a range of variables including demographic information, ADLs, IADLs, the TICS questionnaire, IQCODE, lagged variables and differences in variables between waves. The algorithm predicts the probability of dementia in the following year.

Most of the coefficients used in the predictive algorithm can be found in the online appendix of the Hurd et al. paper. To account for the small number of missing variables in HRS and ELSA we either used coefficients provided by the authors or estimated them by a conditional minimum distance estimator using the publicly available HRS data and the predicted values of the researcher contribution Dementia Predicted Probability Files (DPPF), that follows the method of Hurd et al. To verify our predictions in HRS, we compared our predictions with the DPPF data available on the HRS website for the year 2008 and found that we accurately matched predicted dementia.

Hurd et al’s predictive algorithm has been shown to have high specificity and accuracy, and generally performs well in terms of sensitivity compared to other predictive algorithms for dementia.[15]

For peer review only

Table A2: HRS and ELSA Response Rates

Year	2010	2012	2014	2016
HRS				
Wave	10	11	12	13
Response rate (%)	81.0	89.1	87.1	84.3
ELSA				
Wave	5	6	7	8
Fieldwork response rate (%)	79.1	80.2	80.1	82.4

Notes: The response rates are calculated differently in HRS and ELSA. For the HRS, the response rate includes all individuals who were determined to be eligible for HRS who completed a baseline interview.[16] For ELSA, the fieldwork response rate is the proportion of eligible survey units who participate in the research study, where 'eligible' means not having been found to be ineligible through death or moving out of Great Britain.[17]

For peer review only

Table A3: Summary Statistics, Raw Sample, England vs United States

	Raw Full Sample		With Population Weights	
	Non-Standardized Mean		Non-Standardized Mean	
	England	United States	England	United States
Total Sample Size	3,147	5,330	3,147	5,330
Proxy Respondents (%)	0.044	0.075	0.050	0.072
Age	78.8	80.5	79.5	79.7
Female	0.551	0.590	0.553	0.561
Married	0.580	0.517	0.584	0.536
Current Income and Wealth (£)				
Income	21,070	28,800	20,215	30,240
Wealth	280,000	210,000	270,000	230,000
Non-Housing Wealth	40,000	88,275	36,000	96,150
Wealth 4 Years Prior (£)				
Wealth	270,000	220,000	250,000	230,000
Non-Housing Wealth	42,300	96,800	35,727	100,000
Education				
Less than High School	0.362	0.174	0.424	0.161
High-school or Some College	0.473	0.573	0.444	0.560
College	0.165	0.253	0.132	0.279
Current Health				
ADLs (Out of 6)	0.510	0.583	0.561	0.548
IADLs (Out of 5)	0.348	0.513	0.411	0.482
Arthritis	0.569	0.728	0.578	0.715
Cancer	0.190	0.251	0.189	0.244
Lung disease	0.117	0.135	0.118	0.127
Diabetes	0.162	0.246	0.166	0.240
Heart Disease	0.390	0.383	0.396	0.365
High Blood Pressure	0.576	0.684	0.583	0.671
Psychiatric Condition	0.134	0.171	0.132	0.175
Stroke	0.092	0.103	0.096	0.095
Health 4 Years Prior				
ADLs	0.346	0.274	0.381	0.267
IADLs	0.159	0.228	0.185	0.219
Diabetes	0.131	0.215	0.135	0.212
Heart Disease	0.309	0.316	0.312	0.303
High Blood Pressure	0.513	0.653	0.519	0.643
Psychiatric Condition	0.120	0.157	0.118	0.162
Stroke	0.063	0.074	0.065	0.067
Other Health Factors				
Ever Smoked	0.660	0.553	0.674	0.561
BMI: <20 (Underweight)	0.016	0.038	0.015	0.036
BMI: 20-24.9 (Normal Weight)	0.266	0.352	0.262	0.343
BMI: 25-29.9 (Overweight)	0.461	0.405	0.461	0.410
BMI: 30+ (Obese)	0.256	0.205	0.262	0.211

Notes: Sample includes non-Hispanic white population aged 70+ only. The sample size is participants in England and participants in the United States. The mean is presented for each variable, apart from income and wealth variables where medians values are displayed. All amounts deflated to 2016 £s. Dollar amounts converted to £ using 1 $ = 0.75 £. Age is top coded at 99.

For peer review only

Table A4: Summary Statistics, Age-Gender Standardized, England vs United States

	Full Sample		Lowest Income Decile	
	Age-Gender Standardized Mean		Age-Gender Standardized Mean	
	England	United States	England	United States
Age	79.5	79.3	79.7	79.7
Female	0.553	0.553	0.553	0.553
Married	0.584	0.547	0.148	0.170
Current Income and Wealth (£)				
Income	20,547	31,927	8,247	8,645
Wealth	271,511	236,431	120,261	46,762
Non-Housing Wealth	38,715	100,996	7,778	5,509
Wealth 4 Years Prior (£)				
Wealth	257,459	242,655	111,830	55,953
Non-Housing Wealth	40,604	110,530	6,476	13,724
Education				
Less than High School	0.424	0.160	0.619	0.323
High-school or Some College	0.444	0.559	0.318	0.556
College	0.132	0.281	0.063	0.122
Current Health				
ADLs (Out of 6)	0.561	0.523	0.559	0.827
IADLs (Out of 5)	0.411	0.457	0.421	0.679
Arthritis	0.578	0.715	0.617	0.750
Cancer	0.189	0.244	0.157	0.196
Lung disease	0.118	0.127	0.137	0.217
Diabetes	0.166	0.242	0.157	0.283
Heart Disease	0.396	0.364	0.402	0.427
High Blood Pressure	0.583	0.671	0.619	0.703
Psychiatric Condition	0.132	0.174	0.158	0.262
Stroke	0.096	0.094	0.111	0.135
Health 4 Years Prior				
ADLs	0.381	0.255	0.424	0.546
IADLs	0.185	0.205	0.218	0.393
Diabetes	0.135	0.214	0.131	0.254
Heart Disease	0.312	0.302	0.345	0.352
High Blood Pressure	0.519	0.640	0.559	0.662
Psychiatric Condition	0.118	0.162	0.142	0.271
Stroke	0.065	0.066	0.064	0.099
Other Health Factors				
Ever Smoked	0.674	0.565	0.741	0.594
BMI: <20 (Underweight)	0.015	0.036	0.025	0.032
BMI: 20-24.9 (Normal Weight)	0.261	0.342	0.361	0.364
BMI: 25-29.9 (Overweight)	0.461	0.410	0.397	0.339
BMI: 30+ (Obese)	0.262	0.212	0.216	0.265

Notes: Sample includes non-Hispanic white population aged 70+ only. The sample size is 3,147 participants in England and 5,330 participants in the United States. The age-gender standardized mean is presented for each variable, apart from income and wealth variables where age-gender standardised medians values are displayed. The standard population is the overall age 70+ population in England in 2016. All amounts deflated to 2016 £s. Dollar amounts converted to £ using 1 $ = 0.75 £. Age is top coded at 99.

For peer review only

Table A5: Summary Statistics for Predictor Variables, England vs United States				
	Full Sample		Lowest Income Decile	
	Age-Gender- Standardized Mean		Age-Gender-Standardized Mean	
	England	United States	England	United States
**Cognitive Scores**				
Dates (Out of 4)	3.663	3.387	3.573	3.198
Backward counting 20	0.912	0.875	0.935	0.830
Serial 7 (Out of 5)	3.787	3.364	3.667	2.580
Scissor	0.978	0.920	0.993	0.912
Cactus	0.915	0.891	0.913	0.842
PM/Vice-President	0.846	0.900	0.828	0.854
Immediate recall (Out of 10)	5.318	4.631	5.010	3.972
Delayed recall (Out of 10)	3.797	3.684	3.185	2.894
**Limitations**				
ADLs (Out of 6)	0.561	0.523	0.559	0.827
IADLs (Out of 5)	0.411	0.457	0.421	0.679
Proxy Respondent	0.050	0.068	0.038	0.075

Notes: Sample includes non-Hispanic white population aged 70+ only. The sample size is 3,147 participants in England and 5,330 participants in the United States. The Cognitive Scores are from a range of tests adapted from Telephone Interview for Cognitive Status (TICS). Only non-proxy respondents answered the TICS questions. ADL and IADLs are Activities of Daily Living and Instrumental Activities of Daily Living, respectively.

For peer review only

Table A6: Indexes of Inequality

	Slope Index of Inequality (SII)			Relative Index of Inequality (RII)		
	England	United States	Difference	England	United States	Difference
Income	-0.062	-0.085	-0.023	1.946	2.341	1.203
	[-0.097 to -0.028]	[-0.114 to -0.057]	(0.31)	[1.333 to 2.841]	[1.774 to 3.089]	(0.44)
Education	-0.087	-0.099	-0.011	3.001	2.820	0.940
	[-0.120 to -0.055]	[-0.126 to -0.072]	(0.56)	[2.013 to 4.474]	[2.131 to 3.731]	(0.80)
Wealth	-0.069	-0.096	-0.026	2.157	2.730	1.265
	[-0.104 to -0.035]	[-0.123 to -0.069]	(0.24)	[1.467 to 3.174]	[2.070 to 3.601]	(0.33)
NH Wealth	-0.071	-0.093	-0.022	2.213	2.311	1.044
	[-0.106 to -0.035]	[-0.124 to -0.062]	(0.36)	[1.463 to 3.348]	[1.748 to 3.056]	(0.87)
**Excluding Most Disadvantaged Group**						
Income	-0.067	-0.060	0.007	2.104	1.833	0.871
	[-0.107 to -0.027]	[-0.093 to -0.027]	(0.79)	[1.343 to 3.296]	[1.313 to 2.560]	(0.63)
Education	-0.070	-0.062	0.008	2.509	1.932	0.770
	[-0.107 to -0.032]	[-0.094 to -0.030]	(0.76)	[1.548 to 4.065]	[1.378 to 2.708]	(0.39)
Wealth	-0.065	-0.059	0.005	2.092	1.887	0.902
	[-0.104 to -0.025]	[-0.091 to -0.028]	(0.84)	[1.319 to 3.317]	[1.349 to 2.639]	(0.72)
NH Wealth	-0.064	-0.075	-0.012	2.077	1.981	0.954
	[-0.107 to -0.020]	[-0.110 to -0.040]	(0.69)	[1.217 to 3.544]	[1.433 to 2.740]	(0.88)
**Full Sample with Controls**						
Income	-0.040	-0.051	-0.011	1.499	1.698	1.133
	[-0.075 to -0.004]	[-0.079 to -0.023]	(0.63)	[1.028 to 2.187]	[1.275 to 2.263]	(0.61)
Education	-0.0336	-0.056	-0.022	1.308	1.742	1.332
	[-0.0754 to 0.008]	[-0.086 to -0.026]	(0.40)	[0.899 to 1.903]	[1.306 to 2.324]	(0.24)
Wealth	-0.060	-0.045	0.015	1.777	1.483	0.834
	[-0.097 to -0.023]	[-0.074 to -0.015]	(0.53)	[1.224 to 2.582]	[1.131 to 1.945]	(0.44)
NH Wealth	-0.050	-0.051	-0.002	1.562	1.614	1.033
	[-0.087 to -0.012]	[-0.080 to -0.023]	(0.94)	[1.078 to 2.262]	[1.231 to 2.116]	(0.89)

Notes: This table shows the Slope Index of Inequality (SII) and Relative Index of Inequality (RII), calculated using generalized linear models (log binomial regression) with identity and logarithmic link functions, respectively. The SIIs are displayed graphically in Figure 1. The estimates of RII are risk ratios. P-values are displayed in parentheses under differences. The panel 'Excluding Most Disadvantaged Group' excludes individuals in the bottom income decile. The panel 'Full Sample with Controls' controls for whether an individual had any cardiometabolic diseases (diabetes, heart disease, and stroke) and/or psychiatric conditions 4 years prior, whether an individual has ever smoked, and Body Mass Index (BMI) from at least 10 years prior.

For peer review only

Table A7: Difference in Prevalence of Dementia with Controls, United States vs England, 2017

Whole Sample:					
England Prevalence	0.097	0.088	0.086	0.089	0.092
	(0.089 to 0.106)	(0.078 to 0.098)	(0.073 to 0.099)	(0.072 to 0.106)	(0.072 to 0.111)
United States	0.112	0.100	0.098	0.101	0.120
	(0.106 to 0.118)	(0.092 to 0.108)	(0.087 to 0.109)	(0.087 to 0.114)	(0.104 to 0.135)
Percentage Point Difference	1.43	1.15	1.21	1.18	2.82
p-value	0.0055	0.025	0.020	0.034	<0.0001
Excluding Lowest Income Decile:					
England Prevalence	0.094	0.086	0.086	0.090	0.094
	(0.085 to 0.103)	(0.076 to 0.096)	(0.073 to 0.1)	(0.073 to 0.108)	(0.074 to 0.114)
United States	0.103	0.093	0.094	0.098	0.115
	(0.097 to 0.109)	(0.085 to 0.102)	(0.083 to 0.105)	(0.084 to 0.113)	(0.099 to 0.131)
Percentage Point Difference	0.89	0.74	0.75	0.81	2.09
p-value	0.11	0.18	0.18	0.18	0.0021
Lowest Income Decile:					
England Prevalence	0.114	0.107	0.088	0.082	0.095
	(0.089 to 0.139)	(0.074 to 0.14)	(0.036 to 0.14)	(0.021 to 0.144)	(0.025 to 0.164)
United States	0.187	0.162	0.148	0.131	0.163
	(0.166 to 0.208)	(0.129 to 0.195)	(0.103 to 0.192)	(0.078 to 0.184)	(0.103 to 0.223)
Percentage Point Difference	7.27	5.45	5.93	4.85	6.85
p-value	<0.0001	0.0020	0.0011	0.012	0.0015
Control for:					
Past Health Conditions		✓	✓	✓	✓
Ever Smoked			✓	✓	✓
Past BMI				✓	✓
Education					✓

Notes: Sample includes non-Hispanic white population aged 70+ only. The sample size is 3,147 participants in England and 5,330 participants in the United States. All estimates are age-gender standardized to the overall 2016 aged 70+ white population in England. The difference is calculated as the prevalence in the US minus prevalence in England. Differences are displayed as percentage points. 'Past Health Conditions' controls for whether an individual had any cardiometabolic diseases (diabetes, heart disease, and stroke) and/or psychiatric conditions 4 years prior. 'Ever Smoked' controls for whether an individual has ever smoked. BMI stands for Body Mass Index. 'Past BMI' includes dummy variables to control for whether an individual is classed as underweight, normal weight, *overweight* or *obese*. BMI measurements are based on when an individual first entered the survey, which is at least 10 years prior. We assume the effect of each control is constant across countries and age/gender groups. The reference groups are: No past health conditions; never smoked; normal weight; and high-school education.

For peer review only

Table A8: Difference in Prevalence of Dementia with Controls, United States vs England, 2017

	England			United States		
Whole Sample:						
Prevalence	0.097	0.092	0.089	0.112	0.105	0.101
CI	(0.089 to 0.106)	(0.082 to 0.102)	(0.072 to 0.106)	(0.106 to 0.118)	(0.098 to 0.113)	(0.087 to 0.114)
Income Decile:						
1 *Prevalence*	0.114	0.112	0.082	0.187	0.173	0.131
CI	(0.089 to 0.139)	(0.079 to 0.144)	(0.021 to 0.144)	(0.166 to 0.208)	(0.142 to 0.205)	(0.078 to 0.184)
2 *Prevalence*	0.113	0.119	0.118	0.141	0.145	0.148
CI	(0.09 to 0.136)	(0.088 to 0.149)	(0.05 to 0.187)	(0.119 to 0.163)	(0.113 to 0.176)	(0.086 to 0.21)
3 *Prevalence*	0.124	0.107	0.130	0.111	0.089	0.093
CI	(0.097 to 0.151)	(0.074 to 0.139)	(0.073 to 0.187)	(0.095 to 0.127)	(0.067 to 0.11)	(0.048 to 0.137)
4 *Prevalence*	0.099	0.095	0.094	0.118	0.114	0.111
CI	(0.071 to 0.126)	(0.065 to 0.126)	(0.041 to 0.148)	(0.099 to 0.137)	(0.088 to 0.14)	(0.062 to 0.16)
5 *Prevalence*	0.094	0.087	0.061	0.086	0.084	0.063
CI	(0.072 to 0.116)	(0.062 to 0.112)	(0.024 to 0.098)	(0.069 to 0.102)	(0.063 to 0.104)	(0.035 to 0.09)
6 *Prevalence*	0.098	0.097	0.116	0.108	0.107	0.100
CI	(0.07 to 0.127)	(0.064 to 0.131)	(0.056 to 0.177)	(0.088 to 0.128)	(0.081 to 0.134)	(0.053 to 0.148)
7 *Prevalence*	0.068	0.066	0.073	0.100	0.101	0.104
CI	(0.042 to 0.093)	(0.038 to 0.094)	(0.021 to 0.124)	(0.078 to 0.122)	(0.073 to 0.129)	(0.054 to 0.154)
8 *Prevalence*	0.083	0.084	0.097	0.082	0.078	0.085
CI	(0.053 to 0.114)	(0.051 to 0.117)	(0.054 to 0.14)	(0.066 to 0.097)	(0.058 to 0.098)	(0.054 to 0.117)
9 *Prevalence*	0.082	0.076	0.100	0.093	0.085	0.107
CI	(0.041 to 0.122)	(0.034 to 0.118)	(0.048 to 0.151)	(0.071 to 0.116)	(0.06 to 0.111)	(0.07 to 0.145)
10 *Prevalence*	0.059	0.058	0.051	0.077	0.077	0.068
CI	(0.034 to 0.083)	(0.029 to 0.087)	(0.015 to 0.087)	(0.052 to 0.103)	(0.051 to 0.103)	(0.036 to 0.1)
Control for:						
Past Health Conditions		✓	✓		✓	✓
Ever Smoked			✓			✓
Past BMI			✓			✓

Notes: Sample includes non-Hispanic white population aged 70+ only. The sample size is 3,147 participants in England and 5,330 participants in the United States. All estimates are age-gender standardized to the overall 2016 aged 70+ white population in England. 'Past Health Conditions' controls for whether an individual had any cardiometabolic diseases (diabetes, heart disease, and stroke) and/or psychiatric conditions 4 years prior. 'Ever Smoked' controls for whether an individual has ever smoked. BMI stands for Body Mass Index. 'Past BMI' includes dummy variables to control for whether an individual is classed as underweight, normal weight, *overweight* or *obese*. BMI measurements are based on when an individual first entered the survey, which is at least 10 years prior. We assume the effect of each control is constant across countries and age/gender groups. The reference groups are: No past health conditions; never smoked; and normal weight.

For peer review only

For peer review only - http://bmjopen.bmj.com/site/about/guidelines.xhtml

Figure A1: SES Gradient of Dementia, US vs England, Controlling for Past Health

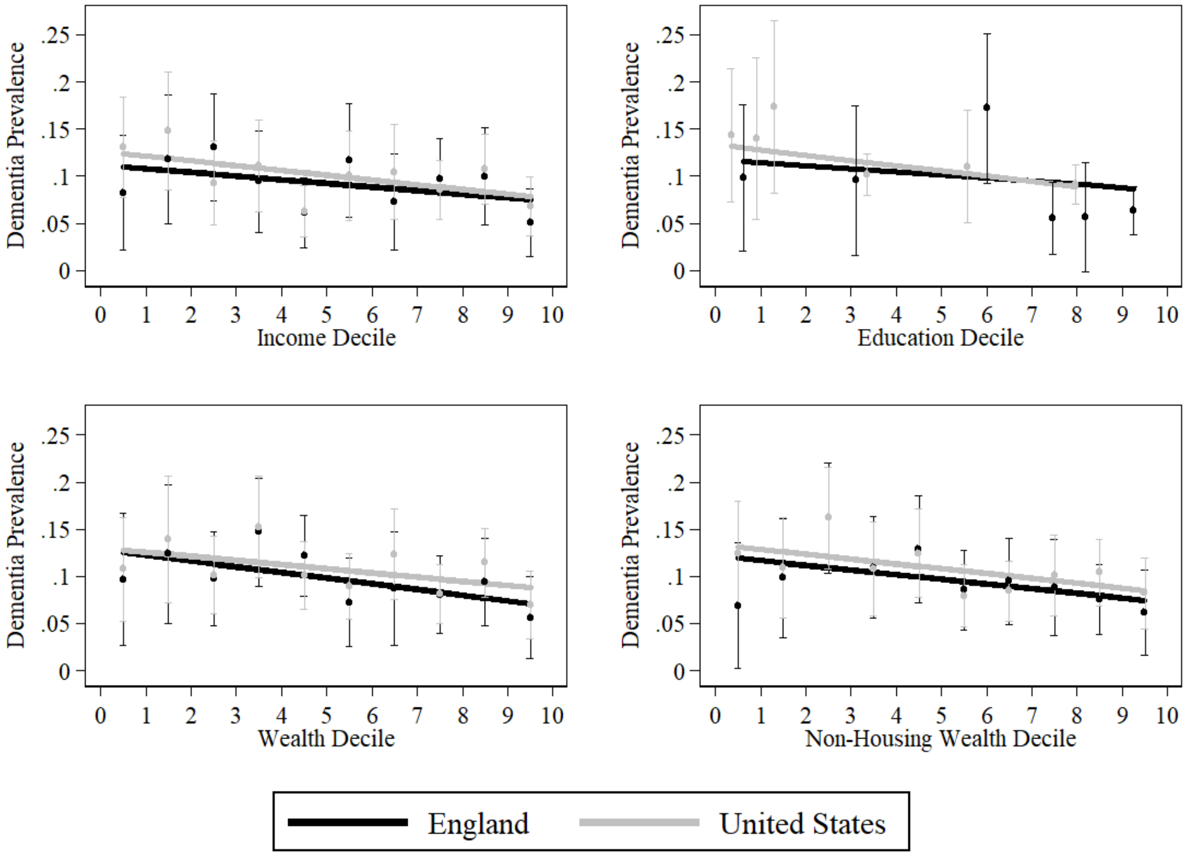

Notes: The points in this figure represent the mean age-gender standardized dementia prevalence for each country by socioeconomic status (SES) after controlling for past health and behaviour, along with 95% confidence interval for these predictions. The solid lines represent the fitted Socioeconomic Index of Inequality (SII) for each country. The values of the SII and the corresponding Relative Index of Inequality (RII) are listed in Table A4.

For peer review only - http://bmjopen.bmj.com/site/about/guidelines.xhtml

Table A9: Prevalence of Dementia, United States vs. England, Full-Sample

	England		United States			
	Age-Gender Standardized Prevalence [95% CI]		Age-Gender Standardized Prevalence [95% CI]		Difference	p-value
All	0.099	[0.091,0.108]	0.124	[0.118,0.129]	0.024	<0.0001
Household Income Decile						
1 (Lowest)	0.116	[0.091,0.140]	0.211	[0.192,0.230]	0.095	<0.0001
2	0.126	[0.101,0.152]	0.161	[0.144,0.178]	0.035	0.025
3	0.124	[0.098,0.150]	0.141	[0.120,0.162]	0.017	0.32
4	0.100	[0.072,0.127]	0.143	[0.124,0.162]	0.044	0.0094
5	0.101	[0.078,0.124]	0.097	[0.082,0.111]	-0.004	0.76
6	0.102	[0.072,0.131]	0.104	[0.086,0.121]	0.002	0.91
7	0.061	[0.043,0.079]	0.108	[0.088,0.127]	0.047	0.0006
8	0.088	[0.057,0.118]	0.085	[0.069,0.102]	-0.002	0.91
9	0.081	[0.041,0.122]	0.089	[0.070,0.108]	0.008	0.74
10 (Highest)	0.057	[0.034,0.080]	0.076	[0.055,0.098]	0.020	0.22
Years of Schooling						
9 or less	0.128	[0.103,0.153]	0.212	[0.195,0.230]	0.084	<0.0001
10	0.099	[0.078,0.119]	0.143	[0.118,0.167]	0.044	0.0072
11	0.097	[0.079,0.116]	0.119	[0.093,0.144]	0.021	0.19
12	0.069	[0.041,0.097]	0.134	[0.124,0.143]	0.064	<0.0001
13	0.063	[0.040,0.085]	0.118	[0.095,0.141]	0.055	0.00071
14 or more	0.055	[0.039,0.071]	0.086	[0.078,0.094]	0.031	0.00071
Household Wealth Decile						
1 (Lowest)	0.172	[0.136,0.207]	0.219	[0.198,0.241]	0.047	0.024
2	0.118	[0.093,0.144]	0.169	[0.151,0.188]	0.051	0.0014
3	0.105	[0.078,0.132]	0.138	[0.120,0.155]	0.032	0.049
4	0.090	[0.068,0.111]	0.120	[0.105,0.135]	0.030	0.023
5	0.118	[0.093,0.143]	0.116	[0.100,0.132]	-0.002	0.89
6	0.077	[0.056,0.099]	0.097	[0.084,0.110]	0.020	0.13
7	0.073	[0.047,0.098]	0.092	[0.076,0.107]	0.019	0.21
8	0.093	[0.067,0.118]	0.103	[0.086,0.120]	0.010	0.52
9	0.080	[0.047,0.113]	0.101	[0.084,0.118]	0.021	0.27
10 (Highest)	0.060	[0.040,0.081]	0.074	[0.058,0.089]	0.013	0.30
Household Non-Housing Wealth Decile						
1 (Lowest)	0.147	[0.113,0.180]	0.215	[0.195,0.235]	0.068	0.00055
2	0.114	[0.089,0.140]	0.184	[0.165,0.203]	0.070	<0.0001
3	0.121	[0.095,0.148]	0.148	[0.129,0.168]	0.027	0.11
4	0.100	[0.076,0.125]	0.129	[0.112,0.147]	0.029	0.054
5	0.110	[0.084,0.135]	0.104	[0.088,0.119]	-0.006	0.70
6	0.093	[0.067,0.118]	0.099	[0.085,0.113]	0.006	0.66
7	0.098	[0.072,0.124]	0.085	[0.071,0.098]	-0.013	0.37
8	0.079	[0.052,0.106]	0.090	[0.075,0.105]	0.011	0.48
9	0.057	[0.035,0.078]	0.094	[0.077,0.110]	0.037	0.0076
10 (Highest)	0.065	[0.045,0.085]	0.083	[0.067,0.099]	0.018	0.15

Notes: This table presents the estimates as Table 1 for the full sample aged 70+, including non-whites. The US sample includes 5,330 non-Hispanic white participants and 1,835 non-white participants. The England sample includes 3,147 non-Hispanic white participants and 77 non-white participants. All estimates are age-gender standardized to the overall 2016 aged 70+ white population in England. The difference is calculated as the prevalence in the US minus prevalence in England.

For peer review only - http://bmjopen.bmj.com/site/about/guidelines.xhtml

For peer review only

Table A10: Difference in Prevalence of Dementia with Controls, Full Sample

Whole Sample:					
England Prevalence	0.099	0.088	0.088	0.084	0.085
	[0.091 to 0.108]	[0.079 to 0.098]	[0.076 to 0.1]	[0.068 to 0.1]	[0.068 to 0.103]
United States	0.124	0.107	0.107	0.105	0.123
	[0.118 to 0.129]	[0.1 to 0.114]	[0.097 to 0.117]	[0.093 to 0.118]	[0.109 to 0.137]
Percentage Point Difference	2.44	1.87	1.90	2.14	3.80
p-value	<0.0001	<0.0001	0.00015	0.00011	<0.0001
Excluding Lowest Income Decile:					
England Prevalence	0.096	0.086	0.086	0.087	0.090
	[0.087 to 0.105]	[0.076 to 0.096]	[0.073 to 0.099]	[0.071 to 0.103]	[0.072 to 0.108]
United States	0.113	0.098	0.099	0.100	0.117
	[0.107 to 0.118]	[0.091 to 0.106]	[0.089 to 0.108]	[0.086 to 0.113]	[0.103 to 0.132]
Percentage Point Difference	1.64	1.25	1.27	1.24	2.70
p-value	0.0024	0.020	0.019	0.030	<0.0001
Lowest Income Decile:					
England Prevalence	0.116	0.107	0.106	0.078	0.099
	[0.091 to 0.140]	[0.077 to 0.138]	[0.062 to 0.15]	[0.026 to 0.13]	[0.036 to 0.162]
United States	0.211	0.187	0.186	0.161	0.195
	[0.192 to 0.230]	[0.16 to 0.214]	[0.149 to 0.223]	[0.117 to 0.206]	[0.138 to 0.252]
Percentage Point Difference	9.54	7.93	7.96	8.37	9.60
p-value	<0.0001	<0.0001	<0.0001	<0.0001	<0.0001
Control for:					
Past Health Conditions		✓	✓	✓	✓
Ever Smoked			✓	✓	✓
Past BMI				✓	✓
Education					✓

Notes: Full sample, population aged 70+, including minorities. The sample sizes are 3,224 participants in England, and 7,165 participants in the United States. All estimates are age-gender standardized to the overall 2016 aged 70+ white population in England. The difference is calculated as the prevalence in the US minus prevalence in England. Differences are displayed as percentage points. 'Past Health Conditions' controls for whether an individual had any cardiometabolic diseases (diabetes, heart disease, and stroke) and/or psychiatric conditions 4 years prior. 'Ever Smoked' controls for whether an individual has ever smoked. BMI stands for Body Mass Index. 'Past BMI' includes dummy variables to control for whether an individual is classed as underweight, normal weight, *overweight* or *obese*. BMI measurements are based on when an individual first entered the survey, which is at least 10 years prior. We assume the effect of each control is constant across countries and age/gender groups. The reference groups are: No past health conditions; never smoked; normal weight; and high-school education.

For peer review only

Table A11: Prevalence of Dementia in United States, Whites versus Non-whites

	US Non-White and Hispanic		US Non-Hispanic White			
	Age-Gender Standardized Prevalence [95% CI]		Age-Gender Standardized Prevalence [95% CI]		Difference	p-value
All	0.182	[0.169 to 0.195]	0.112	[0.106 to 0.118]	-0.070	<0.0001
Household Income Decile						
1 (Lowest)	0.224	[0.199 to 0.249]	0.204	[0.178 to 0.231]	-0.020	0.28
2	0.211	[0.180 to 0.243]	0.143	[0.123 to 0.163]	-0.068	0.00034
3	0.220	[0.158 to 0.282]	0.121	[0.102 to 0.140]	-0.099	0.0023
4	0.202	[0.165 to 0.240]	0.133	[0.111 to 0.155]	-0.069	0.0018
5	0.116	[0.076 to 0.156]	0.094	[0.079 to 0.109]	-0.022	0.31
6	0.164	[0.097 to 0.230]	0.098	[0.081 to 0.116]	-0.065	0.065
7	0.129	[0.080 to 0.178]	0.107	[0.086 to 0.128]	-0.022	0.42
8	0.107	[0.047 to 0.166]	0.084	[0.067 to 0.101]	-0.023	0.47
9	0.072	[0.026 to 0.118]	0.090	[0.069 to 0.110]	0.017	0.50
10 (Highest)	0.084	[0.038 to 0.129]	0.077	[0.055 to 0.099]	-0.006	0.81
Years of Schooling						
9 or less	0.235	[0.212 to 0.258]	0.190	[0.162 to 0.218]	-0.045	0.015
10	0.172	[0.127 to 0.218]	0.137	[0.109 to 0.165]	-0.035	0.19
11	0.144	[0.109 to 0.179]	0.109	[0.080 to 0.139]	-0.035	0.14
12	0.198	[0.170 to 0.227]	0.124	[0.114 to 0.133]	-0.074	<0.0001
13	0.159	[0.101 to 0.218]	0.116	[0.090 to 0.141]	-0.043	0.18
14 or more	0.099	[0.079 to 0.119]	0.085	[0.076 to 0.093]	-0.015	0.20
Household Wealth Decile						
1 (Lowest)	0.228	[0.201 to 0.255]	0.208	[0.176 to 0.240]	-0.020	0.36
2	0.208	[0.179 to 0.237]	0.154	[0.131 to 0.177]	-0.054	0.0043
3	0.184	[0.142 to 0.227]	0.120	[0.102 to 0.138]	-0.064	0.0063
4	0.142	[0.111 to 0.174]	0.115	[0.098 to 0.132]	-0.027	0.13
5	0.159	[0.117 to 0.202]	0.108	[0.092 to 0.124]	-0.051	0.027
6	0.120	[0.084 to 0.155]	0.093	[0.080 to 0.107]	-0.026	0.18
7	0.156	[0.086 to 0.226]	0.088	[0.072 to 0.103]	-0.068	0.063
8	0.197	[0.134 to 0.259]	0.099	[0.081 to 0.116]	-0.098	0.0031
9	0.122	[0.065 to 0.178]	0.099	[0.082 to 0.117]	-0.022	0.46
10 (Highest)	0.045	[0.018 to 0.073]	0.075	[0.059 to 0.090]	0.029	0.071
Household Non-Housing Wealth Decile						
1 (Lowest)	0.231	[0.207 to 0.256]	0.197	[0.167 to 0.227]	-0.034	0.085
2	0.204	[0.177 to 0.231]	0.177	[0.151 to 0.203]	-0.027	0.16
3	0.187	[0.142 to 0.232]	0.135	[0.114 to 0.155]	-0.052	0.036
4	0.158	[0.128 to 0.188]	0.123	[0.104 to 0.143]	-0.034	0.059
5	0.106	[0.069 to 0.143]	0.102	[0.086 to 0.118]	-0.004	0.85
6	0.099	[0.063 to 0.134]	0.098	[0.084 to 0.113]	0.000	0.99
7	0.124	[0.066 to 0.182]	0.083	[0.069 to 0.097]	-0.040	0.18
8	0.140	[0.069 to 0.210]	0.088	[0.073 to 0.103]	-0.051	0.16
9	0.127	[0.054 to 0.201]	0.092	[0.075 to 0.108]	-0.035	0.36
10 (Highest)	0.069	[0.050 to 0.088]	0.084	[0.068 to 0.101]	0.016	0.23

Notes: The sample includes 5,330 non-Hispanic white participants and 1,835 non-white and Hispanic participants. All estimates are age-gender standardized to the overall 2016 aged 70+ white population in England. The difference is calculated as the non-white and Hispanic prevalence in the US minus the non-Hispanic white prevalence in the US.

For peer review only

Appendix References

1 Langa KM, Larson EB, Crimmins EM, *et al.* A comparison of the prevalence of dementia in the United States in 2000 and 2012. *JAMA Intern Med* 2017;**177**:51–8.

2 Hudomiet P, Hurd MD, Rohwedder S. The relationship between lifetime out-of-pocket medical expenditures, dementia, and socioeconomic status in the US. *J Econ Ageing* 2019;**14**:100181.

3 Rusmaully J, Dugravot A, Moatti J-P, *et al.* Contribution of cognitive performance and cognitive decline to associations between socioeconomic factors and dementia: A cohort study. *PLoS Med* 2017;**14**:e1002334.

4 Cadar D, Lassale C, Davies H, *et al.* Individual and area-based socioeconomic factors associated with dementia incidence in England: evidence from a 12-year follow-up in the English longitudinal study of ageing. *JAMA Psychiatry* 2018;**75**:723–32.

5 Rocca WA, Petersen RC, Knopman DS, *et al.* Trends in the incidence and prevalence of Alzheimer's disease, dementia, and cognitive impairment in the United States. *Alzheimers Dement* 2011;**7**:80–93.

6 Basu R. Education and dementia risk: results from the Aging Demographics and Memory Study. *Res Aging* 2013;**35**:7–31.

7 Nguyen TT, Tchetgen EJT, Kawachi I, *et al.* Instrumental variable approaches to identifying the causal effect of educational attainment on dementia risk. *Ann Epidemiol* 2016;**26**:71–6.

8 Crimmins EM, Kim JK, Langa KM, *et al.* Assessment of cognition using surveys and neuropsychological assessment: the Health and Retirement Study and the Aging, Demographics, and Memory Study. *J Gerontol B Psychol Sci Soc Sci* 2011;**66**:i162–71.

9 Garcia MA, Saenz J, Downer B, *et al.* The role of education in the association between race/ethnicity/nativity, cognitive impairment, and dementia among older adults in the United States. *Demogr Res* 2018;**38**:155.

10 Weden MM, Shih RA, Kabeto MU, *et al.* Secular trends in dementia and cognitive impairment of US rural and urban older adults. *Am J Prev Med* 2018;**54**:164–72.

11 Sonnega A, Faul JD, Ofstedal MB, *et al.* Cohort profile: the health and retirement study (HRS). *Int J Epidemiol* 2014;**43**:576–85.

12 Steptoe A, Breeze E, Banks J, *et al.* Cohort profile: the English longitudinal study of ageing. *Int J Epidemiol* 2013;**42**:1640–8.

13 Hurd MD, Martorell P, Delavande A, *et al.* Monetary costs of dementia in the United States. *N Engl J Med* 2013;**368**:1326–34.

14 Langa KM, Plassman BL, Wallace RB, *et al.* The Aging, Demographics, and Memory Study: study design and methods. *Neuroepidemiology* 2005;**25**:181–91.

15 Gianattasio KZ, Wu Q, Glymour MM, *et al.* Comparison of methods for algorithmic classification of dementia status in the health and retirement study. *Epidemiol Camb Mass* 2019;**30**:291.

16 Staff H. Sample sizes and response rates. *Ann Arbor MI Surv Res Cent Inst Soc Res Univ Mich Online Verfügbar Unter Httpshrs Isr Umich Edusitesdefaultf IlesbiblioResponseRates2017 Pdf Zugriff Am* 2017;**28**.

17 Banks J, Nazroo J, Steptoe A. Wave 6. *Inst Fisc Stud* 2014.

For peer review only - http://bmjopen.bmj.com/site/about/guidelines.xhtml

STROBE (Strengthening The Reporting of OBservational Studies in Epidemiology) Checklist

A checklist of items that should be included in reports of observational studies. You must report the page number in your manuscript where you consider each of the items listed in this checklist. If you have not included this information, either revise your manuscript accordingly before submitting or note N/A.

Note: An Explanation and Elaboration article discusses each checklist item and gives methodological background and published examples of transparent reporting. The STROBE checklist is best used in conjunction with this article (freely available on the Web sites of PLoS Medicine at http://www.plosmedicine.org/, Annals of Internal Medicine at http://www.annals.org/, and Epidemiology at http://www.epidem.com/). Information on the STROBE Initiative is available at www.strobe-statement.org.

Section and Item	Item No.	Recommendation	Reported on Page No.
**Title and Abstract**	1	(*a*) Indicate the study's design with a commonly used term in the title or the abstract	1
		(*b*) Provide in the abstract an informative and balanced summary of what was done and what was found	1
**Introduction**			
Background/Rationale	2	Explain the scientific background and rationale for the investigation being reported	3
Objectives	3	State specific objectives, including any prespecified hypotheses	3
**Methods**			
Study Design	4	Present key elements of study design early in the paper	3-4
Setting	5	Describe the setting, locations, and relevant dates, including periods of recruitment, exposure, follow-up, and data collection	3-4
Participants	6	(*a*) *Cohort study*—Give the eligibility criteria, and the sources and methods of selection of participants. Describe methods of follow-up	3
		Case-control study—Give the eligibility criteria, and the sources and methods of case ascertainment and control selection. Give the rationale for the choice of cases and controls	
		Cross-sectional study—Give the eligibility criteria, and the sources and methods of selection of participants	
		(*b*) *Cohort study*—For matched studies, give matching criteria and number of exposed and unexposed	
		Case-control study—For matched studies, give matching criteria and the number of controls per case	
Variables	7	Clearly define all outcomes, exposures, predictors, potential confounders, and effect modifiers. Give diagnostic criteria, if applicable	4-5

For peer review only

Section and Item	Item No.	Recommendation	Reported on Page No.
Data Sources/ Measurement	8*	For each variable of interest, give sources of data and details of methods of assessment (measurement). Describe comparability of assessment methods if there is more than one group	4
Bias	9	Describe any efforts to address potential sources of bias	4-5
Study Size	10	Explain how the study size was arrived at	3
Quantitative Variables	11	Explain how quantitative variables were handled in the analyses. If applicable, describe which groupings were chosen and why	4-5
Statistical Methods	12	(*a*) Describe all statistical methods, including those used to control for confounding	4-5
		(*b*) Describe any methods used to examine subgroups and interactions	4-5
		(*c*) Explain how missing data were addressed	4-5
		(*d*) *Cohort study*—If applicable, explain how loss to follow-up was addressed	4-5
		Case-control study—If applicable, explain how matching of cases and controls was addressed	
		Cross-sectional study—If applicable, describe analytical methods taking account of sampling strategy	
		(*e*) Describe any sensitivity analyses	4-5
**Results**			
Participants	13*	(a) Report numbers of individuals at each stage of study—eg numbers potentially eligible, examined for eligibility, confirmed eligible, included in the study, completing follow-up, and analysed	9
		(b) Give reasons for non-participation at each stage	
		(c) Consider use of a flow diagram	
Descriptive Data	14*	(a) Give characteristics of study participants (eg demographic, clinical, social) and information on exposures and potential confounders	6,11
		(b) Indicate number of participants with missing data for each variable of interest	
		(c) *Cohort study*—Summarise follow-up time (eg, average and total amount)	
Outcome Data	15*	*Cohort study*—Report numbers of outcome events or summary measures over time	5-6
		Case-control study—Report numbers in each exposure category, or summary measures of exposure	
		Cross-sectional study—Report numbers of outcome events or summary measures	

For peer review only

Section and Item	Item No.	Recommendation	Reported on Page No.
Main Results	16	(*a*) Give unadjusted estimates and, if applicable, confounder-adjusted estimates and their precision (eg, 95% confidence interval). Make clear which confounders were adjusted for and why they were included	5-6
		(*b*) Report category boundaries when continuous variables were categorized	
		(*c*) If relevant, consider translating estimates of relative risk into absolute risk for a meaningful time period	10
Other Analyses	17	Report other analyses done—eg analyses of subgroups and interactions, and sensitivity analyses	5-6,9-11
**Discussion**			
Key Results	18	Summarise key results with reference to study objectives	6-7
Limitations	19	Discuss limitations of the study, taking into account sources of potential bias or imprecision. Discuss both direction and magnitude of any potential bias	8
Interpretation	20	Give a cautious overall interpretation of results considering objectives, limitations, multiplicity of analyses, results from similar studies, and other relevant evidence	7
Generalisability	21	Discuss the generalisability (external validity) of the study results	7
**Other Information**			
Funding	22	Give the source of funding and the role of the funders for the present study and, if applicable, for the original study on which the present article is based	12

*Give information separately for cases and controls in case-control studies and, if applicable, for exposed and unexposed groups in cohort and cross-sectional studies.

Once you have completed this checklist, please save a copy and upload it as part of your submission. DO NOT include this checklist as part of the main manuscript document. It must be uploaded as a separate file.

For peer review only

BMJ Open

Dementia and Disadvantage in the United States and England: Population-based comparative study

Journal:	*BMJ Open*
Manuscript ID	bmjopen-2020-045186.R2
Article Type:	Original research
Date Submitted by the Author:	13-Jul-2021
Complete List of Authors:	Arapakis, Karolos; UCL, Department of Economics<br>Brunner, Eric; University College London, Epidemiology and Public Health<br>French, Eric; Cambridge University, Department of Economics; Institute for Fiscal Studies<br>McCauley, Jeremy; University of Bristol, School of Economics
**Primary Subject Heading**:	Public health
Secondary Subject Heading:	Health economics, Geriatric medicine, Neurology
Keywords:	Dementia < NEUROLOGY, PUBLIC HEALTH, HEALTH ECONOMICS

SCHOLARONE™ Manuscripts

I, the Submitting Author has the right to grant and does grant on behalf of all authors of the Work (as defined in the below author licence), an exclusive licence and/or a non-exclusive licence for contributions from authors who are: i) UK Crown employees; ii) where BMJ has agreed a CC-BY licence shall apply, and/or iii) in accordance with the terms applicable for US Federal Government officers or employees acting as part of their official duties; on a worldwide, perpetual, irrevocable, royalty-free basis to BMJ Publishing Group Ltd ("BMJ") its licensees and where the relevant Journal is co-owned by BMJ to the co-owners of the Journal, to publish the Work in this journal and any other BMJ products and to exploit all rights, as set out in our licence.

The Submitting Author accepts and understands that any supply made under these terms is made by BMJ to the Submitting Author unless you are acting as an employee on behalf of your employer or a postgraduate student of an affiliated institution which is paying any applicable article publishing charge ("APC") for Open Access articles. Where the Submitting Author wishes to make the Work available on an Open Access basis (and intends to pay the relevant APC), the terms of reuse of such Open Access shall be governed by a Creative Commons licence – details of these licences and which Creative Commons licence will apply to this Work are set out in our licence referred to above.

Other than as permitted in any relevant BMJ Author's Self Archiving Policies, I confirm this Work has not been accepted for publication elsewhere, is not being considered for publication elsewhere and does not duplicate material already published. I confirm all authors consent to publication of this Work and authorise the granting of this licence.

For peer review only

Dementia and Disadvantage in the United States and England:
Population-based comparative study

By Karolos Arapakis, Eric J Brunner, Eric French, Jeremy McCauley*

Objectives – To compare dementia prevalence and how it varies by socioeconomic status (SES) across the United States and England.

Design – Population-based comparative study.

Setting - Non-Hispanic whites aged over 70 population in the United States and England.

Participants – Data from the Health and Retirement Study (HRS) and the English Longitudinal Study of Ageing (ELSA), which are harmonised, nationally representative panel studies. The sample includes 5,330 and 3,147 individuals in the United States and England, respectively.

Main outcome measures – Between country differences in age-gender standardized dementia prevalence, across the SES gradient. Dementia prevalence was estimated in each country using an algorithm based on an identical battery of demographic, cognitive, and functional measures.

Results – Dementia prevalence is higher among the disadvantaged in both countries, with the United States being more unequal according to four measures of SES. Overall prevalence was lower in England at 9.7% (95% CI, 8.9% to 10.6%) than the US at 11.2% (95% CI, 10.6% to 11.8%), a difference of 1.4 percentage points (pp) (p = 0.0055). Most of the between country difference is driven by the bottom of the SES distribution. In the lowest income decile individuals in the US had 7.3 pp (p <0.0001) higher prevalence than in England. Once past health factors and education were controlled for, most of the within country inequalities disappeared; however, the cross-country difference in prevalence for those in lowest income decile remained disproportionately high.

Conclusions – There is inequality in dementia prevalence according to income, wealth, and education in both the US and England. England has lower dementia prevalence and a less steep SES gradient. Most of the cross-country difference is concentrated in the lowest SES group, which provides evidence that disadvantage in the United States is a disproportionately high risk factor for dementia.

* Department of Economics, University College London, London, UK (K Arapakis MSc, Prof E French PhD)
Department of Epidemiology and Public Health, University College London, London, UK (Prof E J Brunner PhD)
Department of Economics, University of Bristol, Bristol, UK (J McCauley PhD)
Institute for Fiscal Studies, London, UK (E French)
Correspondence to: Jeremy McCauley, School of Economics, University of Bristol, Bristol BS8 1TU, UK **jeremy.mccauley@bristol.ac.uk**

For peer review only - http://bmjopen.bmj.com/site/about/guidelines.xhtml

Strengths and limitations of this study

- This is the first study to compare dementia prevalence across countries using the same survey methodology and the exact same measure of dementia. The surveys have similar sample selection and questionnaire design. We standardise our estimates by age and gender to the English population aged over 70 in 2016. Any differences in overall prevalence across the two countries should represent true differences.

- We measure the SES gradient of dementia across four different measures of SES: income; education; wealth; and non-housing wealth.

- Dementia disproportionately affects the most disadvantaged in in both countries, although the gradient is steeper in the US according to all four measures of SES.

- We do not ascertain dementia directly, but predict cases using a common battery of measures in ELSA and HRS. One of the SES measures, education, is also used as a predictor of dementia.

For peer review only

For peer review only - http://bmjopen.bmj.com/site/about/guidelines.xhtml

Introduction

Dementia, a severe and irreversible decline in memory and other cognitive functions, is a major and increasing global health challenge. It is the fifth leading cause of death globally and is one of the most common comorbidities for Covid-19 morbidity.[1,2] It results in large social and economic costs.[3,4]

Americans are more likely to be in poor health than their English counterparts in multiple dimensions, including heart disease and diabetes.[5] These differences are large along all points of the socioeconomic status (SES) gradient, although the gradient is generally steeper in the US. While the SES gradient for many diseases has been well established,[6–8] only a few studies have focused specifically on dementia.[9,10] The available evidence is summarized in Table A1 in the online appendix. The evidence of the SES gradient for dementia is also less clear, as in England while a strong association has been established between wealth and dementia incidence, the same was not observed for education level.[11]

The Global Burden of Disease Study (GBD) reported that in 2017 among those aged over 70, the US had a lower overall prevalence of dementia at 7.89% compared with the UK at 8.91%.[12] However, the GBD has identified substantial heterogeneity in case-ascertainment methods throughout the dementia literature, resulting in location-specific inconsistencies and potentially biased cross-country comparisons. This has led to calls for analyses with more consistent and comparable measures of dementia to inform policy makers, researchers, and clinicians about global differences in dementia.[13]

In this study, we compared dementia prevalence in England and the US among non-Hispanic whites aged 70+, and how it varied across the SES gradient of each country. Location-specific inconsistencies caused by differences in diagnostic practices were not an issue in our study because we used an identical case definition for dementia, and the surveys in the analysis shared the same design and sampling techniques. More specifically, we used two large surveys, the US Health and Retirement Study (HRS) and the English Longitudinal Study of Ageing (ELSA), that contain a battery of the same demographic, cognitive, and functional measures, and we applied the same prediction algorithm in both countries to detect undiagnosed as well as diagnosed cases. We compared dementia prevalence within and across England and the US using important indicators of SES, specifically: income; education; wealth; and non-housing wealth.

Methods

Description of Surveys

Data were extracted from the 2016 and earlier waves of the HRS and ELSA, which are nationally representative biennial surveys of the US and English populations, respectively.[14,15] Both the HRS and ELSA follow respondents longitudinally until death, with new cohorts entering to maintain population representativeness as the study sample gets older. The design of ELSA was based on the HRS, making the two surveys analogous, with both collecting data on health, ability, demographics, employment, and wealth. In addition to measuring health conditions and difficulties respondents have with Activities of Daily Living (ADLs) and Instrumental Activities of Daily Living (IADLs), sample members also have their cognitive function assessed. A range of tests adapted from the Telephone Interview for Cognitive Status (TICS) have been carried out in HRS since 1996 and ELSA since 2014. If a sample member was unable to respond in person, a proxy respondent was asked questions about the respondent's change in memory. Both surveys

For peer review only - http://bmjopen.bmj.com/site/about/guidelines.xhtml

have a high response rate, which is displayed in the online appendix Table A2. We describe these surveys in more detail in the online appendix.

Cohort Description

Our samples are restricted to non-Hispanic whites over the age of 70 years old that live in the community or in nursing homes in 2016. This provides a study sample of 5,330 participants in the HRS and 3,147 participants in ELSA. We restrict our sample to non-Hispanic whites to ensure estimates are comparable across countries. Summary statistics of both the raw and selected samples are displayed in online appendix Tables A3 and A4, and specifications that include ethnic minorities are also displayed.

Patient and Public Involvement

No study participants were involved in setting the research question or outcome measures, nor were they involved in any other area of the design, implementation, and analysis of the study. There are no direct plans to disseminate the results of the research to study participants.

Dementia Case Definition

The HRS included a detailed clinical substudy (ADAMS: Aging, Demographics and Memory Study) of 856 sample members aged 70+ who completed an in-depth in-home assessment of cognitive status conducted by experienced teams at the Duke University Dementia Epidemiology Research Center who diagnosed each participant as normal, cognitively impaired but not demented (CIND), or demented.[3] Data from ADAMS is regarded as the gold-standard dementia diagnoses against which to train algorithms to predict dementia.[16] Hurd et al. estimated separate ordered probit models in the ADAMS subsample for self- and proxy-respondents to generate a predictive algorithm for cognitive status, based on the ADAMS diagnoses, for the whole HRS sample. The algorithm uses a range of variables including demographic information, Activities of Daily Living (ADLs), Instrumental Activities of Daily Living (IADLs), TICS questionnaire, as well as the change in these variables across waves.[3] Proxy respondents had a separate predictive algorithm as they were asked a different set of questions from self-respondents, which included the short form of the Informant Questionnaire on Cognitive Decline in the Elderly (IQCODE). The use of a proxy to assess cognitive decline and dementia in elderly people is a recognised accepted standard method for identifying severe cognitive impairment and has been validated many times.[17] Importantly, the same set of questions used in the Hurd et al. algorithm is asked of self- and proxy-respondents in both HRS and ELSA. Summary statistics for a variety of predictors are displayed in online appendix Table A5.

We applied Hurd et al.'s predictive algorithm to estimate the probably of dementia for those in the HRS sample in 2016 and extended the prediction to the ELSA sample. The algorithm predicts the probability of dementia in the following year; therefore, we predicted dementia prevalence in 2017. Hurd et al's predictive algorithm has been shown to have an accuracy (percentage correctly classified as demented or non-demented) of 94%, sensitivity of 65%, and a specificity of 98% in the estimation sample.[16] An in-depth discussion of the predictive algorithm procedure can be found in the online appendix.

For peer review only - http://bmjopen.bmj.com/site/about/guidelines.xhtml

Non-response for people unable or unwilling to participate in the survey is important when attempting to estimate dementia prevalence across the population. While attrition exists in both surveys, it is unlikely to significantly affect our estimates since among older ELSA and HRS respondents there is no statistically significant correlation between attrition and prior health or the SES indicators of education, income and wealth.[18]

Measures of Socioeconomic Status

We considered four measures of SES: income; education; wealth; and non-housing wealth. Income is measured as current household income from all sources. For education we used total years of schooling. “Wealth is measured as the sum of all household reported savings, stocks, bonds, business wealth, other assets, and the value of housing assets (e.g., properties) after financial debt and mortgage debt has been subtracted. Non-housing wealth is the same measure as wealth but excludes housing assets and mortgage debt, and therefore measure wealth that can be more easily converted to cash. Wealth and non-housing wealth are both measured from 4 years prior to minimize reverse causality, as medical expenses associated with dementia are high and may run down wealth.[4] For each measure, we created a SES gradient by ranking individuals based on that measure. For income, wealth, and non-housing wealth, we assigned everyone to a decile in their respective country. For education, we ranked individuals according to their number of years of schooling.

Statistical Analysis

We created a pooled dataset of the two surveys. In our statistical analysis we used HRS and ELSA sampling weights to adjust for nonresponse and for the sampling design of the surveys. To make both within country estimates along the SES gradient and cross-country estimates directly comparable, estimates were age-gender standardized to the English population aged over 70 in 2016 using direct standardization, categorising the population into ten groups: five age bands (70-74; 75-79; 80-84; 85-89; and 90+) by gender. We estimated the prevalence of dementia in each country, their difference, and compared the prevalence along the four SES gradients. For each estimate presented we computed the corresponding 95% confidence interval (CI), and for any differences we computed the corresponding p-values. For each SES factor, as well as estimating the age-gender standardized prevalence along the gradient, we calculated the Relative Index of Inequality (RII) and Slope Index of Inequality (SII) using generalized linear models (log binomial regression) with logarithmic and identity link functions, respectively. The RII can be interpreted as the relative likelihood of dementia prevalence of those in the lowest SES group compared to those in the highest, and the SII can be interpreted as the absolute effect on dementia probability of moving from the lowest SES group to the highest.[19] To assess whether any observed differences could be explained by disparities in past health risk factors across countries we conditioned on a variety of risk factors and assessed how our estimates changed. Where possible, when conditioning on these factors we used past health instead of current health to address the problem of reverse causality: i.e., the problem that dementia may cause health problems such as low weight. Statistical analyses were performed using STATA software.

For peer review only - http://bmjopen.bmj.com/site/about/guidelines.xhtml

Results

Table 1 shows the age-gender standardized prevalence of dementia for the aged over 70 white non-Hispanic population in both England and the US. Dementia prevalence is lower in England at 9.7% (95% CI, 8.9% to 10.6%) than the US at 11.2% (95% CI, 10.6% to 11.8), a difference of 1.4 percentage points (pp) that is highly statistically significant (*P* = 0.0055).

Table 1 also shows dementia prevalence for different SES groups, in terms of income, education, wealth, and non-housing wealth. Regardless of the measure of the SES, there is a clear gradient in dementia prevalence, with the most disadvantaged groups in both England and the US having higher dementia prevalence. The gradient is steeper in the US and is driven by significantly higher dementia prevalence for those at the very bottom of the distribution. In the US, those in the lowest income decile have a dementia prevalence of 18.7% (95% CI, 16.6% to 20.8%), which is considerably higher than in England, with a prevalence among those in the lowest decile of 11.4% (95% CI, 8.9% to 13.9%). The difference is highly statistically significant (*P < 0.0001)*. For income deciles above the lowest, the difference across the two countries is much smaller and not statistically significant. This same general pattern is evident across the other measures of SES that we consider, although when using wealth, the difference between those in the bottom decile is not statistically significant.

Figure 1 presents the same dementia prevalence information shown in Table 1, but in graphical format. It also reports the Slope Index of Inequality (SII) for the four different measures of the SES for both countries. In both the US and England, dementia is more prevalent among the more disadvantaged. The gradient tends to be steeper in the US, corresponding to a larger (in absolute value) SII in the US for each SES measure. For income, the SII is -0.062 (95% CI, -0.097 to -0.028) and -0.085 (95% CI, -0.114 to -0.057) for England and the US, respectively. The SIIs are not statistically different. If the lowest income decile is excluded, the SII for England becomes slightly steeper (-0.067 (95% CI, -0.107 to -0.027)) whereas the SII for the US becomes less steep than England (-0.060 (95% CI, -0.093 to -0.027)).

Next, we attempted to understand the potential drivers of these gradients and the differences in the gradients across countries. We extended the analysis to account for cardiometabolic diseases (i.e. diabetes, heart disease and stroke), and behaviours (i.e. smoking and body mass index) as dementia risk factors.[1,20] Previous research showed these factors to be more prevalent in the US than England, especially among the most disadvantaged.[5] Table 2 displays the percentage point difference in dementia prevalence after we controlled for various measures of past health and behaviours. The results are split into three panels: the whole sample; the whole sample excluding those in the lowest income decile; and the lowest income decile. As was shown in Table 1, the difference in the prevalence of dementia between England and the US was 1.43 pp (*P = 0.0055*). Table 2 shows this difference declined to 0.894 pp (*P = 0.11)* when we excluded the lowest income decile. Controlling for past health and behaviours modestly reduced this cross-country difference further: the difference declined by a maximum of 19%. For the lowest income decile, controlling for past health and behaviour reduced the cross-country difference of 7.27 pp (*P < 0.0001*) by a more substantial 33%.

In the online appendix Tables A6-A8 we investigated whether past health and behaviour explained the SES gradient within each country. We found that in England and the US these factors accounted for most of

For peer review only - http://bmjopen.bmj.com/site/about/guidelines.xhtml

the SES gradient, as shown in online appendix Figure A1. However, in the US, prevalence in the lowest income decile remained disproportionately high.

Education has also been shown to be a risk factor for dementia. Table 1 shows that in both the US and England, the less educated have higher dementia prevalence. Controlling for education increased the estimated difference across countries, from 1.43 pp to 2.82 pp, as can be seen in the online appendix Table A7. Education cannot explain these differences since the English have lower educational attainment.

In our main analysis we exclude ethnic minorities, who have higher dementia prevalence and comprise a larger share of the US than the English population. Including minorities increased the estimated difference across countries, from 1.43 pp to 2.42 pp, as can be seen in the online appendix Tables A9 and A10. This is largely caused by the high prevalence of dementia among minorities in the US as displayed in online appendix Table A11.

Discussion

Main findings

Using nationally representative samples of older individuals from England and the US, and applying the same algorithmic procedure to predict dementia in both samples, we showed that in both the US and England dementia is more prevalent among the disadvantaged, and the SES gradient of dementia is steeper in the US. The steeper gradient in the US is largely driven by those in the lowest decile. In both countries, most of the SES gradient disappeared when we controlled for past health related factors, although prevalence for those in lowest income decile in the US remained disproportionately high. If the lowest income decile is excluded from our sample the difference in dementia prevalence across the countries is statistically insignificant, and the remaining SES gradient of dementia is remarkably similar across countries.

While poorer individuals face a higher burden of dementia in both England and the US, the extremely poor in the US face a disproportionately high burden of dementia. Controlling for past health-related factors can explain some, but not all, of the cross-country difference. It can explain up to around one third of the difference for those in the lowest income decile. While past health factors such as adiposity and smoking are correlated with dementia, those in the lowest income decile in the US do not smoke more or have higher BMIs than their English counterparts. Therefore, this cannot explain their disproportionately higher prevalence of dementia. Education also cannot explain the difference, as the US population is more educated at every income decile, and in fact the educational difference masks some of the underlying dementia risk difference between countries.

Adding minorities increased estimated dementia prevalence, especially in the US, because dementia prevalence is higher among minorities, who comprise a higher share of the US than the English population. This fits with prior research which showed dementia prevalence is higher for non-whites and Hispanics.[20,21] We did not observe higher dementia prevalence among Hispanics and non-whites in the US for those in relatively high socioeconomic groups.

For peer review only - http://bmjopen.bmj.com/site/about/guidelines.xhtml

Comparison with previous studies and how findings are an advance on current literature

Previous studies have shown cross-country variation in dementia prevalence. However, substantial heterogeneity in case-ascertainment methods across countries and studies makes interpreting any observed differences difficult. We believe this is the first study to compare dementia prevalence in England and the US using the exact same measure of dementia, thus overcoming previous difficulties in making comparisons across the two countries due differences in diagnostic practices and case definitions. We also compared the SES gradient of dementia in both countries. While some studies have shown in both England and the US those with lower education and less wealth have been found to have higher rates of dementia,[4,9,11,22,23] there are no systematic comparative studies. We compared prevalence along the SES gradient using almost identical measures of income, wealth, and education. Further, we standardized the cross-country comparison for age and gender, using the English over 70 population as the standard population. We found that dementia prevalence is higher and more concentrated among the poorest in the US than England. Detailed disaggregation according to SES measures shows the true extent of the excess burden of dementia in the very poorest group in the US.

We showed that risk factors for dementia such as cardiometabolic diseases, psychiatric conditions, high BMI, smoking have similar affects across countries. Accounting for these risk factors removes most of the SES gradient for both countries, but disproportionately high prevalence remains for the most disadvantaged in the US.

Implications (wider interpretation)

Much research has shown that low income Americans are more likely to be in poor health and die younger than their high-income counterparts.[24] We show that these health differences also extend to dementia prevalence.

While risk factors contribute to higher prevalence among those who are more disadvantaged, those in the US appear to have an undue burden that is caused by risk factors for which we cannot account. One possible explanation is differential access to healthcare. The NHS provides broadly equitable care according to education in the older population after accounting for health status.[25] In the US, the poor often go uninsured, and although virtually every American aged 65 or older is eligible for Medicare, around 20% of Medicare beneficiaries healthcare must be financed out of pocket.[26] The extent to which health care provision below and above aged 65 may account for the relative excess dementia burden in the US is unclear.

The implications of our results are that interventions designed to attempt to prevent dementia should be targeted towards the most disadvantaged. This is especially true in the US. As yet, we are unable to advocate specific measures as we do not yet understand the specific nature of disadvantage in respect to dementia risk.

Strengths and Weaknesses of Analysis

This study has a strong design. Results are directly comparable across England and the US. The same predictive algorithm was applied to both countries, addressing the problem of heterogeneity in case ascertainment which has affected the literature.[1,13] Further, because ELSA and HRS share sample

For peer review only - http://bmjopen.bmj.com/site/about/guidelines.xhtml

selection and questionnaire design, any differences in overall prevalence and SES gradients in prevalence across the two countries should represent true differences. In contrast to the Global Burden Disease study, we find higher dementia prevalence in the US. Furthermore, we measured the SES gradient of dementia across four different measures of SES, with consistent results. Our work also highlights the usefulness of the standardized measure of dementia to allow for meaningful comparisons across countries.

This study had three limitations. First, we do not ascertain dementia directly, but predict cases using a common battery of measures in ELSA and HRS. Importantly, Hurd's prediction algorithm has high accuracy and although our case definition lacks a clinical point of reference in England, it is based on a detailed clinical substudy in the US.[16] Further, cross-cultural subjectivities in reporting of impairment severity are likely to be similar in the US and England (see online appendix Table A5). It would be of great value for future work to use the Harmonized Cognitive Assessment Protocol (HCAP) data to provide a standard clinical point of reference to validate and verify the cross-country dementia prevalence estimates.[27] Second, education is one of the factors in the predictive algorithm for dementia and also one of our measures of SES. The dementia algorithm takes account of the well-documented correlation between education level and cognitive function in adult life. Nevertheless, we found substantial absolute and relative inequalities in dementia prevalence according to education level in the US and UK. Education may be a successful approach for reducing dementia risk.[28] Third, while we show risk factors explain a large proportion of the differences in dementia between the England and the US – although cannot account for the difference in the lowest income decile – there are likely other unmeasured confounding factors that impact dementia prevalence which we do not observe.

Conclusion

Given the large social and economic costs of dementia, there is great value in understanding the scope and burden of dementia in the population along the SES gradient. This study indicates that more disadvantaged individuals face a higher burden of dementia and that the poorest individuals in the US face a disproportionately high burden. The high burden faced by these individuals can be partly but not fully explained by past health factors. We lacked data on other important possible contributing factors such as habitual drug use. Further research is needed to fully understand this issue using data from multiple sources.

For peer review only - http://bmjopen.bmj.com/site/about/guidelines.xhtml

Table 1: Prevalence of Dementia, United States vs. England, 2017. Overall prevalence and prevalence according to four measures of socioeconomic status

	England		United States			
	Age-Gender Standardized Prevalence [95% CI]		Age-Gender Standardized Prevalence [95% CI]		Difference	p-value
All	0.097	[0.089 to 0.106]	0.112	[0.106 to 0.118]	0.014	0.0055
Household Income Decile						
1 (Lowest)	0.114	[0.089 to 0.139]	0.187	[0.166 to 0.208]	0.073	<0.0001
2	0.113	[0.090 to 0.136]	0.141	[0.119 to 0.163]	0.028	0.090
3	0.124	[0.097 to 0.151]	0.111	[0.095 to 0.127]	-0.013	0.42
4	0.099	[0.071 to 0.126]	0.118	[0.099 to 0.137]	0.019	0.26
5	0.094	[0.072 to 0.116]	0.086	[0.069 to 0.102]	-0.008	0.56
6	0.098	[0.070 to 0.127]	0.108	[0.088 to 0.128]	0.010	0.59
7	0.068	[0.042 to 0.093]	0.100	[0.078 to 0.122]	0.032	0.060
8	0.083	[0.053 to 0.114]	0.082	[0.066 to 0.097]	-0.002	0.92
9	0.082	[0.041 to 0.122]	0.093	[0.071 to 0.116]	0.012	0.62
10 (Highest)	0.059	[0.035 to 0.083]	0.077	[0.052 to 0.103]	0.019	0.30
Years of Schooling						
9 or fewer	0.128	[0.101 to 0.154]	0.190	[0.162 to 0.218]	0.062	0.0015
10	0.095	[0.074 to 0.116]	0.137	[0.109 to 0.165]	0.042	0.018
11	0.096	[0.077 to 0.115]	0.109	[0.080 to 0.139]	0.013	0.471
12	0.071	[0.042 to 0.100]	0.124	[0.114 to 0.133]	0.053	0.0006
13	0.061	[0.038 to 0.083]	0.116	[0.090 to 0.141]	0.055	0.0013
14 or more	0.056	[0.039 to 0.073]	0.085	[0.076 to 0.093]	0.029	0.0031
Household Wealth Decile						
1 (Lowest)	0.165	[0.132 to 0.198]	0.187	[0.162 to 0.211]	0.022	0.31
2	0.117	[0.092 to 0.143]	0.149	[0.129 to 0.169]	0.031	0.061
3	0.100	[0.073 to 0.127]	0.107	[0.091 to 0.122]	0.006	0.68
4	0.093	[0.071 to 0.115]	0.115	[0.098 to 0.132]	0.022	0.12
5	0.110	[0.085 to 0.134]	0.091	[0.077 to 0.106]	-0.018	0.21
6	0.080	[0.057 to 0.103]	0.089	[0.074 to 0.103]	0.008	0.55
7	0.070	[0.046 to 0.094]	0.103	[0.084 to 0.123]	0.034	0.034
8	0.092	[0.066 to 0.118]	0.089	[0.072 to 0.106]	-0.003	0.85
9	0.082	[0.048 to 0.116]	0.103	[0.084 to 0.123]	0.021	0.28
10 (Highest)	0.060	[0.038 to 0.081]	0.067	[0.051 to 0.084]	0.008	0.58
Household Non-Housing Wealth Decile						
1 (Lowest)	0.136	[0.103 to 0.168]	0.201	[0.176 to 0.226]	0.065	0.0019
2	0.109	[0.084 to 0.134]	0.137	[0.119 to 0.156]	0.029	0.074
3	0.123	[0.095 to 0.151]	0.131	[0.111 to 0.150]	0.008	0.66
4	0.096	[0.073 to 0.118]	0.101	[0.085 to 0.116]	0.005	0.71
5	0.108	[0.082 to 0.134]	0.107	[0.091 to 0.123]	-0.001	0.95
6	0.093	[0.067 to 0.119]	0.086	[0.070 to 0.101]	-0.007	0.64
7	0.099	[0.072 to 0.125]	0.086	[0.070 to 0.102]	-0.013	0.42
8	0.078	[0.051 to 0.105]	0.090	[0.073 to 0.106]	0.011	0.49
9	0.058	[0.036 to 0.081]	0.092	[0.074 to 0.111]	0.034	0.024
10 (Highest)	0.063	[0.043 to 0.084]	0.079	[0.062 to 0.095]	0.015	0.26

Notes: Sample includes non-Hispanic white population aged 70+ only. The sample size is 3,147 participants in England and 5,330 participants in the United States. All estimates are age-gender standardized to the overall 2016 aged 70+ white population in England. The difference is calculated as the prevalence in the US minus prevalence in England. All deciles are calculated within country.

For peer review only

Figure 1: SES Gradient of Dementia, United States vs England, 2017, according to four measures of socioeconomic status. Absolute and relative inequality shown with 95% confidence intervals.

[Figure 1 Attached separately]

Notes: The points in this figure represent the mean age-gender standardized dementia prevalence for each country by socioeconomic status (SES), along with 95% confidence interval for these predictions. The solid lines represent the fitted Slope Index of Inequality (SII: absolute inequality) for each country. The values of the SII and the corresponding Relative Index of Inequality (RII: relative inequality) are listed in the top right of each figure, with 95% Confidence Intervals in brackets. For education, individuals are ranked based on their years of schooling within each country, and as the US has higher educational attainment, individuals with 14+ years of schooling are the 80th percentile of the US education distribution, but at the 90th percentile of the English educational distribution.

Table 2: Difference in Prevalence of Dementia, United States vs England, 2017. Estimates of the between-country difference taking account of antecedent health status, smoking and degree of adiposity

Whole Sample:					
Percentage Point Difference	1.43	1.34	1.15	1.21	1.18
p-value	0.0055	0.0091	0.025	0.020	0.034
% Difference from Baseline	--	-6%	-19%	-16%	-17%
Excluding Lowest Income Decile:					
Percentage Point Difference	0.89	0.88	0.74	0.75	0.81
p-value	0.11	0.11	0.18	0.18	0.18
% Difference from Baseline	--	-2%	-17%	-16%	-10%
Lowest Income Decile:					
Percentage Point Difference	7.27	6.14	5.45	5.93	4.85
p-value	<0.0001	0.0003	0.002	0.0011	0.012
% Difference from Baseline	--	-15%	-25%	-18%	-33%
Control for:					
Past Cardiometabolic Diseases		✓	✓	✓	✓
Past Psychiatric Conditions			✓	✓	✓
Ever Smoked				✓	✓
Past BMI					✓

Notes: Sample includes non-Hispanic white population aged 70+ only. The sample size is 3,147 participants in England and 5,330 participants in the United States. All estimates are age-gender standardized to the overall 2016 aged 70+ white population in England. The difference is calculated as the prevalence in the US minus prevalence in England. Differences are displayed as percentage points. 'Past Cardiometabolic Diseases' and 'Past Psychiatric Conditions' control for whether an individual says they had the conditions 4 years prior. 'Cardiometabolic Diseases' include diabetes, heart disease, and stroke. 'Smoking' controls for whether an individual has ever smoked. BMI stands for Body Mass Index. 'Past BMI' includes dummy variables to control for whether an individual is classed as underweight, overweight, or obese. BMI values are based on when an individual first entered the survey, which is at least 10 years prior.

For peer review only - http://bmjopen.bmj.com/site/about/guidelines.xhtml

Contributors and Transparency Statement

All authors made a substantial contribution to study conception and design. EF and JM developed the original idea. JM and KA prepared and analysed the data, with inputs from EF and EJB. All authors contributed to interpreting the results and drafting the manuscript. JM and KA serve as guarantors and affirm that the manuscript is an honest, accurate, and transparent account of the study being reported; that no important aspects of the study have been omitted; and that any discrepancies from the study as planned have been explained. All authors had full access to all of the data (including statistical reports and tables) in the study and take responsibility for the integrity of the data and accuracy of the data analysis. The corresponding author (JM) attests that all listed authors meet authorship criteria and that no others meeting the criteria have been omitted. Author names on manuscript are listed in alphabetical order.

Competing interests

All authors have completed the ICMJE uniform disclosure form and declare no support from any organisation for the submitted work; no financial relationships with any organisations that might have an interest in the submitted work in the previous three years; no other relationships or activities that could appear to have influenced the submitted work

Acknowledgments

We thank Paco Martorell and Ken Langa for help with understanding the prediction equations and Liliana French for editorial assistance.

Ethical approval

The HRS has ethical approval from the University of Michigan Institutional Review Board. ELSA has ethical approval from the London Multicentre Research Ethics Committee (MREC/01/2/91). This study did not require additional ethical approval as we conduct secondary analysis of these two publicly available datasets.

Funding

This work was supported by grants from the British Heart Foundation (RG/13/2/30098, RG/16/11/32334), MRRC, ESRC, and Onassis Foundation.

Data sharing

Appendix with details on the formula and calculations is available online. Code and results are available on request from the corresponding author.

For peer review only

References

1 Nichols E, Szoeke CE, Vollset SE, *et al.* Global, regional, and national burden of Alzheimer's disease and other dementias, 1990–2016: a systematic analysis for the Global Burden of Disease Study 2016. *Lancet Neurol* 2019;**18**:88–106.

2 Kuo C-L, Pilling LC, Atkins JL, *et al.* ApoE e4e4 genotype and mortality with COVID-19 in UK Biobank. *medRxiv* 2020.

3 Hurd MD, Martorell P, Delavande A, *et al.* Monetary costs of dementia in the United States. *N Engl J Med* 2013;**368**:1326–34.

4 Hudomiet P, Hurd MD, Rohwedder S. The relationship between lifetime out-of-pocket medical expenditures, dementia, and socioeconomic status in the US. *J Econ Ageing* 2019;**14**:100181.

5 Banks J, Marmot M, Oldfield Z, *et al.* Disease and disadvantage in the United States and in England. *Jama* 2006;**295**:2037–45.

6 Marmot MG. Status syndrome: a challenge to medicine. *Jama* 2006;**295**:1304–7.

7 Brunner EJ. Social factors and cardiovascular morbidity. *Neurosci Biobehav Rev* 2017;**74**:260–8.

8 Marmot M. Health equity in England: the Marmot review 10 years on. *Bmj* 2020;**368**.

9 Rusmaully J, Dugravot A, Moatti J-P, *et al.* Contribution of cognitive performance and cognitive decline to associations between socioeconomic factors and dementia: A cohort study. *PLoS Med* 2017;**14**:e1002334.

10 George KM, Lutsey PL, Kucharska-Newton A, *et al.* Life Course Individual and Neighborhood Socioeconomic Status and Risk of Dementia in the Atherosclerosis Risk in Communities Neurocognitive Study (ARIC-NCS). *Am J Epidemiol* 2020.

11 Cadar D, Lassale C, Davies H, *et al.* Individual and area-based socioeconomic factors associated with dementia incidence in England: evidence from a 12-year follow-up in the English longitudinal study of ageing. *JAMA Psychiatry* 2018;**75**:723–32.

12 Global Burden of Disease. Global Burden of Disease. GBD Compare. IHME Viz Hub. GBD Comp. Viz Hub. https://vizhub.healthdata.org/gbd-compare/ (accessed 18 Jul 2020).

13 Launer LJ. Statistics on the burden of dementia: need for stronger data. *Lancet Neurol* 2019;**18**:25–7.

14 Sonnega A, Faul JD, Ofstedal MB, *et al.* Cohort profile: the health and retirement study (HRS). *Int J Epidemiol* 2014;**43**:576–85.

15 Steptoe A, Breeze E, Banks J, *et al.* Cohort profile: the English longitudinal study of ageing. *Int J Epidemiol* 2013;**42**:1640–8.

16 Gianattasio KZ, Wu Q, Glymour MM, *et al.* Comparison of methods for algorithmic classification of dementia status in the health and retirement study. *Epidemiol Camb Mass* 2019;**30**:291.

17 Jorm AF, Christensen H, Korten A, *et al.* Informant ratings of cognitive decline in old age: validation against change on cognitive tests over 7 to 8 years. *Psychol Med* 2000;**30**:981–5.

18 Banks J, Muriel A, Smith JP. Attrition and health in ageing studies: evidence from ELSA and HRS. *Longitud Life Course Stud* 2011;**2**:101–26.

19 Wagstaff A, Paci P, Van Doorslaer E. On the measurement of inequalities in health. *Soc Sci Med* 1991;**33**:545–57.

For peer review only - http://bmjopen.bmj.com/site/about/guidelines.xhtml

20 Yaffe K, Falvey C, Harris TB, *et al.* Effect of socioeconomic disparities on incidence of dementia among biracial older adults: prospective study. *Bmj* 2013;**347**.

21 Vega IE, Cabrera LY, Wygant CM, *et al.* Alzheimer's disease in the latino community: Intersection of genetics and social determinants of health. *J Alzheimers Dis* 2017;**58**:979–92.

22 Langa KM, Larson EB, Crimmins EM, *et al.* A comparison of the prevalence of dementia in the United States in 2000 and 2012. *JAMA Intern Med* 2017;**177**:51–8.

23 Rocca WA, Petersen RC, Knopman DS, *et al.* Trends in the incidence and prevalence of Alzheimer's disease, dementia, and cognitive impairment in the United States. *Alzheimers Dement* 2011;**7**:80–93.

24 Chetty R, Stepner M, Abraham S, *et al.* The association between income and life expectancy in the United States, 2001-2014. *Jama* 2016;**315**:1750–66.

25 Stoye G, Zaranko B, Shipley M, *et al.* Educational inequalities in hospital use among the over 65s in England. *Milbank Q* 2020.

26 De Nardi M, French E, Jones JB, *et al.* Medical spending of the US elderly. *Fisc Stud* 2016;**37**:717–47.

27 Langa KM, Ryan LH, McCammon RJ, *et al.* The health and retirement study harmonized cognitive assessment protocol project: study design and methods. *Neuroepidemiology* 2020;**54**:64–74.

28 Nguyen TT, Tchetgen EJT, Kawachi I, *et al.* Instrumental variable approaches to identifying the causal effect of educational attainment on dementia risk. *Ann Epidemiol* 2016;**26**:71–6.

For peer review only

For peer review only - http://bmjopen.bmj.com/site/about/guidelines.xhtml

Author Licence

I *Jeremy McCauley [*The Corresponding Author] of this article contained within the original manuscript which includes any diagrams & photographs within and any related or stand alone film submitted (the Contribution") has the right to grant on behalf of all authors and does grant on behalf of all authors, a licence to the BMJ Publishing Group Ltd and its licencees, to permit this Contribution (if accepted) to be published in the BMJ and any other BMJ Group products and to exploit all subsidiary rights, as set out in our licence set out at: http://www.bmj.com/about-bmj/resources-authors/forms-policies-and-checklists/copyright-open-access-and-permission-reuse."

IF YOU ARE A NATIONAL INSTITUTE OF HEALTH ("NIH") EMPLOYEE, CONTRACTOR OR TRAINEE the following cover sheet will be accepted by the BMJ Group and NIH and incorporated into the above Licence.

Please tick **one or more** boxes as appropriate:

- ☐ I am the sole author of the Contribution.
- ✓ I am one author signing on behalf of all co-owners of the Contribution.
- ☐ The Contribution has been made in the course of my employment and I am signing as authorised by my employer.
- ☐ I am a US Federal Government employee acting in the course of my employment.
- ☐ I am not a US Federal Government employee, but some or all of my co-authors are.
- ☐ I am an employee of the UK Crown* acting in the course of my employment
- ☐ I am a US Federal Government employee acting in the course of my employment.
- ☐ I am not a US Federal Government employee, but some or all of my co-authors are.
- ☐ I am an employee of the UK Crown acting in the course of my employment
- ☐ I am not an employee of the UK Crown acting in the course of my employment but some/all of my co-authors are.*

For peer review only - http://bmjopen.bmj.com/site/about/guidelines.xhtml

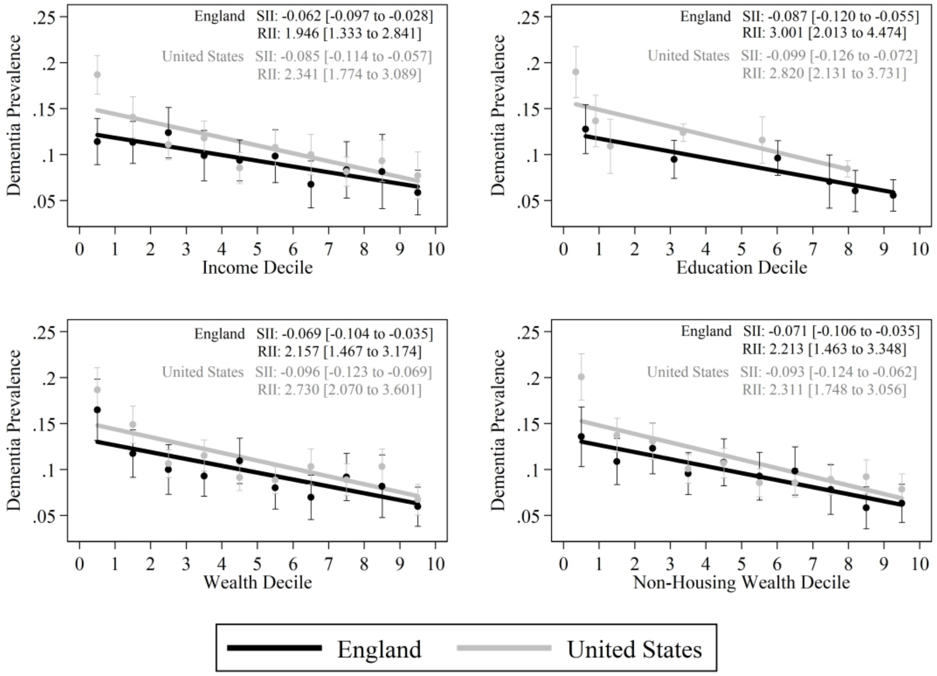

SES Gradient of Dementia, United States vs England, 2017, according to four measures of socioeconomic status. Absolute and relative inequality shown with 95% confidence intervals.

169x123mm (600 x 600 DPI)

Supplementary Appendix (For Online Publication)

Research in context

We reviewed existing evidence in July 2020, searching PubMed database for any studies looking at cross-country comparisons of dementia using the same standard measures and diagnostic practices.

("Dementia"[mesh])   AND ("Prevalence"[mesh])

AND

("Survey"[tw]) AND ("Population"[tw]) AND ("Representative"[tw])

AND

("Cross-Country"[ti] OR "Comparison"[ti])

This search yielded no results. The only relevant project we could find relating to our research was regarding the Health and Retirement Study Harmonized Cognitive Assessment Protocol (HCAP) Project. [1] This is an international research collaboration funded by the National Institute on Aging to better measure and identify cognitive impairment and dementia in longitudinal studies of countries around the world. However, this project has not yet released any international comparisons.

We also reviewed existing evidence in the PubMed database for any studies looking at how dementia prevalence varies by socioeconomic status (SES) in either England or the US. The search terms used were the following:

("Dementia"[mesh]) AND

("Prevalence"[tw])   AND ("Socioeconomic"[ti] OR "Socioeconomic"[tw] OR "SES"[tw]) AND

("United Kingdom"[mesh] OR "United Kingdom"[tw] OR "England"[tw] OR "UK"[tw] OR "United Kingdom"[tw] "Britain"[tw] OR "United States"[mesh] OR "United States"[tw] OR "US"[tw] OR "America"[tw])

This search yielded 50 results. Papers which were not relevant were manually removed. We also excluded papers that did not use nationally representative samples. We performed additional searches using lists of references retrieved from relevant papers. Relevant papers found in the search can be found in Table A1 below.

For peer review only - http://bmjopen.bmj.com/site/about/guidelines.xhtml

Table A1: Studies on Dementia Prevalence by Socioeconomic Status (SES)

Authors	Country	Socioeconomic Indicator	Years	Findings
Langa et al. (2017)[1]	United States	Education	2000 and 2012	Link between education and dementia risk
Hudomiet et al, (2019)[2]	United States	Education and Social Security Benefits	1998 to 2014	Link between education and dementia risk
Rusmaully et al, (2017)[3]	United Kingdom	Education, height, occupational position	Various years between 1985 and 2015	High cognitive reserve associated with lower risk for dementia
Cadar et al, (2018)[4]	United Kingdom	Education, wealth and the index of multiple deprivation	2002 to 2015	Lower wealth in but not education associated with increased risk for dementia
Rocca et al,(2011)[5]	United States	Education, net worth	1993 and 2002	Higher education, higher net worth protected against cognitive impairment.
Basu (2013)[6]	United States	Education	2000 - 2002	Education has causal effect on dementia risk
Nguyen et al, (2016)[7]	United States	Education	1998 to 2010	Education protective against dementia risk
Crimmins et al, (2018)[8]	United States	Education	2000 and 2010	More education linked to lower dementia prevalence
Garcia et al, (2018)[9]	United States	Race, ethnicity, nativity, and education	2012	Education reduces the odds for CIND
Weden et al, (2018)[10]	United States	Race, ethnicity, total number of children, marital status, highest educational attainment, and net total assets in 2000	2000 and 2010	Strong protective role of educational attainment and persisting rural disadvantages for dementia

For peer review only

For peer review only - http://bmjopen.bmj.com/site/about/guidelines.xhtml

Data Appendix

HRS Data

The Health and Retirement Study (HRS) is a nationally representative, biennial longitudinal survey of adults in the United States.[11] It started in 1992 and since it collects a wide range of questions on income, wealth, employment, health, cognition, and demographics. It utilises a steady-state sampling design, with a new cohort aged 51-56 entering every 6 years. In total, 43,478 individuals have been interviewed to date. We used data from the 2016 and earlier waves to predict dementia and focused on the over the age of 70 years old that live in the community or in nursing homes in 2016. This left a sample of 7,165 individuals. We restricted our attention to non-Hispanic whites for comparability with ELSA. These restrictions generated a main study sample of 5,330 participants with 4,932 being self-respondents, and 398 proxy interviews.

ELSA Data

The English Longitudinal Study of Ageing (ELSA) is a biennial longitudinal survey of adults in England, developed as a companion study to the HRS.[12] ELSA was also designed to be nationally representative of the non-institutionalised population. Respondents remain the study if they become institutionalised. While it is has been shown to be representative of the English population in terms of sociodemographic characteristics, the proportion of non-white people in the survey is very small.[12] We used data from the 2016 and earlier waves and focused on the aged over 70 years old that live in the community or in nursing homes in 2016. This left a sample of 3,224 individuals. We restricted our attention to non-Hispanic whites for comparability. These restrictions generated a study sample of 3,147 participants with 3,007 being self-respondents, and 140 proxy interviews.

Harmonised Variables HRS and ELSA

We use the same set of cognitive and demographic variables available in both the HRS and ELSA.

For cognitive and demographic measures, we used the same questions as Hurd et al.[13] Specifically, we used variables on demographics, difficulties respondents have with Activities of Daily Living (ADLs), Instrumental Activities of Daily Living (IADLs), and a range of cognitive function tests adapted from the Telephone Interview for Cognitive Status (TICS) and their change across waves. For Proxy respondents we also used the shortened 16 question form of IQCODE.

We used four variables to measure socioeconomic status (SES). These are household income, education, total household wealth, and total household non-housing wealth. For the HRS we used variables from HRS RAND dataset. To establish each respondent's SES in terms of income, wealth, and non-housing wealth, we assigned everyone to a decile rank for each variable in their respective country. For education, we ranked respondents according to their number of years of schooling. We used respondents' reported wealth and non-housing wealth from 4 years prior. If a respondent had a missing value of income, wealth, or non-housing wealth we used the most recent non-missing observation from previous waves. All amounts of income, wealth and non-housing wealth were deflated to 2016 GBPs, and USD amounts were converted to GBPs using 1 USD = 0.75 GBP.

Finally, in our analysis, we investigated how variables that have been associated with dementia affect our results. These include Body Mass Index (BMI), past smoking behaviour, past stroke. Respondents are asked whether they have any health conditions, of which we use whether they had any cardiometabolic diseases (diabetes, heart disease, and stroke) and/or psychiatric conditions 4 years prior. BMI is collected by a nurse for every ELSA respondent when they first enter the survey, which for everyone over 70 in the 2016 survey means their BMI was collected over 10 years prior. BMI is recorded in each wave of HRS based on self-reported height and weight. To make the BMI measures in HRS comparable with ELSA, we used respondents first ever recorded BMI in HRS, which for our sample was at least 10 years prior.

Hurd et al. Dementia Prediction Algorithm

To estimate the probability of dementia for each individual present in our pooled data we follow the method of Hurd et al. that is based on the HRS supplement Aging, Demographics and Memory Study (ADAMS).[13] It is a representative subsample of HRS members aged over 70 that received a detailed in-home assessment of their cognitive status by experienced teams at the Duke University Dementia Epidemiology

For peer review only - http://bmjopen.bmj.com/site/about/guidelines.xhtml

Research Center.[14] Consensus conferences were used to establish a final diagnosis of dementia or a cognitive impairment with no dementia (CIND) for each participant. Hurd et al. applied separate ordered probit models to self- and proxy-respondents to generate a predictive algorithm, based on the ADAMS diagnoses, for the whole HRS sample, using a range of variables including demographic information, ADLs, IADLs, the TICS questionnaire, IQCODE, lagged variables and differences in variables between waves. The algorithm predicts the probability of dementia in the following year.

Most of the coefficients used in the predictive algorithm can be found in the online appendix of the Hurd et al. paper. To account for the small number of missing variables in HRS and ELSA we either used coefficients provided by the authors or estimated them by a conditional minimum distance estimator using the publicly available HRS data and the predicted values of the researcher contribution Dementia Predicted Probability Files (DPPF), that follows the method of Hurd et al. To verify our predictions in HRS, we compared our predictions with the DPPF data available on the HRS website for the year 2008 and found that we accurately matched predicted dementia.

Hurd et al's predictive algorithm has been shown to have high specificity and accuracy, and generally performs well in terms of sensitivity compared to other predictive algorithms for dementia.[15]

For peer review only

Table A2: HRS and ELSA Response Rates

Year	2010	2012	2014	2016
HRS				
Wave	10	11	12	13
Response rate (%)	81.0	89.1	87.1	84.3
ELSA				
Wave	5	6	7	8
Fieldwork response rate (%)	79.1	80.2	80.1	82.4

Notes: The response rates are calculated differently in HRS and ELSA. For the HRS, the response rate includes all individuals who were determined to be eligible for HRS who completed a baseline interview.[16] For ELSA, the fieldwork response rate is the proportion of eligible survey units who participate in the research study, where 'eligible' means not having been found to be ineligible through death or moving out of Great Britain.[17]

For peer review only

For peer review only - http://bmjopen.bmj.com/site/about/guidelines.xhtml

Table A3: Summary Statistics, Raw Sample, England vs United States

	Raw Full Sample		With Population Weights	
	Non-Standardized Mean		Non-Standardized Mean	
	England	United States	England	United States
Total Sample Size	3,147	5,330	3,147	5,330
Proxy Respondents (%)	0.044	0.075	0.050	0.072
Age	78.8	80.5	79.5	79.7
Female	0.551	0.590	0.553	0.561
Married	0.580	0.517	0.584	0.536
Current Income and Wealth (£)				
Income	21,070	28,800	20,215	30,240
Wealth	280,000	210,000	270,000	230,000
Non-Housing Wealth	40,000	88,275	36,000	96,150
Wealth 4 Years Prior (£)				
Wealth	270,000	220,000	250,000	230,000
Non-Housing Wealth	42,300	96,800	35,727	100,000
Education				
Less than High School	0.362	0.174	0.424	0.161
High-school or Some College	0.473	0.573	0.444	0.560
College	0.165	0.253	0.132	0.279
Current Health				
ADLs (Out of 6)	0.510	0.583	0.561	0.548
IADLs (Out of 5)	0.348	0.513	0.411	0.482
Arthritis	0.569	0.728	0.578	0.715
Cancer	0.190	0.251	0.189	0.244
Lung disease	0.117	0.135	0.118	0.127
Diabetes	0.162	0.246	0.166	0.240
Heart Disease	0.390	0.383	0.396	0.365
High Blood Pressure	0.576	0.684	0.583	0.671
Psychiatric Condition	0.134	0.171	0.132	0.175
Stroke	0.092	0.103	0.096	0.095
Health 4 Years Prior				
ADLs	0.346	0.274	0.381	0.267
IADLs	0.159	0.228	0.185	0.219
Diabetes	0.131	0.215	0.135	0.212
Heart Disease	0.309	0.316	0.312	0.303
High Blood Pressure	0.513	0.653	0.519	0.643
Psychiatric Condition	0.120	0.157	0.118	0.162
Stroke	0.063	0.074	0.065	0.067
Other Health Factors				
Ever Smoked	0.660	0.553	0.674	0.561
BMI: <20 (Underweight)	0.016	0.038	0.015	0.036
BMI: 20-24.9 (Normal Weight)	0.266	0.352	0.262	0.343
BMI: 25-29.9 (Overweight)	0.461	0.405	0.461	0.410
BMI: 30+ (Obese)	0.256	0.205	0.262	0.211

Notes: Sample includes non-Hispanic white population aged 70+ only. The sample size is participants in England and participants in the United States. The mean is presented for each variable, apart from income and wealth variables where medians values are displayed. All amounts deflated to 2016 £s. Dollar amounts converted to £ using 1 $ = 0.75 £. Age is top coded at 99.

For peer review only

Table A4: Summary Statistics, Age-Gender Standardized, England vs United States

	Full Sample		Lowest Income Decile	
	Age-Gender Standardized Mean		Age-Gender Standardized Mean	
	England	United States	England	United States
Age	79.5	79.3	79.7	79.7
Female	0.553	0.553	0.553	0.553
Married	0.584	0.547	0.148	0.170
Current Income and Wealth (£)				
Income	20,547	31,927	8,247	8,645
Wealth	271,511	236,431	120,261	46,762
Non-Housing Wealth	38,715	100,996	7,778	5,509
Wealth 4 Years Prior (£)				
Wealth	257,459	242,655	111,830	55,953
Non-Housing Wealth	40,604	110,530	6,476	13,724
Education				
Less than High School	0.424	0.160	0.619	0.323
High-school or Some College	0.444	0.559	0.318	0.556
College	0.132	0.281	0.063	0.122
Current Health				
ADLs (Out of 6)	0.561	0.523	0.559	0.827
IADLs (Out of 5)	0.411	0.457	0.421	0.679
Arthritis	0.578	0.715	0.617	0.750
Cancer	0.189	0.244	0.157	0.196
Lung disease	0.118	0.127	0.137	0.217
Diabetes	0.166	0.242	0.157	0.283
Heart Disease	0.396	0.364	0.402	0.427
High Blood Pressure	0.583	0.671	0.619	0.703
Psychiatric Condition	0.132	0.174	0.158	0.262
Stroke	0.096	0.094	0.111	0.135
Health 4 Years Prior				
ADLs	0.381	0.255	0.424	0.546
IADLs	0.185	0.205	0.218	0.393
Diabetes	0.135	0.214	0.131	0.254
Heart Disease	0.312	0.302	0.345	0.352
High Blood Pressure	0.519	0.640	0.559	0.662
Psychiatric Condition	0.118	0.162	0.142	0.271
Stroke	0.065	0.066	0.064	0.099
Other Health Factors				
Ever Smoked	0.674	0.565	0.741	0.594
BMI: <20 (Underweight)	0.015	0.036	0.025	0.032
BMI: 20-24.9 (Normal Weight)	0.261	0.342	0.361	0.364
BMI: 25-29.9 (Overweight)	0.461	0.410	0.397	0.339
BMI: 30+ (Obese)	0.262	0.212	0.216	0.265

Notes: Sample includes non-Hispanic white population aged 70+ only. The sample size is 3,147 participants in England and 5,330 participants in the United States. The age-gender standardized mean is presented for each variable, apart from income and wealth variables where age-gender standardised medians values are displayed. The standard population is the overall age 70+ population in England in 2016. All amounts deflated to 2016 £s. Dollar amounts converted to £ using 1 $ = 0.75 £. Age is top coded at 99.

For peer review only - http://bmjopen.bmj.com/site/about/guidelines.xhtml

For peer review only

Table A5: Summary Statistics for Predictor Variables, England vs United States

	Full Sample		Lowest Income Decile	
	Age-Gender- Standardized Mean		Age-Gender- Standardized Mean	
	England	United States	England	United States
**Cognitive Scores**				
Dates (Out of 4)	3.663	3.387	3.573	3.198
Backward counting 20	0.912	0.875	0.935	0.830
Serial 7 (Out of 5)	3.787	3.364	3.667	2.580
Scissor	0.978	0.920	0.993	0.912
Cactus	0.915	0.891	0.913	0.842
PM/Vice-President	0.846	0.900	0.828	0.854
Immediate recall (Out of 10)	5.318	4.631	5.010	3.972
Delayed recall (Out of 10)	3.797	3.684	3.185	2.894
**Limitations**				
ADLs (Out of 6)	0.561	0.523	0.559	0.827
IADLs (Out of 5)	0.411	0.457	0.421	0.679
Proxy Respondent	0.050	0.068	0.038	0.075

Notes: Sample includes non-Hispanic white population aged 70+ only. The sample size is 3,147 participants in England and 5,330 participants in the United States. The Cognitive Scores are from a range of tests adapted from Telephone Interview for Cognitive Status (TICS). Only non-proxy respondents answered the TICS questions. ADL and IADLs are Activities of Daily Living and Instrumental Activities of Daily Living, respectively.

For peer review only

Table A6: Indexes of Inequality

	Slope Index of Inequality (SII)			Relative Index of Inequality (RII)		
	England	United States	Difference	England	United States	Difference
Income	-0.062	-0.085	-0.023	1.946	2.341	1.203
	[-0.097 to -0.028]	[-0.114 to -0.057]	(0.31)	[1.333 to 2.841]	[1.774 to 3.089]	(0.44)
Education	-0.087	-0.099	-0.011	3.001	2.820	0.940
	[-0.120 to -0.055]	[-0.126 to -0.072]	(0.56)	[2.013 to 4.474]	[2.131 to 3.731]	(0.80)
Wealth	-0.069	-0.096	-0.026	2.157	2.730	1.265
	[-0.104 to -0.035]	[-0.123 to -0.069]	(0.24)	[1.467 to 3.174]	[2.070 to 3.601]	(0.33)
NH Wealth	-0.071	-0.093	-0.022	2.213	2.311	1.044
	[-0.106 to -0.035]	[-0.124 to -0.062]	(0.36)	[1.463 to 3.348]	[1.748 to 3.056]	(0.87)
**Excluding Most Disadvantaged Group**						
Income	-0.067	-0.060	0.007	2.104	1.833	0.871
	[-0.107 to -0.027]	[-0.093 to -0.027]	(0.79)	[1.343 to 3.296]	[1.313 to 2.560]	(0.63)
Education	-0.070	-0.062	0.008	2.509	1.932	0.770
	[-0.107 to -0.032]	[-0.094 to -0.030]	(0.76)	[1.548 to 4.065]	[1.378 to 2.708]	(0.39)
Wealth	-0.065	-0.059	0.005	2.092	1.887	0.902
	[-0.104 to -0.025]	[-0.091 to -0.028]	(0.84)	[1.319 to 3.317]	[1.349 to 2.639]	(0.72)
NH Wealth	-0.064	-0.075	-0.012	2.077	1.981	0.954
	[-0.107 to -0.020]	[-0.110 to -0.040]	(0.69)	[1.217 to 3.544]	[1.433 to 2.740]	(0.88)
**Full Sample with Controls**						
Income	-0.040	-0.051	-0.011	1.499	1.698	1.133
	[-0.075 to -0.004]	[-0.079 to -0.023]	(0.63)	[1.028 to 2.187]	[1.275 to 2.263]	(0.61)
Education	-0.0336	-0.056	-0.022	1.308	1.742	1.332
	[-0.0754 to 0.008]	[-0.086 to -0.026]	(0.40)	[0.899 to 1.903]	[1.306 to 2.324]	(0.24)
Wealth	-0.060	-0.045	0.015	1.777	1.483	0.834
	[-0.097 to -0.023]	[-0.074 to -0.015]	(0.53)	[1.224 to 2.582]	[1.131 to 1.945]	(0.44)
NH Wealth	-0.050	-0.051	-0.002	1.562	1.614	1.033
	[-0.087 to -0.012]	[-0.080 to -0.023]	(0.94)	[1.078 to 2.262]	[1.231 to 2.116]	(0.89)

Notes: This table shows the Slope Index of Inequality (SII) and Relative Index of Inequality (RII), calculated using generalized linear models (log binomial regression) with identity and logarithmic link functions, respectively. The SIIs are displayed graphically in Figure 1. The estimates of RII are risk ratios. P-values are displayed in parentheses under differences. The panel 'Excluding Most Disadvantaged Group' excludes individuals in the bottom income decile. The panel 'Full Sample with Controls' controls for whether an individual had any cardiometabolic diseases (diabetes, heart disease, and stroke) and/or psychiatric conditions 4 years prior, whether an individual has ever smoked, and Body Mass Index (BMI) from at least 10 years prior.

For peer review only - http://bmjopen.bmj.com/site/about/guidelines.xhtml

Table A7: Difference in Prevalence of Dementia with Controls, United States vs England, 2017

Whole Sample:					
England Prevalence	0.097	0.088	0.086	0.089	0.092
	(0.089 to 0.106)	(0.078 to 0.098)	(0.073 to 0.099)	(0.072 to 0.106)	(0.072 to 0.111)
United States	0.112	0.100	0.098	0.101	0.120
	(0.106 to 0.118)	(0.092 to 0.108)	(0.087 to 0.109)	(0.087 to 0.114)	(0.104 to 0.135)
Percentage Point Difference	1.43	1.15	1.21	1.18	2.82
p-value	0.0055	0.025	0.020	0.034	<0.0001
Excluding Lowest Income Decile:					
England Prevalence	0.094	0.086	0.086	0.090	0.094
	(0.085 to 0.103)	(0.076 to 0.096)	(0.073 to 0.1)	(0.073 to 0.108)	(0.074 to 0.114)
United States	0.103	0.093	0.094	0.098	0.115
	(0.097 to 0.109)	(0.085 to 0.102)	(0.083 to 0.105)	(0.084 to 0.113)	(0.099 to 0.131)
Percentage Point Difference	0.89	0.74	0.75	0.81	2.09
p-value	0.11	0.18	0.18	0.18	0.0021
Lowest Income Decile:					
England Prevalence	0.114	0.107	0.088	0.082	0.095
	(0.089 to 0.139)	(0.074 to 0.14)	(0.036 to 0.14)	(0.021 to 0.144)	(0.025 to 0.164)
United States	0.187	0.162	0.148	0.131	0.163
	(0.166 to 0.208)	(0.129 to 0.195)	(0.103 to 0.192)	(0.078 to 0.184)	(0.103 to 0.223)
Percentage Point Difference	7.27	5.45	5.93	4.85	6.85
p-value	<0.0001	0.0020	0.0011	0.012	0.0015
Control for:					
Past Health Conditions		✓	✓	✓	✓
Ever Smoked			✓	✓	✓
Past BMI				✓	✓
Education					✓

Notes: Sample includes non-Hispanic white population aged 70+ only. The sample size is 3,147 participants in England and 5,330 participants in the United States. All estimates are age-gender standardized to the overall 2016 aged 70+ white population in England. The difference is calculated as the prevalence in the US minus prevalence in England. Differences are displayed as percentage points. 'Past Health Conditions' controls for whether an individual had any cardiometabolic diseases (diabetes, heart disease, and stroke) and/or psychiatric conditions 4 years prior. 'Ever Smoked' controls for whether an individual has ever smoked. BMI stands for Body Mass Index. 'Past BMI' includes dummy variables to control for whether an individual is classed as underweight, normal weight, *overweight* or *obese*. BMI measurements are based on when an individual first entered the survey, which is at least 10 years prior. We assume the effect of each control is constant across countries and age/gender groups. The reference groups are: No past health conditions; never smoked; normal weight; and high-school education.

For peer review only

Table A8: Difference in Prevalence of Dementia with Controls, United States vs England, 2017

	England			United States		
Whole Sample:						
Prevalence	0.097	0.092	0.089	0.112	0.105	0.101
CI	(0.089 to 0.106)	(0.082 to 0.102)	(0.072 to 0.106)	(0.106 to 0.118)	(0.098 to 0.113)	(0.087 to 0.114)
Income Decile:						
1 *Prevalence*	0.114	0.112	0.082	0.187	0.173	0.131
CI	(0.089 to 0.139)	(0.079 to 0.144)	(0.021 to 0.144)	(0.166 to 0.208)	(0.142 to 0.205)	(0.078 to 0.184)
2 *Prevalence*	0.113	0.119	0.118	0.141	0.145	0.148
CI	(0.09 to 0.136)	(0.088 to 0.149)	(0.05 to 0.187)	(0.119 to 0.163)	(0.113 to 0.176)	(0.086 to 0.21)
3 *Prevalence*	0.124	0.107	0.130	0.111	0.089	0.093
CI	(0.097 to 0.151)	(0.074 to 0.139)	(0.073 to 0.187)	(0.095 to 0.127)	(0.067 to 0.11)	(0.048 to 0.137)
4 *Prevalence*	0.099	0.095	0.094	0.118	0.114	0.111
CI	(0.071 to 0.126)	(0.065 to 0.126)	(0.041 to 0.148)	(0.099 to 0.137)	(0.088 to 0.14)	(0.062 to 0.16)
5 *Prevalence*	0.094	0.087	0.061	0.086	0.084	0.063
CI	(0.072 to 0.116)	(0.062 to 0.112)	(0.024 to 0.098)	(0.069 to 0.102)	(0.063 to 0.104)	(0.035 to 0.09)
6 *Prevalence*	0.098	0.097	0.116	0.108	0.107	0.100
CI	(0.07 to 0.127)	(0.064 to 0.131)	(0.056 to 0.177)	(0.088 to 0.128)	(0.081 to 0.134)	(0.053 to 0.148)
7 *Prevalence*	0.068	0.066	0.073	0.100	0.101	0.104
CI	(0.042 to 0.093)	(0.038 to 0.094)	(0.021 to 0.124)	(0.078 to 0.122)	(0.073 to 0.129)	(0.054 to 0.154)
8 *Prevalence*	0.083	0.084	0.097	0.082	0.078	0.085
CI	(0.053 to 0.114)	(0.051 to 0.117)	(0.054 to 0.14)	(0.066 to 0.097)	(0.058 to 0.098)	(0.054 to 0.117)
9 *Prevalence*	0.082	0.076	0.100	0.093	0.085	0.107
CI	(0.041 to 0.122)	(0.034 to 0.118)	(0.048 to 0.151)	(0.071 to 0.116)	(0.06 to 0.111)	(0.07 to 0.145)
10 *Prevalence*	0.059	0.058	0.051	0.077	0.077	0.068
CI	(0.034 to 0.083)	(0.029 to 0.087)	(0.015 to 0.087)	(0.052 to 0.103)	(0.051 to 0.103)	(0.036 to 0.1)
Control for:						
Past Health Conditions		✓	✓		✓	✓
Ever Smoked			✓			✓
Past BMI			✓			✓

Notes: Sample includes non-Hispanic white population aged 70+ only. The sample size is 3,147 participants in England and 5,330 participants in the United States. All estimates are age-gender standardized to the overall 2016 aged 70+ white population in England. 'Past Health Conditions' controls for whether an individual had any cardiometabolic diseases (diabetes, heart disease, and stroke) and/or psychiatric conditions 4 years prior. 'Ever Smoked' controls for whether an individual has ever smoked. BMI stands for Body Mass Index. 'Past BMI' includes dummy variables to control for whether an individual is classed as underweight, normal weight, *overweight* or *obese*. BMI measurements are based on when an individual first entered the survey, which is at least 10 years prior. We assume the effect of each control is constant across countries and age/gender groups. The reference groups are: No past health conditions; never smoked; and normal weight.

For peer review only

Figure A1: SES Gradient of Dementia, US vs England, Controlling for Past Health

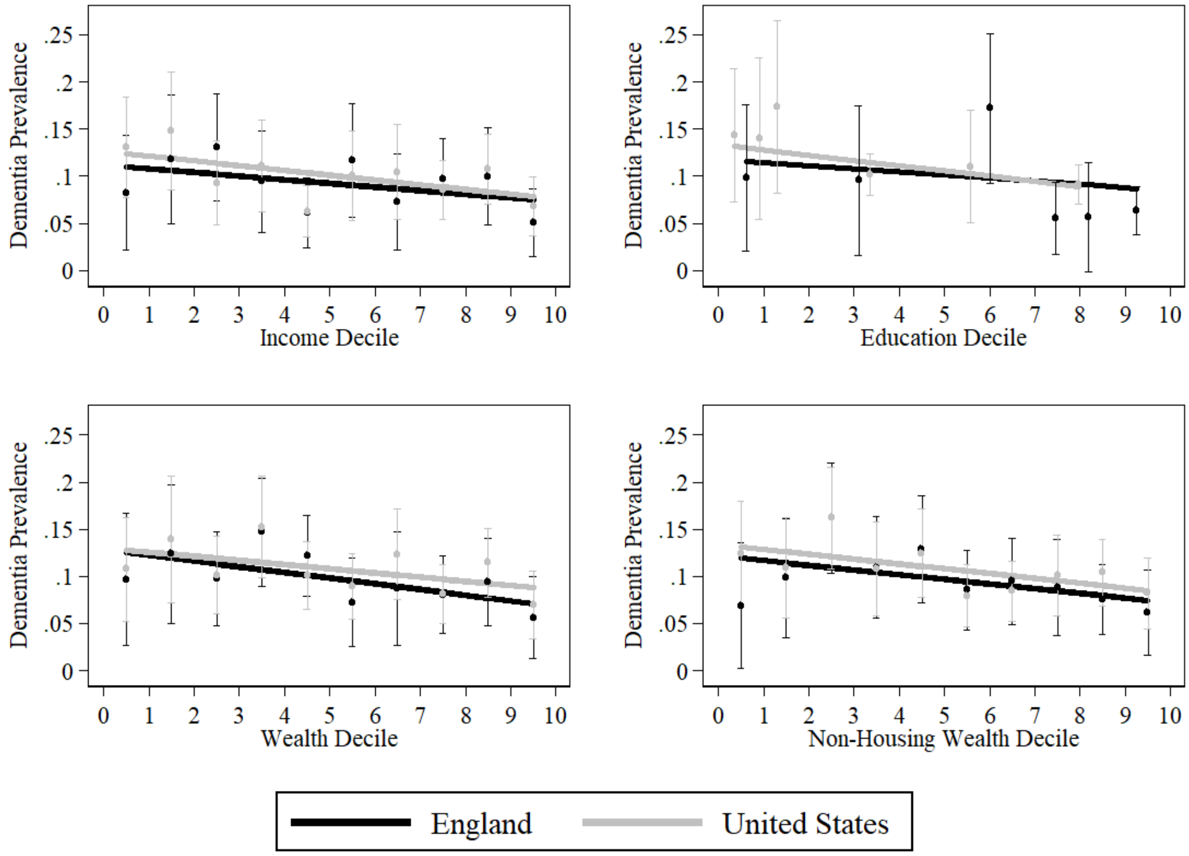

Notes: The points in this figure represent the mean age-gender standardized dementia prevalence for each country by socioeconomic status (SES) after controlling for past health and behaviour, along with 95% confidence interval for these predictions. The solid lines represent the fitted Socioeconomic Index of Inequality (SII) for each country. The values of the SII and the corresponding Relative Index of Inequality (RII) are listed in Table A4.

For peer review only - http://bmjopen.bmj.com/site/about/guidelines.xhtml

Table A9: Prevalence of Dementia, United States vs. England, Full-Sample

	England		United States			
	Age-Gender Standardized Prevalence [95% CI]		Age-Gender Standardized Prevalence [95% CI]		Difference	p-value
All	0.099	[0.091,0.108]	0.124	[0.118,0.129]	0.024	<0.0001
Household Income Decile						
1 (Lowest)	0.116	[0.091,0.140]	0.211	[0.192,0.230]	0.095	<0.0001
2	0.126	[0.101,0.152]	0.161	[0.144,0.178]	0.035	0.025
3	0.124	[0.098,0.150]	0.141	[0.120,0.162]	0.017	0.32
4	0.100	[0.072,0.127]	0.143	[0.124,0.162]	0.044	0.0094
5	0.101	[0.078,0.124]	0.097	[0.082,0.111]	-0.004	0.76
6	0.102	[0.072,0.131]	0.104	[0.086,0.121]	0.002	0.91
7	0.061	[0.043,0.079]	0.108	[0.088,0.127]	0.047	0.0006
8	0.088	[0.057,0.118]	0.085	[0.069,0.102]	-0.002	0.91
9	0.081	[0.041,0.122]	0.089	[0.070,0.108]	0.008	0.74
10 (Highest)	0.057	[0.034,0.080]	0.076	[0.055,0.098]	0.020	0.22
Years of Schooling						
9 or less	0.128	[0.103,0.153]	0.212	[0.195,0.230]	0.084	<0.0001
10	0.099	[0.078,0.119]	0.143	[0.118,0.167]	0.044	0.0072
11	0.097	[0.079,0.116]	0.119	[0.093,0.144]	0.021	0.19
12	0.069	[0.041,0.097]	0.134	[0.124,0.143]	0.064	<0.0001
13	0.063	[0.040,0.085]	0.118	[0.095,0.141]	0.055	0.00071
14 or more	0.055	[0.039,0.071]	0.086	[0.078,0.094]	0.031	0.00071
Household Wealth Decile						
1 (Lowest)	0.172	[0.136,0.207]	0.219	[0.198,0.241]	0.047	0.024
2	0.118	[0.093,0.144]	0.169	[0.151,0.188]	0.051	0.0014
3	0.105	[0.078,0.132]	0.138	[0.120,0.155]	0.032	0.049
4	0.090	[0.068,0.111]	0.120	[0.105,0.135]	0.030	0.023
5	0.118	[0.093,0.143]	0.116	[0.100,0.132]	-0.002	0.89
6	0.077	[0.056,0.099]	0.097	[0.084,0.110]	0.020	0.13
7	0.073	[0.047,0.098]	0.092	[0.076,0.107]	0.019	0.21
8	0.093	[0.067,0.118]	0.103	[0.086,0.120]	0.010	0.52
9	0.080	[0.047,0.113]	0.101	[0.084,0.118]	0.021	0.27
10 (Highest)	0.060	[0.040,0.081]	0.074	[0.058,0.089]	0.013	0.30
Household Non-Housing Wealth Decile						
1 (Lowest)	0.147	[0.113,0.180]	0.215	[0.195,0.235]	0.068	0.00055
2	0.114	[0.089,0.140]	0.184	[0.165,0.203]	0.070	<0.0001
3	0.121	[0.095,0.148]	0.148	[0.129,0.168]	0.027	0.11
4	0.100	[0.076,0.125]	0.129	[0.112,0.147]	0.029	0.054
5	0.110	[0.084,0.135]	0.104	[0.088,0.119]	-0.006	0.70
6	0.093	[0.067,0.118]	0.099	[0.085,0.113]	0.006	0.66
7	0.098	[0.072,0.124]	0.085	[0.071,0.098]	-0.013	0.37
8	0.079	[0.052,0.106]	0.090	[0.075,0.105]	0.011	0.48
9	0.057	[0.035,0.078]	0.094	[0.077,0.110]	0.037	0.0076
10 (Highest)	0.065	[0.045,0.085]	0.083	[0.067,0.099]	0.018	0.15

Notes: This table presents the estimates as Table 1 for the full sample aged 70+, including non-whites. The US sample includes 5,330 non-Hispanic white participants and 1,835 non-white participants. The England sample includes 3,147 non-Hispanic white participants and 77 non-white participants. All estimates are age-gender standardized to the overall 2016 aged 70+ white population in England. The difference is calculated as the prevalence in the US minus prevalence in England.

For peer review only - http://bmjopen.bmj.com/site/about/guidelines.xhtml

Table A10: Difference in Prevalence of Dementia with Controls, Full Sample

Whole Sample:					
England Prevalence	0.099	0.088	0.088	0.084	0.085
	[0.091 to 0.108]	[0.079 to 0.098]	[0.076 to 0.1]	[0.068 to 0.1]	[0.068 to 0.103]
United States	0.124	0.107	0.107	0.105	0.123
	[0.118 to 0.129]	[0.1 to 0.114]	[0.097 to 0.117]	[0.093 to 0.118]	[0.109 to 0.137]
Percentage Point Difference	2.44	1.87	1.90	2.14	3.80
p-value	<0.0001	<0.0001	0.00015	0.00011	<0.0001
Excluding Lowest Income Decile:					
England Prevalence	0.096	0.086	0.086	0.087	0.090
	[0.087 to 0.105]	[0.076 to 0.096]	[0.073 to 0.099]	[0.071 to 0.103]	[0.072 to 0.108]
United States	0.113	0.098	0.099	0.100	0.117
	[0.107 to 0.118]	[0.091 to 0.106]	[0.089 to 0.108]	[0.086 to 0.113]	[0.103 to 0.132]
Percentage Point Difference	1.64	1.25	1.27	1.24	2.70
p-value	0.0024	0.020	0.019	0.030	<0.0001
Lowest Income Decile:					
England Prevalence	0.116	0.107	0.106	0.078	0.099
	[0.091 to 0.140]	[0.077 to 0.138]	[0.062 to 0.15]	[0.026 to 0.13]	[0.036 to 0.162]
United States	0.211	0.187	0.186	0.161	0.195
	[0.192 to 0.230]	[0.16 to 0.214]	[0.149 to 0.223]	[0.117 to 0.206]	[0.138 to 0.252]
Percentage Point Difference	9.54	7.93	7.96	8.37	9.60
p-value	<0.0001	<0.0001	<0.0001	<0.0001	<0.0001
Control for:					
Past Health Conditions		✓	✓	✓	✓
Ever Smoked			✓	✓	✓
Past BMI				✓	✓
Education					✓

Notes: Full sample, population aged 70+, including minorities. The sample sizes are 3,224 participants in England, and 7,165 participants in the United States. All estimates are age-gender standardized to the overall 2016 aged 70+ white population in England. The difference is calculated as the prevalence in the US minus prevalence in England. Differences are displayed as percentage points. 'Past Health Conditions' controls for whether an individual had any cardiometabolic diseases (diabetes, heart disease, and stroke) and/or psychiatric conditions 4 years prior. 'Ever Smoked' controls for whether an individual has ever smoked. BMI stands for Body Mass Index. 'Past BMI' includes dummy variables to control for whether an individual is classed as underweight, normal weight, *overweight* or *obese*. BMI measurements are based on when an individual first entered the survey, which is at least 10 years prior. We assume the effect of each control is constant across countries and age/gender groups. The reference groups are: No past health conditions; never smoked; normal weight; and high-school education.

For peer review only - http://bmjopen.bmj.com/site/about/guidelines.xhtml

Table A11: Prevalence of Dementia in United States, Whites versus Non-whites

	US Non-White and Hispanic		US Non-Hispanic White			
	Age-Gender Standardized Prevalence [95% CI]		Age-Gender Standardized Prevalence [95% CI]		Difference	p-value
All	0.182	[0.169 to 0.195]	0.112	[0.106 to 0.118]	-0.070	<0.0001
Household Income Decile						
1 (Lowest)	0.224	[0.199 to 0.249]	0.204	[0.178 to 0.231]	-0.020	0.28
2	0.211	[0.180 to 0.243]	0.143	[0.123 to 0.163]	-0.068	0.00034
3	0.220	[0.158 to 0.282]	0.121	[0.102 to 0.140]	-0.099	0.0023
4	0.202	[0.165 to 0.240]	0.133	[0.111 to 0.155]	-0.069	0.0018
5	0.116	[0.076 to 0.156]	0.094	[0.079 to 0.109]	-0.022	0.31
6	0.164	[0.097 to 0.230]	0.098	[0.081 to 0.116]	-0.065	0.065
7	0.129	[0.080 to 0.178]	0.107	[0.086 to 0.128]	-0.022	0.42
8	0.107	[0.047 to 0.166]	0.084	[0.067 to 0.101]	-0.023	0.47
9	0.072	[0.026 to 0.118]	0.090	[0.069 to 0.110]	0.017	0.50
10 (Highest)	0.084	[0.038 to 0.129]	0.077	[0.055 to 0.099]	-0.006	0.81
Years of Schooling						
9 or less	0.235	[0.212 to 0.258]	0.190	[0.162 to 0.218]	-0.045	0.015
10	0.172	[0.127 to 0.218]	0.137	[0.109 to 0.165]	-0.035	0.19
11	0.144	[0.109 to 0.179]	0.109	[0.080 to 0.139]	-0.035	0.14
12	0.198	[0.170 to 0.227]	0.124	[0.114 to 0.133]	-0.074	<0.0001
13	0.159	[0.101 to 0.218]	0.116	[0.090 to 0.141]	-0.043	0.18
14 or more	0.099	[0.079 to 0.119]	0.085	[0.076 to 0.093]	-0.015	0.20
Household Wealth Decile						
1 (Lowest)	0.228	[0.201 to 0.255]	0.208	[0.176 to 0.240]	-0.020	0.36
2	0.208	[0.179 to 0.237]	0.154	[0.131 to 0.177]	-0.054	0.0043
3	0.184	[0.142 to 0.227]	0.120	[0.102 to 0.138]	-0.064	0.0063
4	0.142	[0.111 to 0.174]	0.115	[0.098 to 0.132]	-0.027	0.13
5	0.159	[0.117 to 0.202]	0.108	[0.092 to 0.124]	-0.051	0.027
6	0.120	[0.084 to 0.155]	0.093	[0.080 to 0.107]	-0.026	0.18
7	0.156	[0.086 to 0.226]	0.088	[0.072 to 0.103]	-0.068	0.063
8	0.197	[0.134 to 0.259]	0.099	[0.081 to 0.116]	-0.098	0.0031
9	0.122	[0.065 to 0.178]	0.099	[0.082 to 0.117]	-0.022	0.46
10 (Highest)	0.045	[0.018 to 0.073]	0.075	[0.059 to 0.090]	0.029	0.071
Household Non-Housing Wealth Decile						
1 (Lowest)	0.231	[0.207 to 0.256]	0.197	[0.167 to 0.227]	-0.034	0.085
2	0.204	[0.177 to 0.231]	0.177	[0.151 to 0.203]	-0.027	0.16
3	0.187	[0.142 to 0.232]	0.135	[0.114 to 0.155]	-0.052	0.036
4	0.158	[0.128 to 0.188]	0.123	[0.104 to 0.143]	-0.034	0.059
5	0.106	[0.069 to 0.143]	0.102	[0.086 to 0.118]	-0.004	0.85
6	0.099	[0.063 to 0.134]	0.098	[0.084 to 0.113]	0.000	0.99
7	0.124	[0.066 to 0.182]	0.083	[0.069 to 0.097]	-0.040	0.18
8	0.140	[0.069 to 0.210]	0.088	[0.073 to 0.103]	-0.051	0.16
9	0.127	[0.054 to 0.201]	0.092	[0.075 to 0.108]	-0.035	0.36
10 (Highest)	0.069	[0.050 to 0.088]	0.084	[0.068 to 0.101]	0.016	0.23

Notes: The sample includes 5,330 non-Hispanic white participants and 1,835 non-white and Hispanic participants. All estimates are age-gender standardized to the overall 2016 aged 70+ white population in England. The difference is calculated as the non-white and Hispanic prevalence in the US minus the non-Hispanic white prevalence in the US.

For peer review only - http://bmjopen.bmj.com/site/about/guidelines.xhtml

Appendix References

1 Langa KM, Larson EB, Crimmins EM, *et al.* A comparison of the prevalence of dementia in the United States in 2000 and 2012. *JAMA Intern Med* 2017;**177**:51–8.

2 Hudomiet P, Hurd MD, Rohwedder S. The relationship between lifetime out-of-pocket medical expenditures, dementia, and socioeconomic status in the US. *J Econ Ageing* 2019;**14**:100181.

3 Rusmaully J, Dugravot A, Moatti J-P, *et al.* Contribution of cognitive performance and cognitive decline to associations between socioeconomic factors and dementia: A cohort study. *PLoS Med* 2017;**14**:e1002334.

4 Cadar D, Lassale C, Davies H, *et al.* Individual and area-based socioeconomic factors associated with dementia incidence in England: evidence from a 12-year follow-up in the English longitudinal study of ageing. *JAMA Psychiatry* 2018;**75**:723–32.

5 Rocca WA, Petersen RC, Knopman DS, *et al.* Trends in the incidence and prevalence of Alzheimer's disease, dementia, and cognitive impairment in the United States. *Alzheimers Dement* 2011;**7**:80–93.

6 Basu R. Education and dementia risk: results from the Aging Demographics and Memory Study. *Res Aging* 2013;**35**:7–31.

7 Nguyen TT, Tchetgen EJT, Kawachi I, *et al.* Instrumental variable approaches to identifying the causal effect of educational attainment on dementia risk. *Ann Epidemiol* 2016;**26**:71–6.

8 Crimmins EM, Kim JK, Langa KM, *et al.* Assessment of cognition using surveys and neuropsychological assessment: the Health and Retirement Study and the Aging, Demographics, and Memory Study. *J Gerontol B Psychol Sci Soc Sci* 2011;**66**:i162–71.

9 Garcia MA, Saenz J, Downer B, *et al.* The role of education in the association between race/ethnicity/nativity, cognitive impairment, and dementia among older adults in the United States. *Demogr Res* 2018;**38**:155.

10 Weden MM, Shih RA, Kabeto MU, *et al.* Secular trends in dementia and cognitive impairment of US rural and urban older adults. *Am J Prev Med* 2018;**54**:164–72.

11 Sonnega A, Faul JD, Ofstedal MB, *et al.* Cohort profile: the health and retirement study (HRS). *Int J Epidemiol* 2014;**43**:576–85.

12 Steptoe A, Breeze E, Banks J, *et al.* Cohort profile: the English longitudinal study of ageing. *Int J Epidemiol* 2013;**42**:1640–8.

13 Hurd MD, Martorell P, Delavande A, *et al.* Monetary costs of dementia in the United States. *N Engl J Med* 2013;**368**:1326–34.

14 Langa KM, Plassman BL, Wallace RB, *et al.* The Aging, Demographics, and Memory Study: study design and methods. *Neuroepidemiology* 2005;**25**:181–91.

15 Gianattasio KZ, Wu Q, Glymour MM, *et al.* Comparison of methods for algorithmic classification of dementia status in the health and retirement study. *Epidemiol Camb Mass* 2019;**30**:291.

16 Staff H. Sample sizes and response rates. *Ann Arbor MI Surv Res Cent Inst Soc Res Univ Mich Online Verfügbar Unter Httpshrs Isr Umich Edusitesdefaultf IlesbiblioResponseRates2017 Pdf Zugriff Am* 2017;**28**.

17 Banks J, Nazroo J, Steptoe A. Wave 6. *Inst Fisc Stud* 2014.

For peer review only

For peer review only - http://bmjopen.bmj.com/site/about/guidelines.xhtml

STROBE (Strengthening The Reporting of OBservational Studies in Epidemiology) Checklist

A checklist of items that should be included in reports of observational studies. You must report the page number in your manuscript where you consider each of the items listed in this checklist. If you have not included this information, either revise your manuscript accordingly before submitting or note N/A.

Note: An Explanation and Elaboration article discusses each checklist item and gives methodological background and published examples of transparent reporting. The STROBE checklist is best used in conjunction with this article (freely available on the Web sites of PLoS Medicine at http://www.plosmedicine.org/, Annals of Internal Medicine at http://www.annals.org/, and Epidemiology at http://www.epidem.com/). Information on the STROBE Initiative is available at www.strobe-statement.org.

Section and Item	Item No.	Recommendation	Reported on Page No.
**Title and Abstract**	1	(*a*) Indicate the study's design with a commonly used term in the title or the abstract	1
		(*b*) Provide in the abstract an informative and balanced summary of what was done and what was found	1
**Introduction**			
Background/Rationale	2	Explain the scientific background and rationale for the investigation being reported	3
Objectives	3	State specific objectives, including any prespecified hypotheses	3
**Methods**			
Study Design	4	Present key elements of study design early in the paper	3-4
Setting	5	Describe the setting, locations, and relevant dates, including periods of recruitment, exposure, follow-up, and data collection	3-4
Participants	6	(*a*) *Cohort study*—Give the eligibility criteria, and the sources and methods of selection of participants. Describe methods of follow-up	4
		Case-control study—Give the eligibility criteria, and the sources and methods of case ascertainment and control selection. Give the rationale for the choice of cases and controls	not applicable
		Cross-sectional study—Give the eligibility criteria, and the sources and methods of selection of participants	not applicable
		(*b*) *Cohort study*—For matched studies, give matching criteria and number of exposed and unexposed	not applicable
		Case-control study—For matched studies, give matching criteria and the number of controls per case	not applicable
Variables	7	Clearly define all outcomes, exposures, predictors, potential confounders, and effect modifiers. Give diagnostic criteria, if applicable	4-5

For peer review only

Section and Item	Item No.	Recommendation	Reported on Page No.
Data Sources/ Measurement	8*	For each variable of interest, give sources of data and details of methods of assessment (measurement). Describe comparability of assessment methods if there is more than one group	3-5
Bias	9	Describe any efforts to address potential sources of bias	4-5
Study Size	10	Explain how the study size was arrived at	4
Quantitative Variables	11	Explain how quantitative variables were handled in the analyses. If applicable, describe which groupings were chosen and why	4-5
Statistical Methods	12	(*a*) Describe all statistical methods, including those used to control for confounding	4-5
		(*b*) Describe any methods used to examine subgroups and interactions	5
		(*c*) Explain how missing data were addressed	4-5
		(*d*) *Cohort study*—If applicable, explain how loss to follow-up was addressed	4-5
		Case-control study—If applicable, explain how matching of cases and controls was addressed	not applicable
		Cross-sectional study—If applicable, describe analytical methods taking account of sampling strategy	not applicable
		(*e*) Describe any sensitivity analyses	5
**Results**			
Participants	13*	(a) Report numbers of individuals at each stage of study—eg numbers potentially eligible, examined for eligibility, confirmed eligible, included in the study, completing follow-up, and analysed	4,10
		(b) Give reasons for non-participation at each stage	not applicable
		(c) Consider use of a flow diagram	not applicable
Descriptive Data	14*	(a) Give characteristics of study participants (eg demographic, clinical, social) and information on exposures and potential confounders	4,10
		(b) Indicate number of participants with missing data for each variable of interest	not applicable
		(c) *Cohort study*—Summarise follow-up time (eg, average and total amount)	4
Outcome Data	15*	*Cohort study*—Report numbers of outcome events or summary measures over time	10-11
		Case-control study—Report numbers in each exposure category, or summary measures of exposure	not applicable
		Cross-sectional study—Report numbers of outcome events or summary measures	not applicable

For peer review only

Section and Item	Item No.	Recommendation	Reported on Page No.
Main Results	16	(*a*) Give unadjusted estimates and, if applicable, confounder-adjusted estimates and their precision (eg, 95% confidence interval). Make clear which confounders were adjusted for and why they were included	10-11
		(*b*) Report category boundaries when continuous variables were categorized	not applicable
		(*c*) If relevant, consider translating estimates of relative risk into absolute risk for a meaningful time period	6,11
Other Analyses	17	Report other analyses done—eg analyses of subgroups and interactions, and sensitivity analyses	7,10-11
**Discussion**			
Key Results	18	Summarise key results with reference to study objectives	6-7
Limitations	19	Discuss limitations of the study, taking into account sources of potential bias or imprecision. Discuss both direction and magnitude of any potential bias	8-9
Interpretation	20	Give a cautious overall interpretation of results considering objectives, limitations, multiplicity of analyses, results from similar studies, and other relevant evidence	7-8
Generalisability	21	Discuss the generalisability (external validity) of the study results	8
**Other Information**			
Funding	22	Give the source of funding and the role of the funders for the present study and, if applicable, for the original study on which the present article is based	12

*Give information separately for cases and controls in case-control studies and, if applicable, for exposed and unexposed groups in cohort and cross-sectional studies.

Once you have completed this checklist, please save a copy and upload it as part of your submission. DO NOT include this checklist as part of the main manuscript document. It must be uploaded as a separate file.

For peer review only
